# A plasmid toolbox for controlled gene expression across the Proteobacteria

**DOI:** 10.1093/nar/gkab496

**Published:** 2021-06-14

**Authors:** Layla A Schuster, Christopher R Reisch

**Affiliations:** Dept. of Microbiology and Cell Science, Institute of Food and Agricultural Sciences, University of Florida, Gainesville, FL 32603, USA; Dept. of Microbiology and Cell Science, Institute of Food and Agricultural Sciences, University of Florida, Gainesville, FL 32603, USA

## Abstract

Controlled gene expression is fundamental for the study of gene function and our ability to engineer bacteria. However, there is currently no easy-to-use genetics toolbox that enables controlled gene expression in a wide range of diverse species. To facilitate the development of genetics systems in a fast, easy, and standardized manner, we constructed and tested a plasmid assembly toolbox that will enable the identification of well-regulated promoters in many Proteobacteria and potentially beyond. Each plasmid is composed of four categories of genetic parts (i) the origin of replication, (ii) resistance marker, (iii) promoter-regulator and (iv) reporter. The plasmids can be efficiently assembled using ligation-independent cloning, and any gene of interest can be easily inserted in place of the reporter. We tested this toolbox in nine different Proteobacteria and identified regulated promoters with over fifty-fold induction range in eight of these bacteria. We also constructed variant libraries that enabled the identification of promoter-regulators with varied expression levels and increased inducible fold change relative to the original promoter. A selection of over 50 plasmids, which contain all of the toolbox's genetic parts, are available for community use and will enable easy construction and testing of genetics systems in both model and non-model bacteria.

## INTRODUCTION

Genetic tools to control gene expression commonly consist of an allosteric transcription factor that can bind regulatory DNA near a controlled promoter to initiate or repress transcriptional initiation. The addition of a small molecule ligand binds the transcription factor and enables RNA polymerase binding to the promoter for transcriptional initiation (1). These promoter-regulator pairs enable finely tuned genetic control in a few well-studied bacteria (2–5), facilitating research in areas such as essential gene analysis, metabolic pathway optimization and biosensor development (6–8). An ideal system has a large dynamic range of expression, providing a tunable response where promoter output correlates positively with the concentration of inducer added (9). Hindering this fine-tuning ability is expression in the absence of inducer, often referred to as ‘leakiness’ (10,11). Low leakiness is important for greater predictability of the system and avoids the consequences of unintended low-level expression that can obfuscate physiological experiments, allow the buildup of toxic proteins, or lower product yields in metabolic engineering (12–14). While a high dynamic range is often desired, in practice, inducible promoters often have a tight off-state but only middling on-state, or have a leaky off-state but very high on-state (15,16).

The ability to dynamically control gene expression in *Escherichia coli* is very well-developed, with at least 10 promoter-regulator pairs that can operate orthogonally with high dynamic-range (2). Other well-studied bacteria have smaller but still reliable toolboxes (4,5,17–19). The Standard European Vector Architecture (SEVA) toolbox is well-designed and possesses genetic parts for a broad-range of bacteria (20). However, the complete toolbox is not widely available and adding parts to the system requires re-coding to remove incompatible restriction sites. Moreover, this toolbox was not explicitly designed for controlled gene expression. While a limited number of regulatory proteins are available, including both a regulatory system and reporter (or gene of interest, GOI) requires additional design and cloning because each element is not inherent in the design scheme. Modular cloning (MoClo) toolboxes are also well-developed and highly customizable with hierarchical Golden Gate cloning schemes (21,22). Construction of these plasmids requires several steps with intermediate plasmids and compatible restriction sites must be available in each genetic part, limiting the speed and ease of making new vectors. A full comparison between toolboxes can be found in Supplementary Note 1. Recent work has combined the SEVA and MoClo standards for increased flexibility and cloning efficiency (22,23). Included in these toolboxes are some tried and tested genetic parts from *E. coli* but determining whether these parts function in other bacteria often requires tedious de-novo cloning and testing (24,25). While these previously built systems are valuable and contribute to the goals of parts standardization in synthetic biology, we believe that an easy-to-use toolbox for controlled gene expression is also needed.

## MATERIALS AND METHODS

### Plasmid construction

Plasmids were assembled using NEB HiFi Assembly with PCR amplified genetic parts. A detailed protocol for plasmid assembly is provided in Supplementary Note 2 and the source of genetic parts is in Supplementary Table S2. Sequences of verified plasmids are available at Addgene as indicated in Supplementary Table S1.

### Plasmid transformations

All recipient strains except for *Acinetobacter baylyi* and *Aliivibrio fischeri* were transformed via electroporation. The cell cultures made electrocompetent were taken from either an overnight growth (*Burkholderia thailandensis*, *Pseudomonas aeruginosa*, *Pseudomonas putida* and *Xanthomonas campestris*) or subcultured from an overnight growth and made electrocompetent when the cells reached mid-log phase (*Agrobacterium fabrum*, *Ruegeria* sp. TM1040, *Sulfitobacter* sp. EE-36). The protocol for the preparation of electrocompetent cells was as follows: 6 ml of each wild-type strain was incubated with shaking in the indicated media and temperature conditions (Supplementary Table S3) overnight or until mid-log phase. The total culture was then spun down in four 1.5 ml tubes in a microcentrifuge at 5000 rpm for 2 min. Culture supernatants were aspirated, cell pellets resuspended in 1 ml 300 mM sucrose at room temperature and then centrifuged again for 2 min at 5000 rpm. This process was repeated to wash with sucrose twice and then the pellet was resuspended in a final volume of 1:10 of the initial culture volume or 150 μl in each of the four microcentrifuge tubes. 50 μl of each suspension was then transferred to a 1-mm-gap-width electroporation cuvette and cells were electroporated at the specified voltage for each strain (Supplementary Table S4). Cells were recovered in 1 ml of their respective recovery media and incubated with shaking at either 30 or 37°C in a deep-well plate for 2 h before plating.

A natural transformation protocol was followed for *A. baylyi*, adapted from a previous protocol (26). Here, 5 ml of fresh LB was inoculated with wild-type ADP1 from a glycerol stock and grown overnight at 30°C. The next day, 1 ml of fresh LB was inoculated with 70 μl of this culture and approximately 100 ng of the plasmid were incubated for 3 h before plating onto selection plates.

Conjugation was performed to introduce plasmids into *A. fischeri* using the RP4 system in the following steps. On the day prior to conjugation, 5 ml cultures were inoculated from glycerol stocks of wild-type *A. fischeri*, all requiring donor strains of *E. coli*, and an *E. coli* helper strain containing pEVS104, and grown overnight. The following day, donor and helper cultures were spun down separately at 10 000 rpm for 1 min and resuspended in LBS to remove residual antibiotic. A sufficient volume of donor and helper cultures was pelleted and resuspended such that each conjugation used 500 μl of both donor and helper strains in addition to 500 μl of recipient *A. fischeri*. Each mixture of donor, helper, and recipient was centrifuged at 10 000 rpm for 1 min and the supernatant decanted, leaving approximately 100 μl of LBS to resuspend the pellet. The resuspensions were spotted on an LBS plate and incubated on the benchtop overnight. Each spot was streaked on to a fresh marine media plate containing the appropriate antibiotic the following day.

For transformation efficiency assays, 100 ng of each plasmid was transformed following the protocols above. The recovered cultures were serially diluted 10-fold four-times, and 10 μl of each dilution was spotted onto an agar plate with the appropriate antibiotic and incubated for 1–3 days until colonies were visible. For fluorescence assays, plasmids were transformed with the same protocol and after recovery, cultures were streaked onto agar plates with the appropriate antibiotic. Isolated colonies were then grown up and saved as glycerol stocks.

### Fluorescence assays

For each set of promoter-regulator pairs, glycerol stocks of all relevant strains were struck out on to fresh agar plates to obtain isolated colonies. The following day, a deep-well plate containing 1 ml of rich medium and the appropriate antibiotic was inoculated with isolated colonies and incubated on a plate shaker overnight. After approximately 20 h of growth, cultures were subcultured to an OD of 0.1 into fresh media and antibiotic and incubated on a plate shaker until the cultures reached mid-log phase. Cultures were then diluted again to an OD of 0.07 into 96-well plates (Costar, black, clear-bottom); where the wells contained 100 μl of rich media with antibiotic or rich media only, for strains with plasmids and wild-type strains, respectively. At least eight wells on each 96-well plate were not inoculated and used as controls. Plates were then incubated on a plate shaker for 0.5 h. An additional 100 μl of the respective media with 2× inducer concentration was added to each well of the plate so that the final concentration was 1× for induced samples (inducer concentrations in Supplementary Table S5). The plate was then incubated on a plate shaker, and OD and fluorescence were measured at 2, 4, 6 and 24 h post-induction on a plate reader (Molecular Devices SpectraMax M3), with an additional timepoint taken at 8 h for slower-growing strains. Fluorescence readings were taken using a plate adapter and top-read settings on the plate reader. All experiments were performed with three technical replicates and with two to three independent experiments.

All calculations and data analysis were performed using Microsoft Excel. For screens of the 12 inducible systems within each bacterial strain, absorbance and fluorescence data were organized by timepoint. Optical density was adjusted to a 1 cm pathlength by dividing by a factor of 0.56 or 0.28 when the culture volume in the well was 200 μl or 100 μl, respectively. This adjustment is applicable when the wells of a 96-well plate are completely flat and was empirically validated in our lab. In addition, when optical density measurements were above the threshold for linearity (approximately OD = 1.0 on our machine), cultures were diluted 1:10 into a total of 100 μl/well in another 96-well plate and measured for a more accurate reading. Fluorescence data taken after removing culture for these OD readings were adjusted so that data was consistent across timepoints. The raw fluorescence data was either used directly or further modified by normalizing the adjusted optical density and subtracting the fluorescence of an empty vector control included in each screen. Fold change was then calculated for the raw and modified fluorescence data.

### Antibiotic assays

The assay was adapted from a previous protocol (7). Strains were grown and diluted following a similar protocol to that outlined for the fluorescence assays. Specifically, three individual colonies were grown up from a freshly streaked agar plate in 1 ml nutrient-rich broth with the appropriate antibiotics and incubated overnight in a deep-well plate with shaking. The cultures were then diluted into 1 ml of fresh media to an OD of 0.1 and grown to mid-exponential phase. The cultures were then diluted again into 1 ml of fresh media to an OD of 0.07 with each of the three cultures inoculating two additional cultures, one that remained uninduced and one in which inducer was added. These cultures were grown for 0.5 h, at which point inducer was added to three of the six cultures, marking time zero for the spot dilution plating. Spot dilution plating occurred when cultures were in mid-exponential phase of growth and 200 μl were taken from each of the six cultures and serially diluted 10-fold in water. 10 μl of each dilution, including the undiluted culture, were spotted on to agar plates in the following way: the cultures that were grown in the absence of inducer were spotted on to LB agar with kanamycin to obtain a colony count of the total viable transformants and LB agar with gentamicin where only transformants with leaky expression would grow. The induced cultures were spotted onto the same selection plates as specified above that also contained inducer (cumate) spread at a 1x concentration to preserve induction. Spots were grown overnight or until the appearance of colonies. For *B. thailandensis*, a gentamicin concentration of 20 μg/ml was used, which is inhibitory to wild-type cells. Alongside the experimental strains, wild-type strains were grown and diluted as described above in parallel and plated on to LSLB gentamicin plates to confirm the lack of visible colonies. To make the graph in Figure 5, CFUs within the countable range were recorded on all plates and counts from the gentamicin selection plates were normalized to those on the kanamycin selection plates. The same was done for the set of plates spotted with induced cultures. Images of the full plates used to make Figure 5 are shown in Supplementary Figure S20.

### Library construction and screening

Libraries were assembled using NEB HiFi Assembly with PCR amplified genetic parts. A list of primers and a description of the construction is provided in Supplementary Note 5. Protocols for amplification and assembly are provided in Supplementary Note 2.

Library mutants were screened in two steps where the initial screen was a simplified version of the protocol followed for fluorescence assays that did not include subculturing. Library mutant transformants were inoculated by hand or using a QPix2 colony picker into 96 or 384-well plates depending on library size, and plates were incubated overnight on a plate shaker. Each plate also contained at least three wells inoculated with the original plasmid control strain and at least three wells were left as blanks. The following day, overnight cultures were stamped into two additional plates with a plate replicator, one with inducer added to the media and one without. These plates were incubated overnight, and optical density and fluorescence readings were taken the following day. Data from this screen served to identify non-functional library mutants and those that displayed a dynamic range of expression similar to or better than the control. These potentially improved mutants were isolated from the screening plate by streaking onto fresh agar plates and were included in the next screen. This second screen follows the full fluorescence assay protocol of subculturing and induction with both the original plasmid control strain and empty-vector control strain included in each plate. OD and fluorescence readings were taken during exponential phase and at 24 h post-induction. Library mutants that were characterized to be an improvement on the original plasmid control were Sanger sequenced.

### Violacein experiments

Cultures were started from single colonies and grown overnight in a deep-well plate. The following day, cultures were set back following the protocol outlined in the Fluorescence Assays section. After the final subculture, violacein was extracted from cultures in mid-log phase and after an overnight growth. Violacein extractions were done using a protocol adapted from previous protocols (27,28) and were as follows: for each measurement, 1 mL of culture was pelleted at 21 000 × g for 10 min. The supernatant was removed and the pellet was resuspended in a methanol solution containing 1% (v/v) acetic acid. Tubes were then incubated at 58°C for 10 min with periodic vortexing to extract the violacein followed by centrifugation to pellet cell debris (21 000 × g, 10 min). Multiple extractions were required from samples producing a high amount of violacein and background-subtracted measurements were summed during data analysis. From the supernatant, 200 μl was aliquoted into a 96-well and absorbance was read at 585 nm.

## RESULTS

### Plasmid design

**Figure 1.**
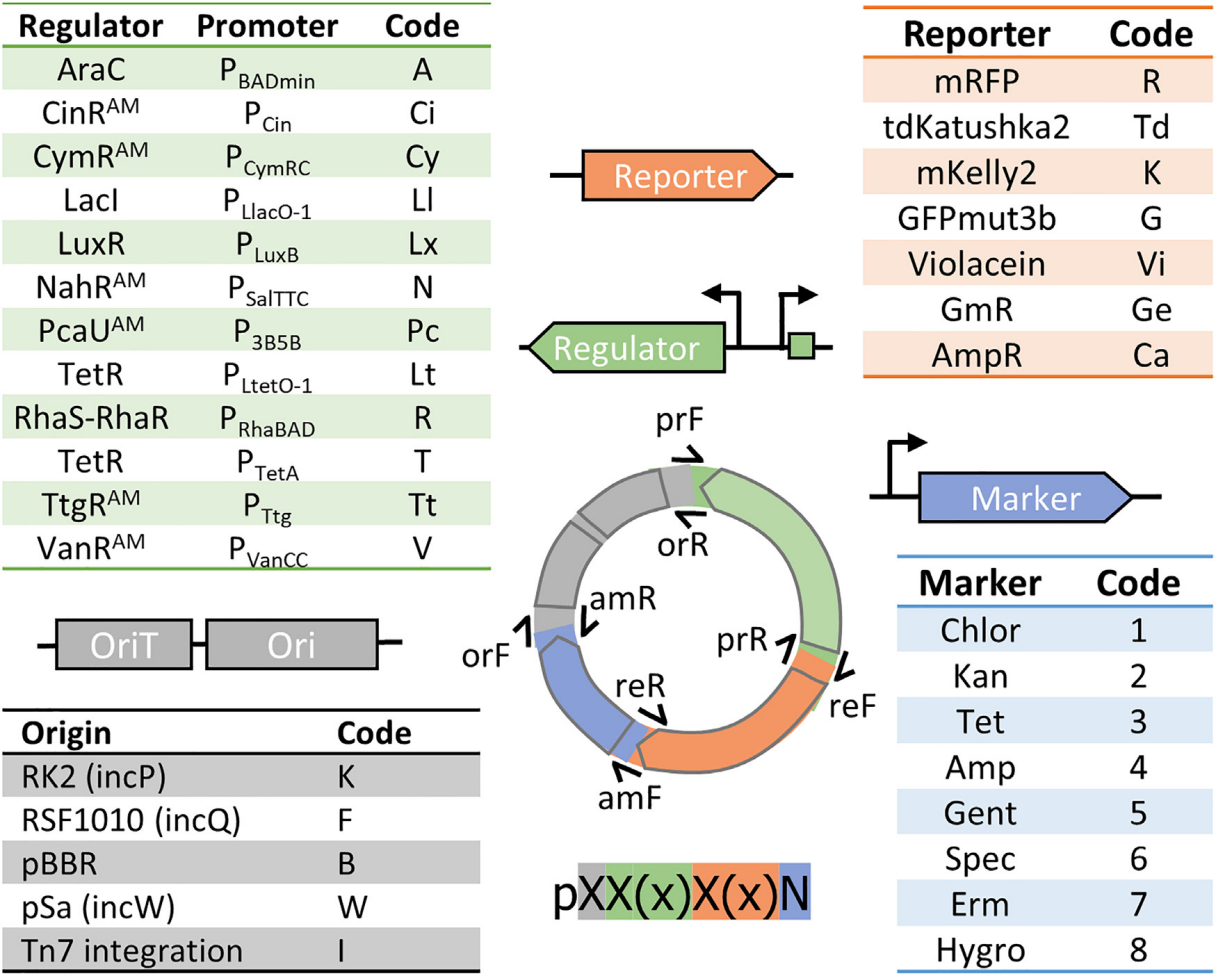
Plasmid toolbox assembly scheme and nomenclature. Each plasmid is composed of four genetic parts that share overlapping primer sequences, requiring only four primer pairs to assemble any version of the plasmid. Plasmids are named based on the codes provided, in the order: origin, regulator, reporter and marker.

To enable quick and easy assembly of customized plasmids, we developed a combinatorial strategy to construct plasmids compatible with a broad-range of bacteria that possess inducible expression systems, as outlined in Figure 1. Our assembly scheme uses ligation-independent cloning, which requires a unique primer pair for each part's initial cloning into the vector. The 3′ end of the primers anneal to the new part, and the 5′ ends have an overlap sequence conserved for each part category. Overlapping sequences between genetic parts were designed for optimal primer annealing temperatures and to give consistently high yields after amplification. After this initial cloning, only four primer pairs are required to assemble any variation of the plasmid (protocol detailed in Supplementary Note 2). The combinatorial assembly is very efficient in our hands, and we routinely assembled four-pieces using the New England Biolabs (NEB, Ipswich, MA) HiFi assembly mix (Supplementary Notes 1–2). In most cases, we were also able to amplify three parts as a single product and efficiently create a library of variants with the fourth piece. Two- and three-piece assemblies can also be efficiently performed using NEB HiFi assembly or CPEC cloning (29).

The toolbox includes several variants of each part to facilitate identifying a part that may function in any member of the Proteobacteria. The available parts for assembly include four origins of replication or a Tn7 integration vector as the backbone, 12 promoter–regulator pairs, eight antibiotic markers and seven reporters (detailed in Supplementary Figures S1–S4, Supplementary Table S1). Of the 12 regulators, seven were taken from the Marionette strain of *E. coli*, where these parts were engineered for increased orthogonality in *E. coli*. These include quorum sensing systems regulated by CinR^AM^ and LuxR, cumate-, salicylic acid- and naringenin-inducible systems derived from *P. putida*, a vanillate-inducible system from *C. crescentus*, and a PcaU-regulated system from *Acinetobacter* sp. ADP1 (2) (Supplementary Tables S2 and S5). The remaining promoter-regulator pairs incorporate TetR-regulated systems and sugar-inducible systems regulated by LacI, AraC and RhaS–RhaR from *E. coli*.

In most cases, the RiboJ ribozyme site was included downstream of the promoter to decrease context-dependence issues that may affect gene expression. The ribozyme site self-cleaves in the 5′ UTR, so that the same sequence is present regardless of the promoter used (30). The promoter-regulator part also has a strong RBS positioned upstream of the reporter or GOI in the final assembly for seamless insertion of the coding sequence. All four origins of replication in our toolbox are known to be broad-host-range, though not all origins are efficiently transformed or maintained in all Proteobacteria (31,32).

### Plasmid screens across the Proteobacteria

To demonstrate that our plasmids can identify functional and inducible promoters across the Proteobacteria, we tested each of the organisms in Table 1, representing the three major classes of Proteobacteria. We first assessed the transformation efficiency by electroporation of all four origins in our selected bacteria (Table 1) and found that the efficiency varied significantly (Supplementary Figure S5 and Supplementary Table S4). In some bacteria, all plasmids were transformed with high efficiency, others had large differences in efficiency between each origin of replication, and some origins could not be transformed into a particular host. Each origin part also possesses an origin of transfer (OriT) that allows conjugation with RP4 conjugal machinery for transfer to bacteria that cannot be efficiently electroporated. We confirmed that conjugation was possible using triparental mating with the mobilizable pEVS104 helper plasmid to conjugate *A. fischeri* (33).

**Table 1.** Strains investigated in this study

Strain	Phylogenetic class
*Acinetobacter baylyi* ADP1	Gammaproteobacteria
*Agrobacterium fabrum* C58	Alphaproteobacteria
*Burkholderia thailandensis* E264	Betaproteobacteria
*Pseudomonas aeruginosa* PAO1	Gammaproteobacteria
*Pseudomonas putida* KT2440	Gammaproteobacteria
*Sulfitobacter* sp. EE-36	Alphaproteobacteria
*Ruegeria* sp. TM1040	Alphaproteobacteria
*Xanthomonas campestris* ATCC 33913	Gammaproteobacteria
*Aliivibrio fischeri* ES114	Gammaproteobacteria

We next collected data from inducible systems expressing different fluorescent proteins as reporters in a few strains (Supplementary Figure S6 and S7). After preliminary screens using the reporters GFPmut3 (34), mKelly2 (35), tdKatushka2 (36) and mRFP (37), we proceeded with mRFP as it consistently gave a larger dynamic range of expression and was less affected by autofluorescence of cells and medium. The other fluorescent proteins are still included in the toolbox as they could be useful for specific circumstances.

**Figure 2.**
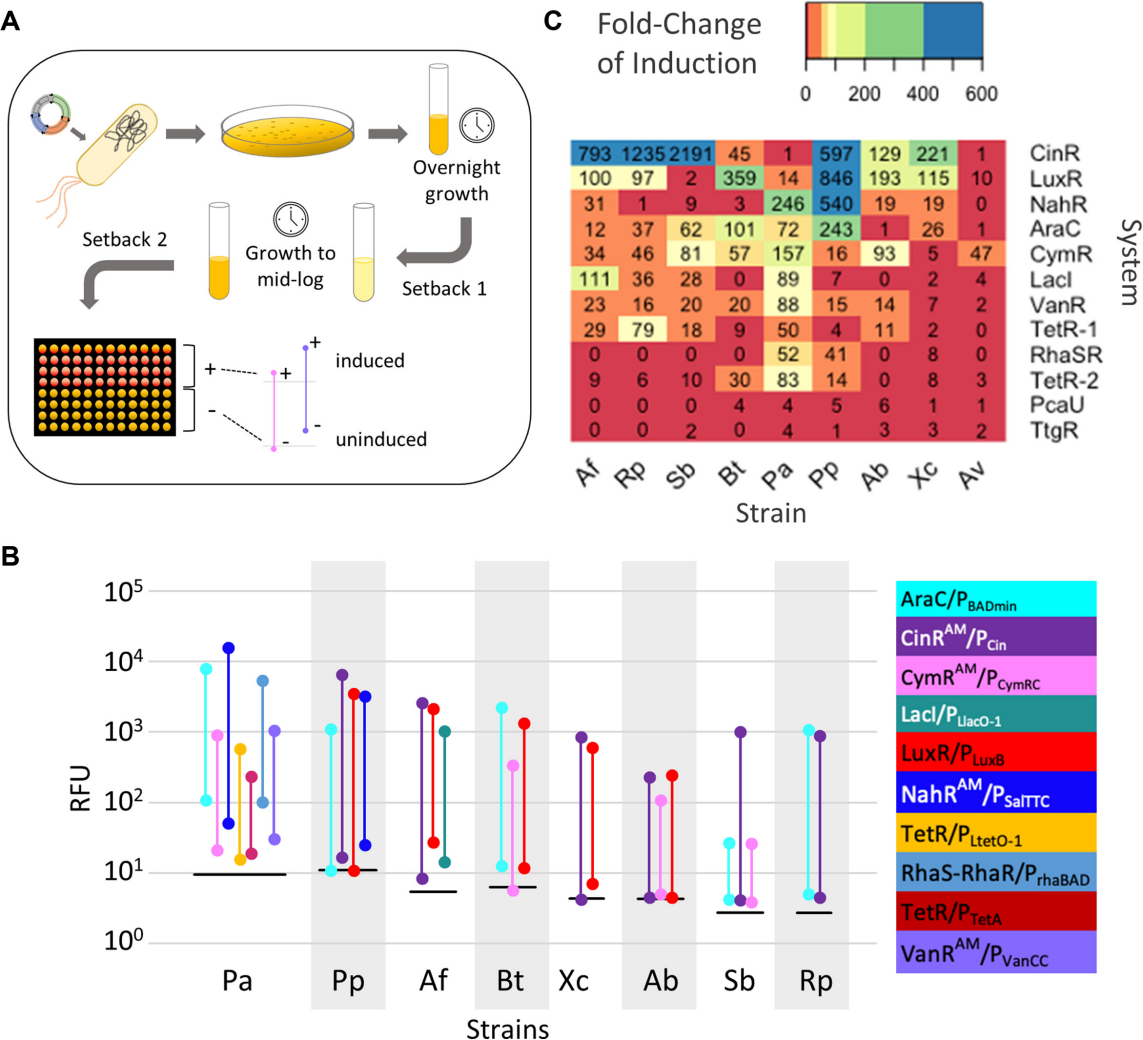
Experimental outline and induction screen results. (**A**) Workflow for inducible systems screens. (**B**) Promoter-regulators with >50-fold induction range. Fold change was calculated without correcting for autofluorescence of the cells and medium. Floating lines represent the induction range of mRFP with fluorescence in the absence of inducer plotted at the bottom of each line and induced expression plotted at the top of the vertical line. Data is clustered by the host strain. Strains on x-axis: Pa (*P. aeruginosa*), Pp (*P. putida*), Af (*A. fabrum*), Bt (*B. thailandensis*), Xc (*X. campestris*), Ab (*A. baylyi*), Sb (*Sulfitobacter* sp. EE-36) and Rp (*Ruegeria* sp. TM1040). Horizontal lines at each cluster represent the average fluorescence of control strains that did not possess *mRFP*. (**C**) Fold Change Heatmap of all Bacteria and Inducible Systems. The fold change was calculated from RFU data normalized to OD and background fluorescence of the medium and empty vector control after 24 h of growth for all bacteria except *A. fischeri*, where the medium only was used for normalization. Strain abbreviations are the same as in (B) plus Av (*A. fischeri*). Inducible systems on the y-axis are labeled with the transcription factor. For TetR systems, TetR-1 refers to TetR/P_TetA_ and TetR-2 refers to TetR/P_Ltet-O1_.

**Figure 3.**
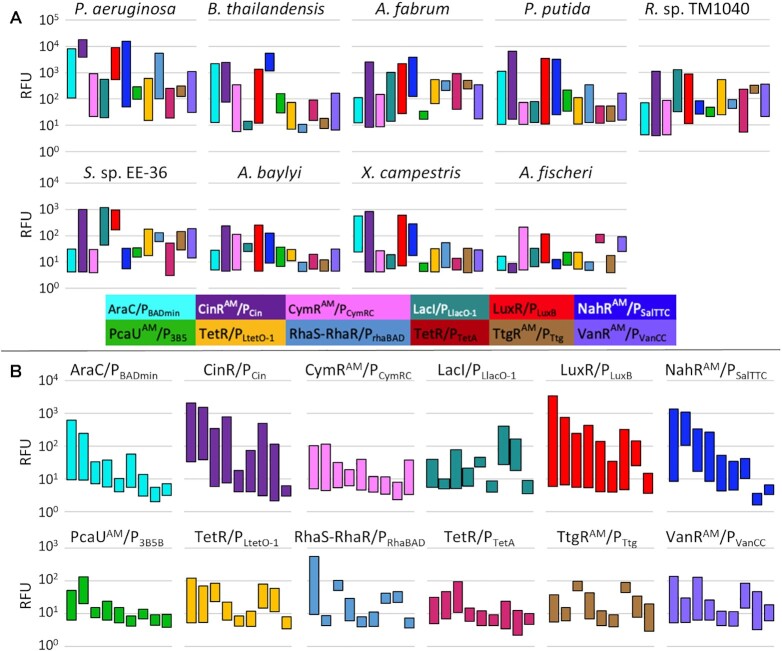
Induction range of 12 expression systems in nine Proteobacteria. (**A**) Expression of mRFP for each of 12 inducible expression systems after overnight growth in each bacterium with induction range represented by floating bars with fluorescence in the absence of inducer plotted at the bottom of each bar and induced expression plotted at the top. (**B**) Expression of mRFP in late-exponential phase of growth graphed by the inducible system. On each graph, expression from the nine Proteobacteria are displayed in the following order: *P. aeruginosa*, *B. thailandensis*, *A. fabrum*, *P. putida*, *A. baylyi*, *X. campestris*, *Ruegeria* sp. TM1040, *Sulfitobacter* sp. EE-36, and *A. fischeri*. Data is presented without correcting for autofluorescence of the cells and medium.

Plasmids were then constructed with each of the 12 promoter-regulator pairs, the mRFP reporter, the gentamicin resistance marker, and either the pK, pB or pF origin. We then transformed the full set of 12 plasmids from the most efficiently electroporated or conjugated origin of replication for each bacterium and screened all inducible systems within each strain in parallel. At least five inducer concentrations were included in the screening plates as an exploratory approach to determine the inducer concentration that gave the highest expression without appreciably compromising growth. The cells were induced during early-log phase, followed by optical density and fluorescence measurements at mid-log and stationary phases of growth. The promoters that had at least 50-fold difference between the uninduced and induced cultures at the 24 h timepoint are shown in Figure 2B, and induction results for all 12 inducible systems in the nine Proteobaceteria are shown in Figure 3 (all data shown in Supplementary Figures S8–S16). Because some of the uninduced cultures possessed slightly lower fluorescence than a control without mRFP, the background fluorescence was not subtracted, and the data is presented as raw values.

In eight bacteria, at least two promoter-regulators were found to have an induction range of over 50-fold. Surprisingly, commonly used systems derived from *E. coli*, such as LacI- and TetR-regulated promoters, were not among those with the largest expression ranges in our dataset. The CinR^AM^/P_Cin_ and LuxR/P_LuxB_ systems were generally the best performing across all of the bacteria we screened, with the CinR^AM^-regulated system achieving over 120-fold induction in all but three of the nine strains (Figure 2C). While eight of the nine strains possess at least one native quorum sensing system induced by a homoserine-lactone, the functionality of these heterologous systems does not appear related to endogenous quorum sensing capabilities. The one strain that does not possess related quorum sensing genes, *X. campestris*, is still inducible by over 100-fold (38). Nonetheless, our data shows these regulators are highly sensitive in the majority of the strains tested, with concentrations less than 10 μM sufficient for full induction. NahR^AM^/P_SalTTC_ was consistently among those with the highest induction, though very leaky by 24 h in some strains. Conversely, CymR^AM^/P_CymRC_ was moderately inducible but remained tight in the absence of inducer in virtually all strains. Though not as highly inducible as the quorum sensing-derived systems, VanR^AM^/P_VanCC_ and AraC/P_BADmin_ were functional across all strains tested with varying levels of leakiness. TtgR^AM^/P_Ttg_ and PcaU^AM^/P_3B5B_ were most likely to be non-functional and many times there was little to no difference in output between on and off states (Figures 2C and 3).

The induction profiles of Alphaproteobacteria *A. fabrum*, *Ruegeria* sp. TM1040, and *Sulfitobacter* sp. EE-36 share some similarities (Figure 3, Supplementary Figure S8, S14 and S15). The cumate-, arabinose-, and OHC14-inducible systems are similarly tightly off in the absence of inducer with CymR^AM^/P_CymRC_ and AraC/P_BADmin_ inducible to nearly the same degree across all three strains. The expression profiles of TetR/P_LtetO-1_ are also very similar across timepoints, with leakiness apparent at 4 h post-induction and moderate though leaky expression after an overnight of growth. LacI/P_LlacO-1_ is leaky but still highly inducible in both roseobacter species and in all three Alphaproteobacteria, the RhaS-RhaR- and PcaU^AM^-regulated systems were leaky and not inducible.

Behavior across promoter-regulator pairs in the two *Pseudomonas* species was surprisingly different given their close phylogenetic relationship. The LuxR- and CinR^AM^-regulated systems were inducible to 846- and 597-fold respectively in *P. putida*, but only 14- and 2-fold in *P. aeruginosa*, due to high leakiness (Figures 2C and 3, Supplementary Figure S11). There was also more than a 150-fold difference between the induction levels of AraC/P_BADmin_, though both reached similar levels of induction. Conversely, while CymR^AM^-, TetR-, VanR^AM^- and LacI-regulated systems were inducible over 50-fold in *P. aeruginosa*, the same systems had a less than 20-fold change in *P. putida*. The shared induction trends in the two roseobacter species and stark differences between *Pseudomonas* species highlight the lack of predictability of tools for controlling gene expression in closely related bacteria.

**Figure 4.**
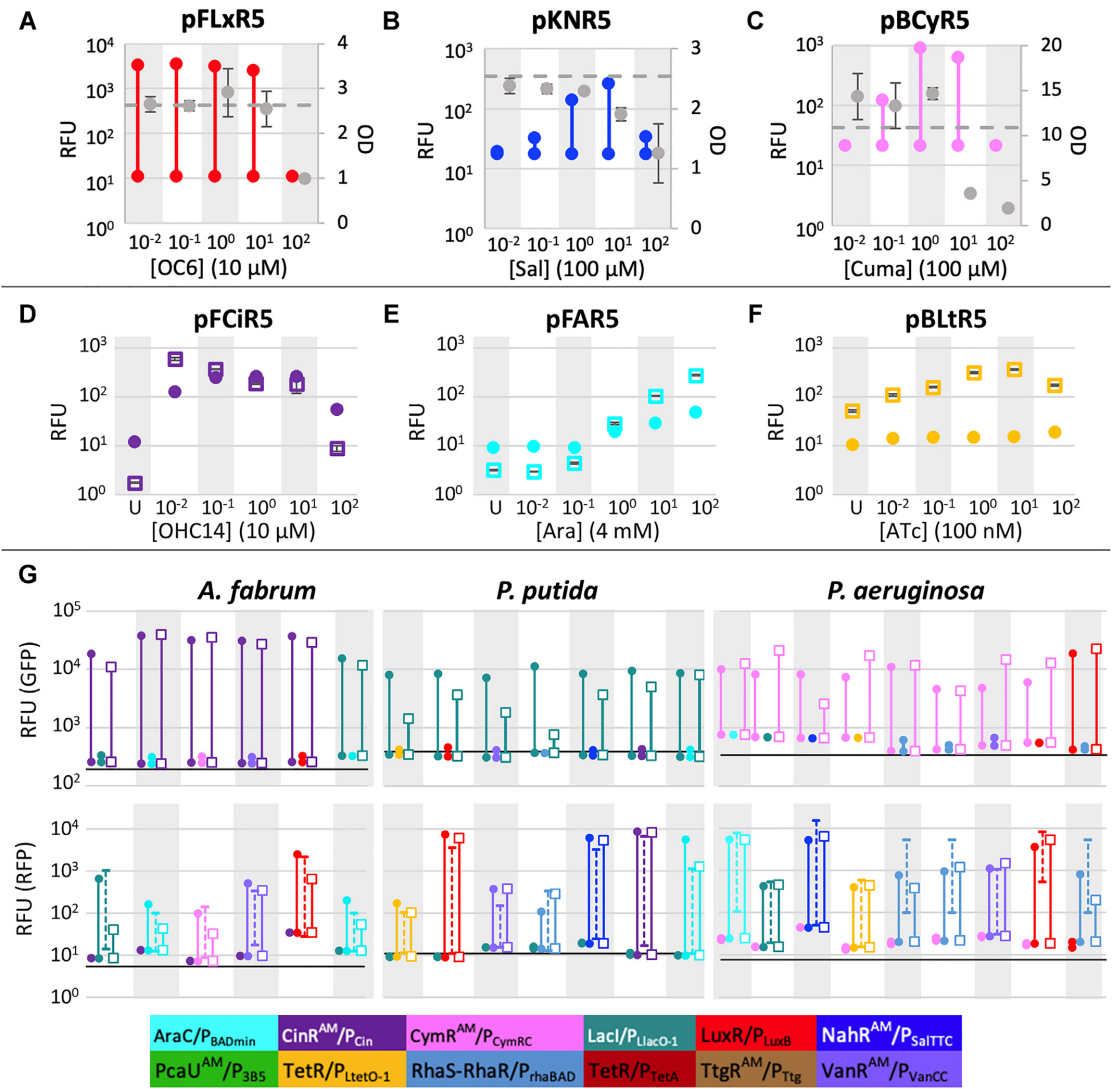
Measurement of mRFP at Titrated Inducer Concentrations. (**A**) pFLxR5 in *P. putida* (**B**) pKNR5 in *X. campestris* (**C**) pBCyR5 in *P. aeruginosa*. Vertical bars represent range of expression at five concentrations of inducer in RFU and gray circles are OD_660_ at late stationary phase ± SE of triplicates. (**D**) pFCiR5 in *A. fabrum* (**E**) pFAR5 in *B. thailandensis* (**F**) pBLtR5 in *Ruegeria* sp. TM1040. Data points represent fluorescence normalized to growth (OD_660_) from samples grown in the absence of inducer (U) and at five inducer concentrations. Exponential phase (closed circles) and late stationary phase (open squares) ± SE of triplicates. (**G**) Expression data from independent induction experiments. Strains containing two plasmids with unique promoter-regulator pairs and reporters were induced both individually and simultaneously. For each bacterium, the top and bottom graphs show fluorescence data from GFP and mRFP, respectively. Plasmid combinations are listed in Supplementary Table S6. For each data cluster, floating lines represent expression from the following conditions in order: expression with inducer for GFP (closed circle), expression with inducer for mRFP (closed circle), and expression with both inducers (open square). Data from strains with the corresponding single plasmid are included on mRFP graphs (dashed line). The data shown are the average RFU of triplicates after an overnight induction.

The inducer concentration that yielded the highest expression level was not consistent between the different bacteria tested, likely a consequence of different uptake capabilities (18,39) (Figure 4A-F, Supplementary Notes 3, 4). In some cases, there was little difference in expression across multiple inducer concentrations, and in a few cultures the highest level of inducer exhibited toxicity that prevented growth of the culture (Figure 4A). Alternatively, some inducible systems gave expression ranges in the shape of a bell curve across titrated inducer concentrations (Figure 4B-C). In other cases, a saturation of fluorescent protein expression was clearly reached at a concentration less than the maximum (Figure 4D). Consistent with promoter responses observed previously, most of our data showed a limited degree of tunability with varying inducer concentration (40) (Figure 4A–F, Supplementary Notes 3, 4). The quorum sensing-derived systems regulated by CinR^AM^ and LuxR frequently exhibited a near-binary response across the inducer concentrations tested, especially after 24 h of induction, though further titrations of these inducers might show a more linear response. Arabinose-, cumate-, vanillate-, and ATc-inducible systems were more likely to show some tunability across at least three inducer concentrations, indicating a space where finely tuned dose-dependent responses could be explored.

### Assessment of context-dependence

Each genetic part in our library should function independently of the other parts in the plasmid. However, in practice, the issue of context-dependence frequently arises, where changing a single gene or genetic part affects nearby parts (41). To demonstrate that plasmid performance was not heavily influenced by context-dependence, we first characterized the effect of each origin of replication on the behavior of the promoter-regulator. To do so, we compared four plasmids that were identical except for the origin of replication in both *P. putida* and *P. aeruginosa*. The uninduced expression was similar across the four different origins in both bacteria (Supplementary Figure S17). The LuxR/P_LuxB_ promoter-regulator pair in *P. putida* performed similarly on a pBBR, RSF1010, and pSa backbone with a fold change difference of less than 10 at two of the three inducer concentrations tested. The same system on an RK2 backbone had a noticeably smaller range of expression, likely due to the lower copy number of RK2 in *P. putida* as compared to other broad-host-range origins (18). This gives an example of how the choice of plasmid origin can be used to add an additional level of control over gene expression (42). VanR^AM^/P_VanCC_ in *P. aeruginosa* also exhibited a smaller dynamic range on an RK2 backbone. The Tn7 integration plasmid was tested in *P. aeruginosa* and *A. fabrum* with the VanR^AM^/P_VanCC_ system. Both bacteria had a decreased leakiness and a lower range of expression compared to the same regulator on a replicating plasmid, as expected because of single copy expression from the chromosome (Supplementary Figure S18). These results demonstrate that promoter-regulator integration provides an alternative to vector-based expression to tune gene expression to the levels needed.

We also assessed whether changing the resistance marker on otherwise identical plasmids affected promoter induction in *A. fabrum*. mRFP expression was measured from four promoter-regulator pairs, VanR^AM^/P_VanCC_, CinR^AM^/P_Cin_, CymR^AM^/P_CymRC_ and LuxR/P_LuxB_ on kanamycin and gentamicin backbones. The results show that the relative expression for each system remained the same though there were slight differences in the absolute level of expression (Supplementary Figure S19). Comparing induction of the same system on each marker within each timepoint, only one of the four promoter-regulator pairs had a difference of >2-fold in basal expression and one had a difference of higher than 3-fold in induced expression. Relative to the range of expression of these systems, these differences are quite small. In sum, these results demonstrate that there is context-dependence in gene expression, but it is minimal when comparing most plasmids. Moreover, the differing levels of expression could be strategically used to optimize expression needed for specific usage.

To test whether these systems were orthogonal to each other and thus allow independent induction in the same cell, two plasmids with different inducible systems were transformed into *P. putida*, *A. fabrum*, and *P. aeruginosa* (plasmid combinations listed in Supplementary Table S6). The systems were induced both individually and simultaneously to assess cross-reactivity and metabolic burden. In the absence of its cognate inducer, there was little expression from any of the regulators from early log-phase through late exponential (data from overnight induction shown in Figure 4G). In *A. fabrum* and *P. putida*, strains with both LacI- and AraC-regulated systems were tested in combination because IPTG is known to inhibit expression from the AraC-regulated promoter (43). We expected that the versions used here would be more compatible because much of the native regulatory sequence was removed from the promoter regions. Induction of AraC/P_BADmin_ in the presence of IPTG was lower than when the two-plasmid strains were induced with only arabinose, though the effect was less in *P. putida* than in *A. fabrum* and the AraC-regulated systems were still functional in both cases. Induction rank order trends in the two-plasmid systems generally followed those from individual plasmid experiments (plotted on Figure 4G with dotted lines, data from Figure 3, Supplementary Figures S8, S9 and S11). However, in most cases the expression from single-inducer induction did not achieve the same maximum levels as in the individual plasmid experiments, likely due to stress from the maintenance of two plasmids and their corresponding antibiotics. Expression was generally lower when both systems were induced, as expected, though the difference in dynamic range between single and simultaneous induction varied considerably depending on the two systems involved. For example, induction of pBLlG2 with IPTG in the presence of rhamnose dramatically decreased expression of GFP in *P. putida*, while the induction of LacI/P_LlacO-1_ was virtually unchanged when arabinose was also present. Nevertheless, these results confirm that our plasmids can be used to independently control the expression of multiple genes, though optimization of experimental design may be necessary to attain desired expression levels in any given host.

### Controlled expression of a physiologically relevant gene

While the fluorescent protein assays described above are useful for identifying the fold change in expression, they are not ideal for determining the tightness of the promoter because low levels of fluorescent protein expression can be obscured by autofluorescence of the cells and medium (44,45).

**Figure 5.**
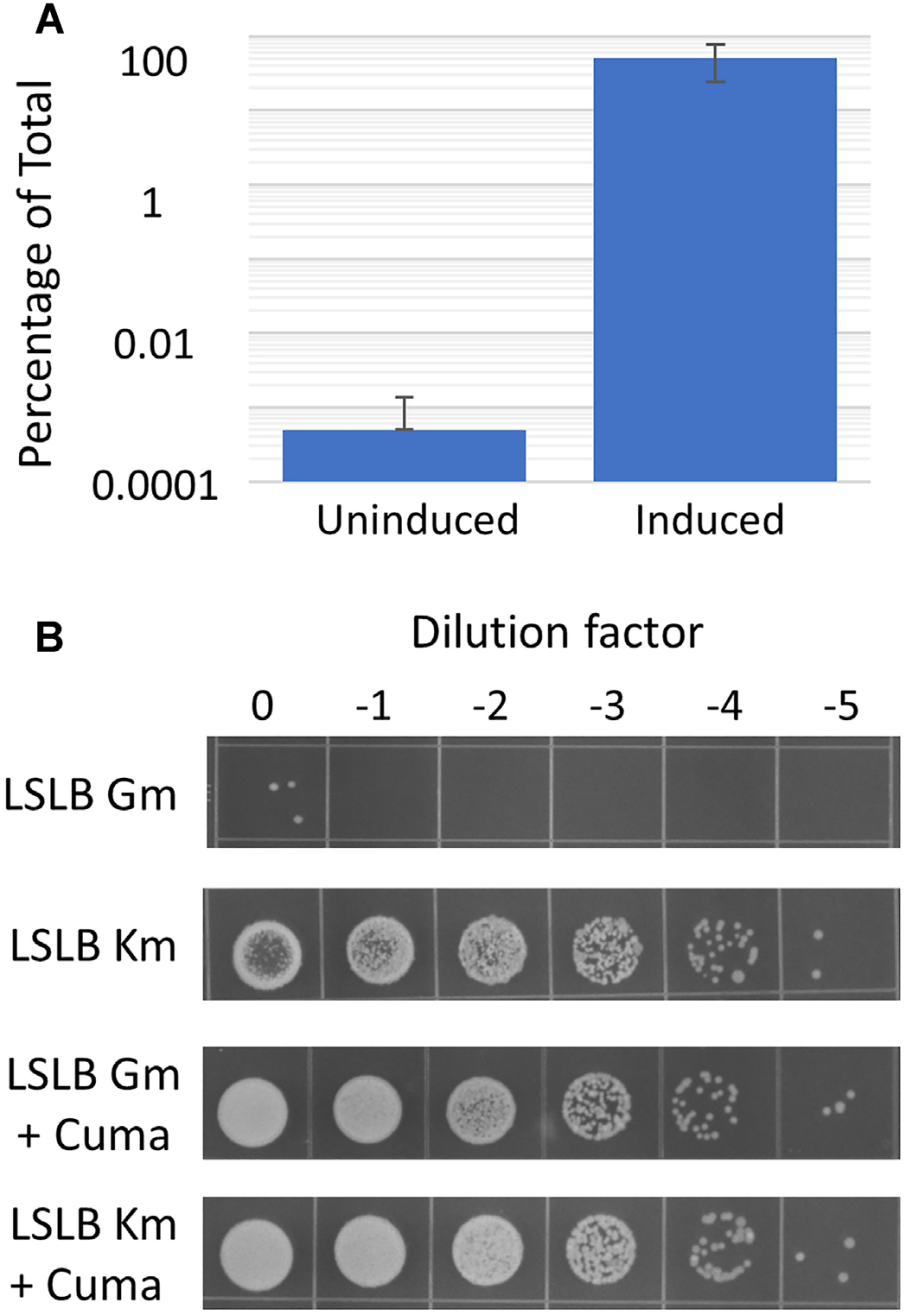
Conditionally essential gene to measure tightness of repression. The gentamicin acetyltransferase gene *aacC1* is placed under control of P_CymRC_ in non-inducing and inducing conditions. (**A**) *B. thailandensis* pKCyGe2 strains are plotted as a percentage of the total number of viable cells containing the plasmid. (**B**) Serial dilutions of *B. thailandensis* plated onto media containing gentamicin or the backbone antibiotic kanamycin with and without the addition of the inducer cumate. Data from cultures in exponential phase of growth.

To examine leaky expression with a physiologically relevant gene, we cloned the gentamicin resistance gene *aacC1* into a plasmid with the CymR^AM^/P_CymRC_ system to create plasmid pKCyGe2. Based on our fluorescence data, this system remained tightly repressed through log phase in *B. thailandensis* (Supplementary Figure S10) and should yield cells that are sensitive to gentamicin in the absence of inducer. After reaching mid-log phase the strains were serially diluted and spotted on agar plates (Figure 5). Both induced and uninduced cultures were spotted onto gentamicin plates, to quantify expression of *aacC1*, and kanamycin plates, to count the total number of viable cells. The results showed nearly a 200 000-fold change difference between uninduced and induced colony counts when each value is calculated as a proportion of total viable cells, demonstrating that this assay was very sensitive and there was a quick response to induction. In the absence of inducer only 5 × 10^–4^ cells/ml were viable, demonstrating that the promoter-regulators identified in the mRFP screen did in fact possess very low levels of leaky transcription (Supplementary Figure S20).

### Promoter libraries enable varied dynamic range

**Figure 6.**
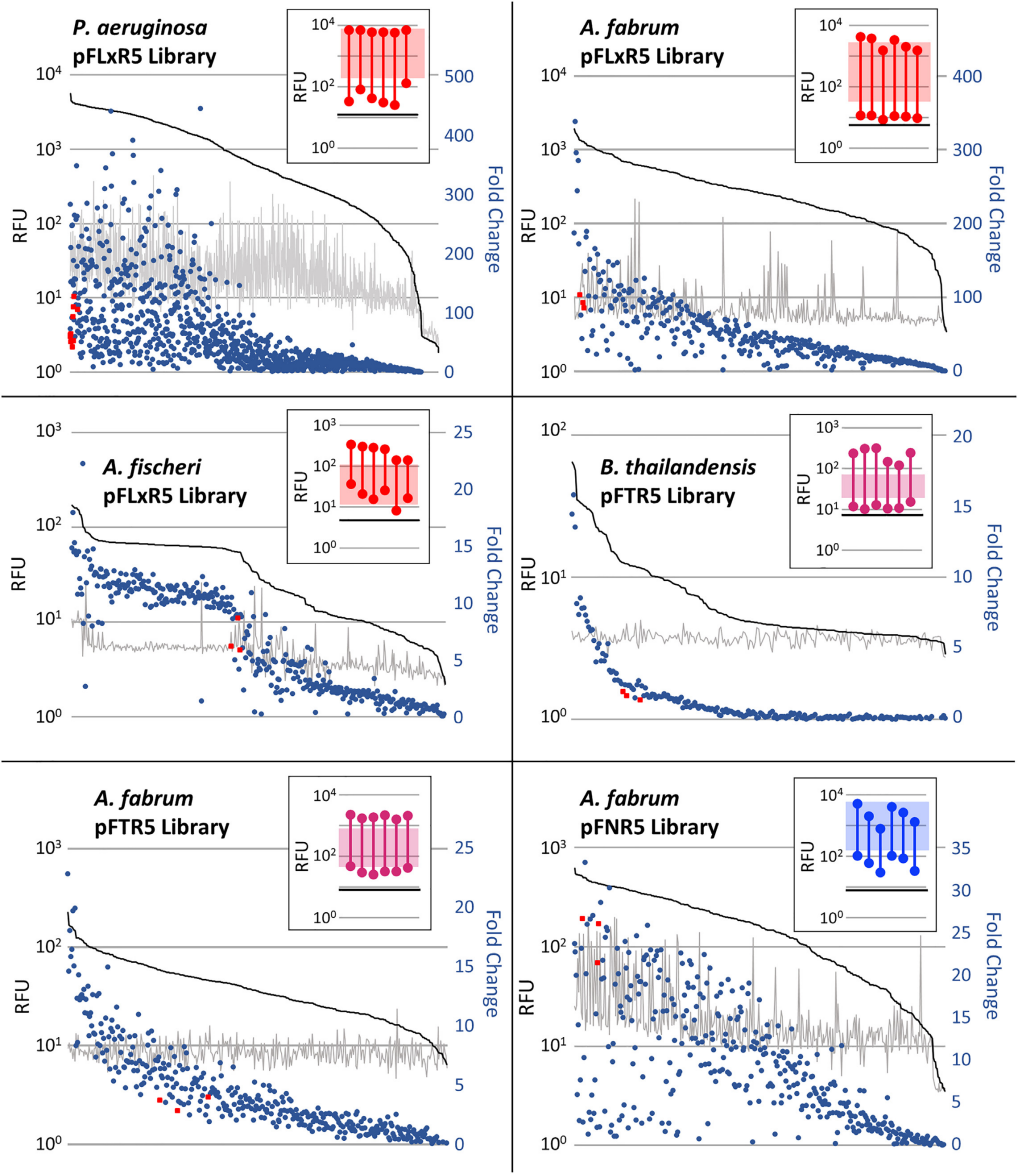
Expression of Total Library and Select Library Isolates. Expression data from all screened isolates of the LuxR/P_LuxB_ library in *P. aeruginosa* (top left), *A. fabrum* (top right), and *A. fischeri* (center left), the TetR/P_TetA_ library in *B. thailandensis* (center right) and *A. fabrum* (bottom left), and the NahR^AM^/P_SalTTC_ library in *A. fabrum* (bottom right). Grey and black lines show uninduced and induced expression of each isolate, respectively, and the overlayed scatterplot shows corresponding fold change. Symbols in red represent the fold change of original plasmids. Data is sorted by induced RFU. Inserted floating bar charts represent expression ranges from isolates with the highest fold change from each respective library. Expression range from original plasmid represented in a shaded box, fluorescence from empty vector control shown as a black horizontal line. Data is an average of three replicates after overnight induction.

Often, experiments that use inducible expression systems require a specific range of expression or very tight repression (46–49). Accordingly, we sought to construct plasmids that had an array of dynamic ranges with a single inducible system, such that the user can easily choose the one that best fits their needs. There are a few available methods in the literature to change dynamic range, including modification of promoter architecture or mutating transcription factors themselves (50–52). Because our inducible systems are diverse (i.e., utilizing activators or repressors as transcription factors) and each system-host pairing is unique, we chose an exploratory approach through degenerate promoter libraries. Libraries were built in the LuxR/P_LuxB_, NahR^AM^/P_SalTTC_ and TetR/P_TetA_ systems by targeting both the promoter driving expression of the reporter and the promoter of the regulator (Supplementary Note 5) since the concentration of the regulatory protein has a direct influence on GOI output (15). We then examined these libraries in *B. thailandensis, P. aeruginosa, A. fabrum*. or *A. fischeri* by first screening 200–1100 variants to identify those that possessed decreased leakiness or a larger dynamic range of expression than the original after 24 h (Figure 6).

Between 20–40 variants were then re-screened in a more sensitive assay to calculate the induction range, a subset of which are shown in the chart inserts in Figure 6 (expression data from all re-screened isolates shown in Supplementary Figure S21). In *A. fabrum*, libraries of plasmids pFNR5 and pFTR5 had variants with induction fold changes of 54 and 108, compared to 40 and 26 for the original plasmids. For the pFLxR5 library, the fold change increased from 120 to 722, with the tightest variants nearing the detection limit for mRFP in our assay. The original pFTR5 construct performed poorly in *B. thailandensis* with a fold change of only 3, while the best members of the TetR/P_TetA_ library had a fold change of 105, with increased expression in the on-state and decreased leakiness in the off-state. In *A. fischeri*, the pFLxR5 library had variants with an increased on-state but none that had a decreased leakiness. Lastly, in *P. aeruginosa* the original pFLxR5 plasmid was very leaky at the 24 h time point, whereas the selected variants were much tighter in the absence of inducer at 24 h, increasing the fold change from 29 to 445 (Figure 6, Supplementary Figure S21).

Library isolates with an increased dynamic range were either more tightly off in the absence of inducer, more highly expressing when induced, or both. The improved isolates from the LuxR-regulated library in *P. aeruginosa* and the LuxR- and NahR^AM^-regulated libraries in *A. fabrum* were less leaky than the original systems after an overnight induction but were similar in the on-state compared to the original. Expression data from the complete library and re-screened selected isolates suggests that the original inducible system was already expressing near the physiological limit for that strain (Figure 6, Supplementary Figure S21). Induction results from single-plasmid screens support this as the LuxR/P_LuxB_ system is among the highest expressing of the 12 systems screened in *P. aeruginosa* and *A. fabrum*, and NahR^AM^/P_SalTTC_ is the highest overall expressing system in *A. fabrum* (Figure 3). Conversely, the improved isolates from libraries of the LuxR-regulated system in *A. fischeri* and the TetR/P_TetA_ system in *B. thailandensis* had markedly higher induced expression compared to the original systems. Though the LuxR used here is from *A. fischeri*, the original plasmid possesses a mutation in the -10 hexamer of the regulated promoter that was made to improve dynamic range and decrease cross-reactivity in *E. coli* (2), and ostensibly had the effect of decreasing induction in its native host.

In a few libraries, isolates with the highest fold change were so tightly off in the absence of inducer that they neared the detection limit of our fluorescence assay. To further confirm that these LuxR/P_LuxB_ promoters in *P. aeruginosa* were tightly repressed, we cloned the five gene violacein biosynthesis pathway from *Pseudoalteromonas luteoviolacea* with its native operon structure in place of *mRFP* in the original pFLxR5 plasmid and several members of the LuxR/P_LuxB_ library (53). Despite the large size of this operon (7.4 kb), four-piece plasmid assemblies were efficient. Without inducer, the amount of violacein extracted from each strain with a variant promoter was near the limit of detection because the crude extract measurements were similar to the negative control, while the strain with the original promoter was nearly 6-fold greater (Supplementary Note 6). In total, these results demonstrate that the dynamic range can be changed by screening a modest number of variants with targeted degeneracies and that promoter-regulators that appear non-functional can sometimes be improved to produce a robust dynamic response, as was the case with the TetR/P_TetA_ system in *B. thailandensis*. Moreover, these libraries can be directly used to screen for activity with desired properties in bacteria where few or no such systems exist.

## DISCUSSION

The comprehensive screening of inducible systems described in this work demonstrates the utility of a standard vector assembly method for identifying and characterizing gene expression systems in diverse bacteria. The plasmid-based design of these systems ensures modularity and facilitates quick and easy movement into and between hosts for testing with a fluorescent protein, with the option to integrate the system into the chromosome if desired. The inherent design scheme of the system requires that all four genetic parts are present and in the pre-determined order in the plasmid, and deviating from the design requires creating new primers (details in Supplementary Note 1). Nevertheless, moving from testing to utilization is quick since there are no sequence-based restrictions for adding a gene of interest in place of the reporter.

The bacteria tested in this work include species that are well-studied and widely used, such as *Pseudomonas*. sp*, A. fabrum*, and *B. thailandensis*, as well as those lesser studied, i.e. *Ruegeria* sp. TM1040 and *Sulfitobacter* sp. EE-36. Similarly, the inducible systems screened herein include the commonly used rhamnose, arabinose, and IPTG-inducible systems (54–56), while the NahR, LuxR, and CinR regulators are arguably under-utilized given their effectiveness for controlling gene expression in several of the bacteria that we tested. When comparing the data gathered here to published expression data from systems in the same bacteria, our plasmids provide either a larger range of inducibility or a lesser degree of leakiness in many cases. Further, these comparisons demonstrate a lack of predictability for expression systems moved from host to host, highlighting the need for standardization and broadly available genetic tools.

*P. putida* has emerged as a prominent microbial chassis for metabolic engineering due to its versatile metabolism and stress-endurance traits (57). Both native and heterologous inducible systems have been employed to control gene expression with varying success. Screening natural *E. coli* inducible promoters P_RhaB_, P_AraB_, P_LacUV5_ and P_T7_ and *Psuedomonas* promoters P_m_, P_Sal_, and P_AlkB_ in *P. putida*, all but P_AraB_ had leakiness that was at least two orders of magnitude above background, with the P_m_ promoter being the leakiest (39). In comparison, our data shows that basal expression was near baseline for the majority of systems screened while still inducible by up to 850-fold (Figure 2C). Differences in expression observed when comparing *E. coli* to *P. putida* emphasize the unpredictability that comes with moving a system into a new host (39,58–60).

Increasingly important synthetic biology hosts include *A. fabrum* and *Burkholderia* sp., due to their relevance as etiological agents of disease (61–63), and *A. baylyi* (64), due to its genetic malleability. The most commonly used systems for regulating gene expression in these bacteria are the *E. coli* IPTG-, arabinose- and rhamnose-induced systems. Previous work in *A. fabrum* had mixed results on LacI-regulated systems' effectiveness, with LacI^q^/P_Lac_ exhibiting only a 6-fold change in expression when induced (32) while LacI/P_Lac_ was induced over 300-fold (40). AraC-regulated systems are highly inducible in *A. fabrum* with low basal expression (31,40). Our data demonstrate that LuxR- and CinR-regulated systems are significant additions to these available tools, with expression levels at 100- and near 800-fold, respectively.

Arabinose- and rhamnose-inducible system are the most widely used for controlling gene expression in *Burkholderia* sp. (31,65,66), inducing 5- to 21-fold higher than *E. coli* (67). In *B. thailandensis*, we observed inductions of over 350-fold with LuxR/P_LuxB_ and over 50-fold with a cumate-inducible system. In *A. baylyi*, the *E. coli* promoters P_BAD_ and P_Tac_ were inducible to over 100-fold with varying levels of basal expression (4). Similarly, another group found the IPTG-inducible Trc, Tac, and T5 promoters to be highly inducible in *A. baylyi* ADP1 and generated a Trc promoter library, identifying isolates with up to a 73-fold induction (26). While the *E. coli*-derived systems that we tested did not have a high range of induction, we observed fold changes of 100 to 200 from CinR^AM–^, LuxR-, and CymR^AM^-regulated systems in *A. baylyi*, thus expanding the options for gene regulation in this organism. The degenerate library methodology employed here successfully expanded the expression range and presented a simple method to identify promoters with expression in the desired range. Perhaps the most surprising observation in our data was that the largest dynamic range of expression came from the CinR^AM^-regulated system in *Ruegeria* sp. TM1040 and *Sulfitobacter* sp. EE-36, 1235-fold and 2191-fold respectively. These results confirm that our toolbox can identify systems for gene regulation where none existed before.

The toolbox described here allows for the systematic evaluation of the three key components needed to develop genetics systems in non-model bacteria: the origin of replication, antibiotic resistance marker, and promoter-regulator. Designed for inducible gene expression, these plasmids are not as customizable as other toolboxes, but their simplicity and ease-of-use expedites the design and build stages of the bio-engineering design-build-test-learn cycle. Even in bacteria that have developed genetics systems, these plasmids will enable parts standardization, increased reproducibility, and streamlined cloning to speed plasmid construction. As microbial synthetic biology continues to move into more diverse hosts, predictable broad-host-range expression systems will be essential to advance the field. These systems can be used for various applications, such as directing flux toward value-added products in metabolic engineering (49) and implementing heterologous tools for genetic manipulation. For example, tools such as CRISPR-interference or CRISPR-activation have enhanced our capacity to manipulate bacterial cells, but they still require control at the transcription level for precise temporal function (68–70). These systems are only as good as the underlying promoters driving the expression of the Cas genes. In most bacteria, there are no well-developed options, making this toolbox of immediate practical value. Though we only tested members of the Proteobacteria, the same genetic parts will likely function in other Gram-negatives, and perhaps an even wider range of bacteria since there is a precedence that the RSF1010 origin of replication can be maintained in some Gram-positive bacteria (71). There is evidence that some of the inducible systems tested here are functional in Gram-positive species as well. For example, the Tn*10* encoded *tet* regulatory system has been used in *Bacillus subtilis* (72), and the AraC-regulated system functions in *Corynebacterium glutamicum* (73). Importantly, the orthogonality of most of the promoter-regulators and the availability of both multiple origins of replication and antibiotic markers will facilitate experiments that require independent expression of multiple genes in the same cell. With genetic part versatility, flexible swapping, and ease of new part addition, this toolbox is a valuable addition to the field and will be useful as new microbial hosts are explored.

## DATA AVAILABILITY

A selection of plasmids that include each of the genetic parts described here are available from Addgene. A complete list of plasmids and Addgene numbers can be found in Supplementary Table S1.

## Supplementary Material

gkab496_Supplemental_File
