# Supplementary material for: A plasmid toolbox for controlled gene expression across the Proteobacteria
Source: Nucleic Acids Res. 2021 Jun 14;49(12):7189–202. doi: 10.1093/nar/gkab496 (PMC8266580; doi:10.1093/nar/gkab496)
Supplement: gkab496_Supplemental_File [file gkab496_supplemental_file.pdf]

Supplementary Information: A Plasmid Toolbox for Controlled Gene Expression Across the Proteobacteria

Layla A. Schuster and Christopher R. Reisch*.
Dept. of Microbiology and Cell Science, Institute of Food and Agricultural Sciences, University of Florida, Gainesville, FL, 32603, USA
* To whom correspondence should be addressed: Tel: +1 352 846 0955 Fax: +1 352 846 0950
Email: creisch@ufl.edu

Table of Contents

Supplementary Tables
Supplementary Table 1: Plasmids and Parts 4
Supplementary Table 2: Regulatory Element Sources 13
Supplementary Table 3: Strain Growth Conditions 14
Supplementary Table 4: Electroporation Conditions 15
Supplementary Table 5: Inducers 16
Supplementary Table 6: Independent Expression Experiments 32

Supplementary Figures
Supplementary Figure 1: Origin Parts. 9
Supplementary Figure 2: Inducible promoter-regulator parts. 10
Supplementary Figure 3: Reporter Parts. 11
Supplementary Figure 4: Marker Parts. 12
Supplementary Figure 5: Plasmid Transformation Efficiencies. 17
Supplementary Figure 6: Red and Far-Red Reporter Comparison. 18
Supplementary Figure 7: Green and Red Reporter Comparison. 19
Supplementary Figure 8: 12 Inducible Systems in *A. fabrum* with plasmid pF_R5. 20
Supplementary Figure 9: 12 Inducible Systems in *P. putida* with plasmid pF_R5. 21
Supplementary Figure 10: 12 Inducible Systems in *B. thailandensis* with plasmid pF_R5. 22
Supplementary Figure 11: 12 Inducible Systems in *P. aeruginosa* with plasmid pB_R5. 23
Supplementary Figure 12: 12 Inducible Systems in *A. baylyi* with plasmid pK_R5. 24
Supplementary Figure 13: 12 Inducible Systems in *X. campestris* with plasmid pK_R5. 25
Supplementary Figure 14: 12 Inducible Systems in *Ruegeria* sp. TM1040 with plasmid pB_R5. 26
Supplementary Figure 15: 12 Inducible Systems in *Sulfitobacter* sp. EE-36 with plasmid pB_R5. 27
Supplementary Figure 16: 12 Inducible Systems in *Aliivibrio fischeri* with plasmid pF_R5. 28
Supplementary Figure 17: Comparison of mRFP Expression with Different Origins of Replication. 29
Supplementary Figure 18: Measurement of mRFP from the Tn7 Integration Vector and a replicating plasmid in *P. aeruginosa* and *A. fabrum*. 30
Supplementary Figure 19: Comparison of Expression with Different Antibiotic Markers. 31
Supplementary Figure 20: Controlled Expression of the *aacC1* Conditionally Essential Gene. 33
Supplementary Figure 21: Expression Range of Select Library Isolates. 34

Supplementary Notes
Supplementary Note 1: Comparison between Vector Assembly Standards 35
Supplementary Note 2: Plasmid Assembly Protocol and Cost Analysis 37
Supplementary Note 3: OD in Titrated Inducer Concentrations 39

- *A. fabrum* in Exponential Phase. 40
- *A. fabrum* in Stationary Phase. 41
- *P. putida* in Exponential Phase. 42
- *P. putida* in Stationary Phase. 43
- *X. campestris* in Exponential Phase. 44
- *X. campestris* in Stationary Phase 45
- *A. baylyi* in Exponential Phase. 46
- *A. baylyi* in Stationary Phase. 47
- *P. aeruginosa* in Exponential Phase. 48
- *P. aeruginosa* in Stationary Phase. 49
- *B. thailandensis* in Exponential Phase. 50

B. thailandensis in Stationary Phase. 51
Sulfitobacter sp. EE-36 in Exponential Phase. 52
Sulfitobacter sp. EE-36 in Stationary Phase. 53
Ruegeria sp. TM1040 in Exponential Phase. 54
Ruegeria sp. TM1040 in Stationary Phase. 55
A. fischeri in Exponential Phase. 56
A. fischeri in Stationary Phase. 57

Supplementary Note 4: RFU Output Over Titrated Inducer Concentrations 58

P. putida 59
A. fabrum 60
A. baylyi 61
X. campestris 62
B. thailandensis 63
P. aeruginosa 64
Ruegeria sp. TM1040 65
Sulfitobacter sp. EE-36 66
A. fischeri 67

Supplementary Note 5: Library Construction 68

pFTR5 library assembly 68
pFNR5 library assembly 69
pFLxR5 library assembly 70

Supplementary Note 6: Violacein Pathway Expression Experiments 71

Comparing violacein production to mRFP expression in *P. aeruginosa* 72

Supplementary Table 1: Plasmids and Parts

Transformation Efficiency Assays

Addgene	Code	Origin	Regulator	Reporter	Marker
167504	pBLxR5	pBBR	LuxR	mRFP	Gentamicin
149490	pWLxR5	pSA	LuxR	mRFP	Gentamicin
149482	pFLxR5	RSF1010	LuxR	mRFP	Gentamicin
149465	pKLxR5	RK2	LuxR	mRFP	Gentamicin

Reporter Comparisons

Addgene	Code	Origin	Regulator	Reporter	Marker
NA	pBCiR5	pBBR	$CinR^{AM}$	mRFP	Gentamicin
NA	pBCiK5	pBBR	$CinR^{AM}$	mKelly2	Gentamicin
149479	pFCiR5	RSF1010	$CinR^{AM}$	mRFP	Gentamicin
167514	pFCiK5	RSF1010	$CinR^{AM}$	mKelly2	Gentamicin
167511	pBVR5	pBBR	$VanR^{AM}$	mRFP	Gentamicin
NA	pBVK5	pBBR	$VanR^{AM}$	mKelly2	Gentamicin
149489	pFVR5	RSF1010	$VanR^{AM}$	mRFP	Gentamicin
167522	pFVK5	RSF1010	$VanR^{AM}$	mKelly2	Gentamicin
149482	pFLxR5	RSF1010	LuxR	mRFP	Gentamicin
167520	pFVTd5	RSF1010	$VanR^{AM}$	tdKatushka2	Gentamicin
167519	pFLxTd5	RSF1010	LuxR	tdKatushka2	Gentamicin
NA	pBRR5	pBBR	RhaS-RhaR	mRFP	Gentamicin
NA	pBTR5	pBBR	TetR (Tn10)	mRFP	Gentamicin
NA	pBTtR5	pBBR	$TtgR^{AM}$	mRFP	Gentamicin
NA	pBAR5	pBBR	AraC	mRFP	Gentamicin
NA	pBRG5	pBBR	RhaS-RhaR	GFPmut3	Gentamicin
NA	pBTG5	pBBR	TetR (Tn10)	GFPmut3	Gentamicin
NA	pBTtG5	pBBR	$TtgR^{AM}$	GFPmut3	Gentamicin
NA	pBAG5	pBBR	AraC	GFPmut3	Gentamicin
149484	pFPcR5	RSF1010	$PcaU^{AM}$	mRFP	Gentamicin
167506	pFRR5	RSF1010	RhaS-RhaR	mRFP	Gentamicin
149483	pFNR5	RSF1010	$NahR^{AM}$	mRFP	Gentamicin
167507	pFTR5	RSF1010	TetR (Tn10)	mRFP	Gentamicin
NA	pFPcG5	RSF1010	$PcaU^{AM}$	GFPmut3	Gentamicin
149486	pFRG5	RSF1010	RhaS-RhaR	GFPmut3	Gentamicin
NA	pFNG5	RSF1010	$NahR^{AM}$	GFPmut3	Gentamicin
149487	pFTG5	RSF1010	TetR (Tn10)	GFPmut3	Gentamicin
149467	pKPcR5	RK2	$PcaU^{AM}$	mRFP	Gentamicin
149468	pKRR5	RK2	RhaS-RhaR	mRFP	Gentamicin
149466	pKNR5	RK2	$NahR^{AM}$	mRFP	Gentamicin

NA	pKTR5	RK2	TetR (Tn10)	mRFP	Gentamicin
NA	pKPcG5	RK2	PcaU^{AM}	GFPmut3	Gentamicin
NA	pKRG5	RK2	RhaS-RhaR	GFPmut3	Gentamicin
NA	pKNG5	RK2	NahR^{AM}	GFPmut3	Gentamicin
NA	pKTG5	RK2	TetR (Tn10)	GFPmut3	Gentamicin

Inducible Systems Screens

Addgene	Code	Origin	Regulator	Reporter	Marker
149478	pFAR5	RSF1010	AraC	mRFP	Gentamicin
149479	pFCiR5	RSF1010	CinR^{AM}	mRFP	Gentamicin
149480	pFCyR5	RSF1010	CymR^{AM}	mRFP	Gentamicin
149481	pFLlR5	RSF1010	LacI	mRFP	Gentamicin
149482	pFLxR5	RSF1010	LuxR	mRFP	Gentamicin
149483	pFNR5	RSF1010	NahR^{AM}	mRFP	Gentamicin
149483	pFPcR5	RSF1010	PcaU^{AM}	mRFP	Gentamicin
167505	pFLtR5	RSF1010	TetR (Ltet)	mRFP	Gentamicin
167506	pFRR5	RSF1010	RhaS-RhaR	mRFP	Gentamicin
167507	pFTR5	RSF1010	TetR (Tn10)	mRFP	Gentamicin
167508	pFTtR5	RSF1010	TtgR^{AM}	mRFP	Gentamicin
149489	pFVR5	RSF1010	VanR^{AM}	mRFP	Gentamicin
NA	pBAR5	pBBR	AraC	mRFP	Gentamicin
NA	pBCiR5	pBBR	CinR^{AM}	mRFP	Gentamicin
NA	pBCyR5	pBBR	CymR^{AM}	mRFP	Gentamicin
NA	pBLlR5	pBBR	LacI	mRFP	Gentamicin
167504	pBLxR5	pBBR	LuxR	mRFP	Gentamicin
NA	pBNR5	pBBR	NahR^{AM}	mRFP	Gentamicin
NA	pBPcR5	pBBR	PcaU^{AM}	mRFP	Gentamicin
NA	pBLtR5	pBBR	TetR (Ltet)	mRFP	Gentamicin
NA	pBRR5	pBBR	RhaS-RhaR	mRFP	Gentamicin
NA	pBTR5	pBBR	TetR (Tn10)	mRFP	Gentamicin
NA	pBTtR5	pBBR	TtgR^{AM}	mRFP	Gentamicin
167511	pBVR5	pBBR	VanR^{AM}	mRFP	Gentamicin
149461	pKAR5	RK2	AraC	mRFP	Gentamicin
149462	pKCiR5	RK2	CinR^{AM}	mRFP	Gentamicin
149463	pKCyR5	RK2	CymR^{AM}	mRFP	Gentamicin
149464	pKLlR5	RK2	LacI	mRFP	Gentamicin
NA	pKLxR5	RK2	LuxR	mRFP	Gentamicin
NA	pKNR5	RK2	NahR^{AM}	mRFP	Gentamicin
NA	pKPcR5	RK2	PcaU^{AM}	mRFP	Gentamicin
NA	pKLtR5	RK2	TetR (Ltet)	mRFP	Gentamicin
NA	pKRR5	RK2	RhaS-RhaR	mRFP	Gentamicin

NA	pKTR5	RK2	TetR (Tn10)	mRFP	Gentamicin
NA	pKTtR5	RK2	TtgR^{AM}	mRFP	Gentamicin
149469	pKVR5	RK2	VanR^{AM}	mRFP	Gentamicin

Context Dependency Test: Origins

Addgene	Code	Origin	Regulator	Reporter	Marker
167511	pBVR5	pBBR	VanR^{AM}	mRFP	Gentamicin
149489	pFVR5	RSF1010	VanR^{AM}	mRFP	Gentamicin
149469	pKVR5	RK2	VanR^{AM}	mRFP	Gentamicin
167509	pWVR5	pSA	VanR^{AM}	mRFP	Gentamicin
167504	pBLxR5	pBBR	LuxR	mRFP	Gentamicin
149465	pKLxR5	RK2	LuxR	mRFP	Gentamicin
149482	pFLxR5	RSF1010	LuxR	mRFP	Gentamicin
149490	pWLxR5	pSA	LuxR	mRFP	Gentamicin

Integrated Inducible Systems

Addgene	Code	Origin	Regulator	Reporter	Marker
167510	pIVR5	Tn7 integration	VanR^{AM}	mRFP	Gentamicin
149489	pFVR5	RSF1010	VanR^{AM}	mRFP	Gentamicin
167511	pBVR5	pBBR	VanR^{AM}	mRFP	Gentamicin

Context Dependency Test: Markers

Addgene	Code	Origin	Regulator	Reporter	Marker
149489	pFVR5	RSF1010	VanR^{AM}	mRFP	Gentamicin
NA	pFVR2	RSF1010	VanR^{AM}	mRFP	Gentamicin
149479	pFCiR5	RSF1010	CinR^{AM}	mRFP	Gentamicin
167512	pFCiR2	RSF1010	CinR^{AM}	mRFP	Kanamycin
149480	pFCyR5	RSF1010	CymR^{AM}	mRFP	Gentamicin
167513	pFCyR2	RSF1010	CymR^{AM}	mRFP	Kanamycin
149482	pFLxR5	RSF1010	LuxR	mRFP	Gentamicin
NA	pFLxR2	RSF1010	LuxR	mRFP	Kanamycin

Independent Expression Assay

Addgene	Code	Origin	Regulator	Reporter	Marker
149470	pBCiG2	pBBR	CinR^{AM}	GFPmut3	Kanamycin
149481	pFLlR5	RSF1010	LacI	mRFP	Gentamicin
149478	pFAR5	RSF1010	AraC	mRFP	Gentamicin
149480	pFCyR5	RSF1010	CymR^{AM}	mRFP	Gentamicin
149489	pFVR5	RSF1010	VanR^{AM}	mRFP	Gentamicin
149482	pFLxR5	RSF1010	LuxR	mRFP	Gentamicin
NA	pBLlG2	pBBR	LacI	GFPmut3	Kanamycin
167505	pFLtR5	RSF1010	TetR (Ltet)	mRFP	Gentamicin
167506	pFRR5	RSF1010	RhaS-RhaR	mRFP	Gentamicin
149483	pFNR5	RSF1010	NahR^{AM}	mRFP	Gentamicin

149479	pFCiR5	RSF1010	CinR^{AM}	mRFP	Gentamicin
NA	pKCyG2	RK2	CymR^{AM}	GFPmut3	Kanamycin
NA	pBAR5	pBBR	AraC	mRFP	Gentamicin
NA	pBLlR5	pBBR	LacI	mRFP	Gentamicin
NA	pBNR5	pBBR	NahR^{AM}	mRFP	Gentamicin
NA	pBLtR5	pBBR	TetR (Ltet)	mRFP	Gentamicin
NA	pBRR5	pBBR	RhaS-RhaR	mRFP	Gentamicin
167511	pBVR5	pBBR	VanR^{AM}	mRFP	Gentamicin
167504	pBLxR5	pBBR	LuxR	mRFP	Gentamicin
NA	pWCyG2	pSA	CymR^{AM}	GFPmut3	Kanamycin
NA	pKLxG2	RK2	LuxR	GFPmut3	Kanamycin

Leakiness Testing

Addgene	Code	Origin	Regulator	Reporter	Marker
167515	pFCyGe2	RSF1010	CymR^{AM}	Gentamicin	Kanamycin

Library Template Plasmids

Addgene	Code	Origin	Regulator	Reporter	Marker
149482	pFLxR5	RSF1010	LuxR	mRFP	Gentamicin
167504	pBLxR5	pBBR	LuxR	mRFP	Gentamicin
167514	pFCiK5	RSF1010	CinR^{AM}	mRFP	Gentamicin
167507	pFTR5	RSF1010	TetR (Tn10)	mRFP	Gentamicin
149483	pFNR5	RSF1010	NahR^{AM}	mRFP	Gentamicin

Violacein Screening

Addgene	Code	Origin	Regulator	Reporter	Marker
NA	pFLxVi5	RSF1010	LuxR	Violacein	Gentamicin
167516	pBLxVi5	pBBR	LuxR	Violacein	Gentamicin

Library Screening

Addgene	Code	Origin	Regulator	Reporter	Marker
Processing	pFLxR5 Library	RSF1010	LuxR	mRFP	Gentamicin
Processing	pFTR5 Library	RSF1010	TetR (Tn10)	mRFP	Gentamicin
Processing	pFNR5 Library	RSF1010	NahR^{AM}	mRFP	Gentamicin

Additional Plasmids Available at Addgene

Addgene	Code	Origin	Regulator	Reporter	Marker
149470	pBCiG2	pBBR	CinR^{AM}	GFPmut3	Kanamycin
149501	pBLlCa5	pBBR	LacI	AmpR	Gentamicin
149471	pBLtG2	pBBR	TetR (Ltet)	GFPmut3	Kanamycin
149472	pBLxG2	pBBR	LuxR	GFPmut3	Kanamycin
149473	pBNG2	pBBR	NahR^{AM}	GFPmut3	Kanamycin
149474	pBRG2	pBBR	RhaS-RhaR	GFPmut3	Kanamycin
149475	pBTG2	pBBR	TetR (Tn10)	GFPmut3	Kanamycin
149476	pBTtG2	pBBR	TtgR^{AM}	GFPmut3	Kanamycin

149477	pBVG2	pBBR	$VanR^{AM}$	GFPmut3	Kanamycin
149485	pFLtG5	RSF1010	TetR (Tn10)	GFPmut3	Gentamicin
149496	pFLxR1	RSF1010	LuxR	mRFP	Chloramphenicol
149488	pFTtG5	RSF1010	$TtgR^{AM}$	GFPmut3	Gentamicin
149500	pFVGe2	RSF1010	$VanR^{AM}$	Gentamicin	Kanamycin
149497	pILxR5	Tn7 integration	LuxR	GFPmut3	Gentamicin
149493	pKCyR6	RK2	$CymR^{AM}$	mRFP	Spectinomycin
149494	pKCyR8	RK2	$CymR^{AM}$	mRFP	Hygromycin
149492	pWLlR5	pSA	LacI	mRFP	Gentamicin
167517	pBLxR4	pBBR	LuxR	mRFP	Carbenicillin
167518	pFLxR3	RSF1010	LuxR	mRFP	Tetracycline
167521	pFCiKm7	RSF1010	$CinR^{AM}$	Kanamycin	Erythromycin

Plasmids are organized by experiment and order that they appear in the main text.

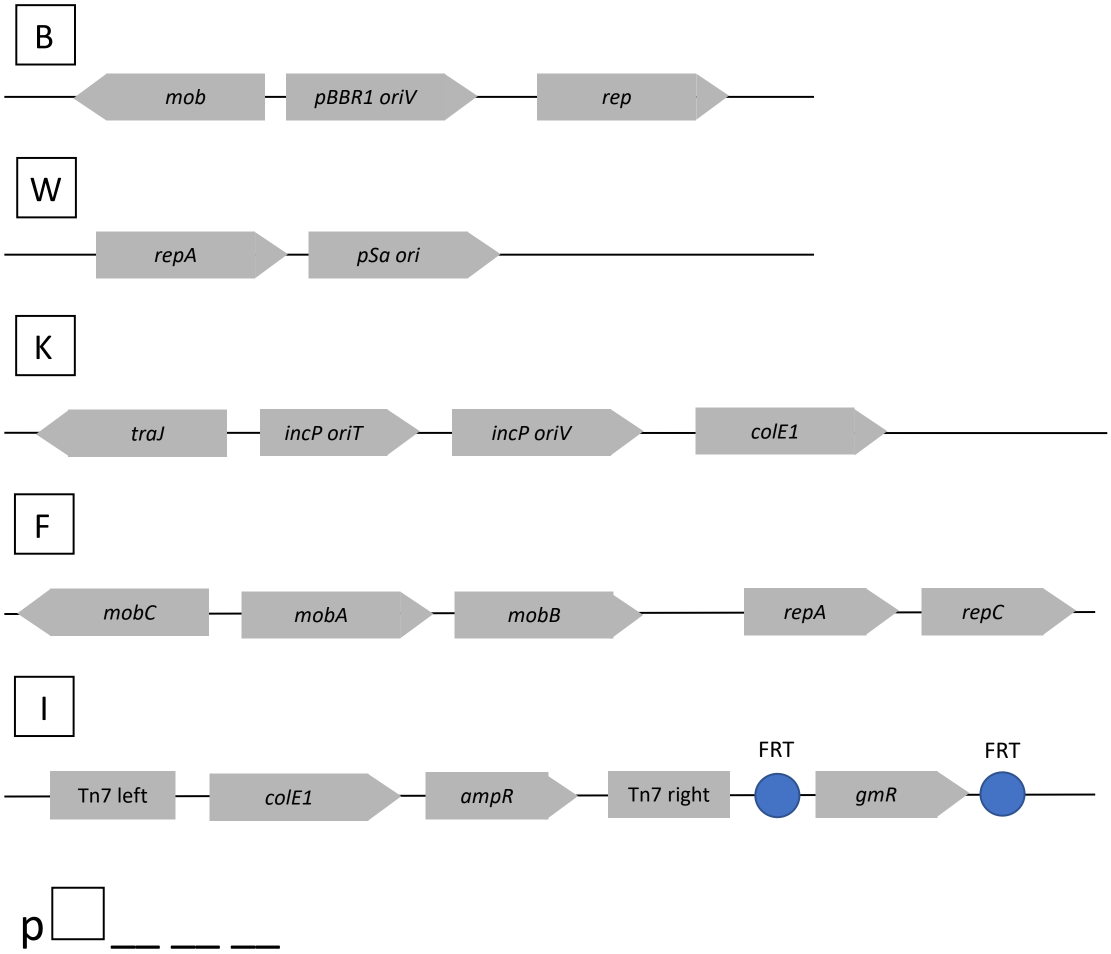

Supplementary Figure 1: Origin Parts.
Broad host-range origins included in plasmid toolbox: pBBR (1) (B), pSa (2) (W), RK2 (3) (K), RSF1010 (4) (F), and Tn7 integration (5) (I).

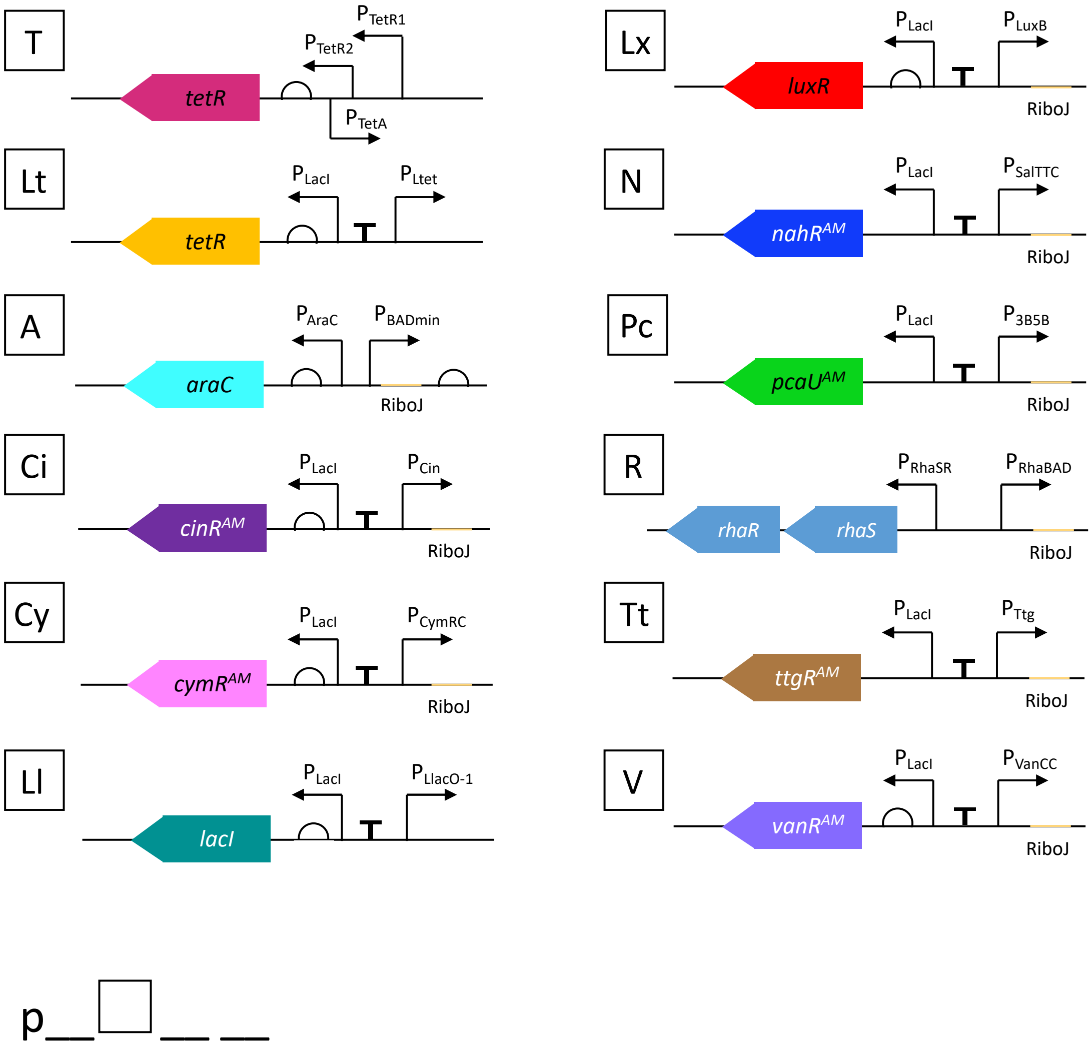

Supplementary Figure 2: Inducible promoter-regulator parts.
12 inducible promoter-regulator pairs included in the plasmid toolbox. Each part includes a transcriptional regulator and associated promoter as well as the cognate regulated promoter. Ribosome binding sites, terminators, and RiboJ are indicated where applicable. VanR contains an A101S mutation relative to the source sequence, though we have no evidence that this mutation affects function.

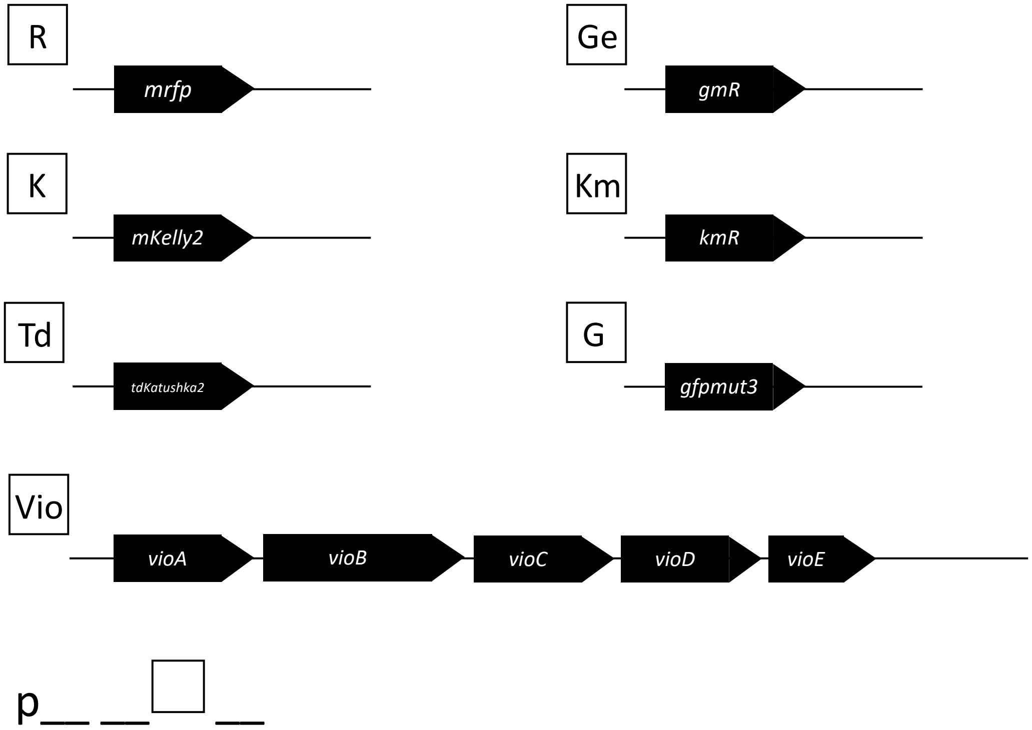

Supplementary Figure 3: Reporter Parts.
Reporter parts included in the plasmid toolbox including four fluorescent proteins, two antibiotic resistance genes, and violacein pathway (4).

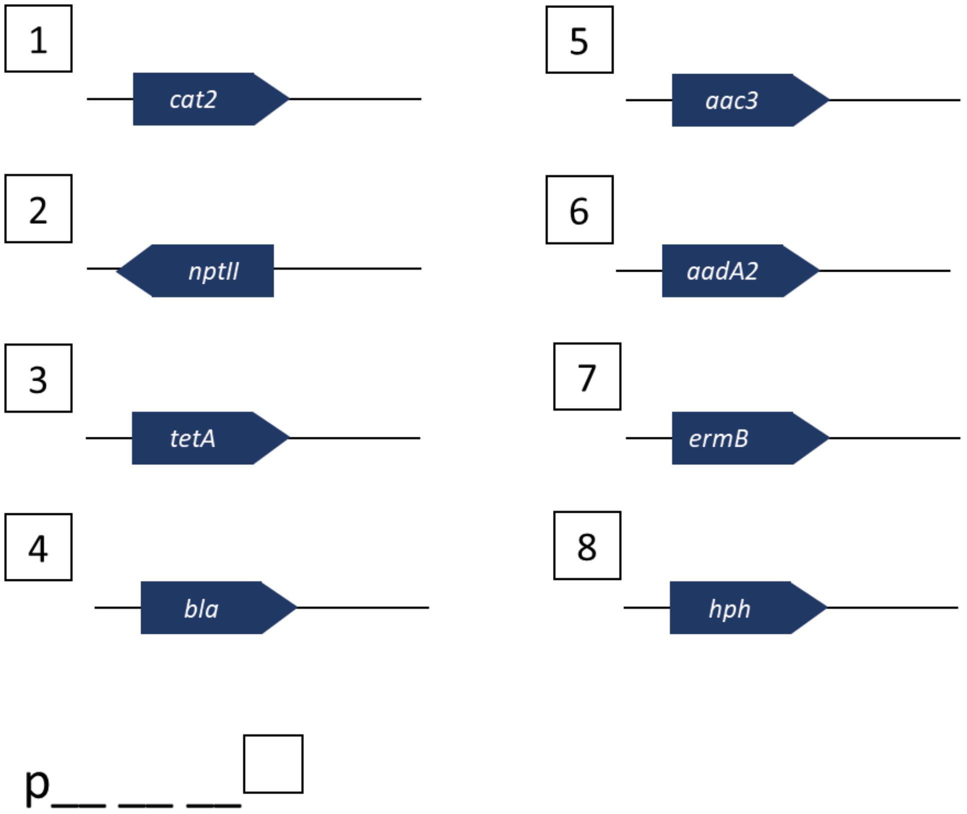

Supplementary Figure 4: Marker Parts.
Antibiotic resistance marker parts included in plasmid toolbox including chloramphenicol resistance (1) (*cat2*), kanamycin resistance (1) (*nptII*), tetracycline resistance (1) (*tetA*), ampicillin resistance (1) (*bla*), gentamicin resistance (1) (*aac3*), streptomycin resistance (6) (*aadA2*), erythromycin resistance (7) (*ermB*), and hygromycin resistance (8) (*hph*).

Supplementary Table 2: Regulatory Element Sources

Part	Native Host	Regulator Source	Promoter Source
AraC/P_{BADmin}	*Escherichia coli*	pSLTS (9, 10)	pSLTS (9–11)
$CinR^{AM}/P_{Cin}$	*Rhizobium leguminosarum*	sAJM.1506 (10, 12, 13)	Synthetic (10)
$CymR^{AM}/P_{CymRC}$	*Pseudomonas putida*	sAJM.1506 (10, 14, 15)	Synthetic (10, 14)
LacI/$P_{LlacO-1}$	*Escherichia coli*	pTacHis	iGEM
LuxR/P_{LuxB}	*Aliivibrio fischeri*	sAJM.1506 (10, 16)	Synthetic (10, 16)
$NahR^{AM}/P_{SalTTC}$	*Pseudomonas putida*	sAJM.1506 (10, 17)	Synthetic (10, 18)
$PcaU^{AM}/P_{3B5B}$	*Acinetobacter* sp. ADP1	sAJM.1506 (10, 19, 20)	Synthetic (10, 19)
RhaS-RhaR/P_{RhaBAD}	*Escherichia coli*	*E. coli*	*E. coli*
TetR/$P_{LtetO-1}$	*Escherichia coli*	pSLTS (9)	iGEM Registry (21)
TetR/P_{TetA}	*Escherichia coli*	pSLTS (9)	pSLTS (9)
$TtgR^{AM}/P_{Ttg}$	*Pseudomonas putida*	sAJM.1506 (10, 15, 22)	Synthetic (10, 22, 23)
$VanR^{AM}/P_{VanCC}$	*Caulobacter crescentus*	sAJM.1506 (10, 24, 25)	Synthetic (10, 25)

Supplementary Table 3: Strain Growth Conditions

**Strain**	**Medium**	**Incubation**	**Gentamycin Concentration**	**Kanamycin Concentration**
Acinetobacter baylyi ADP1	LB	30° C	20 µg/mL	50 µg/mL
Agrobacterium fabrum C58	LB	30° C	100 µg/mL	250 µg/mL
Burkholderia thailandensis E264	LSLB	37° C	100 µg/mL	250 µg/mL
Pseudomonas aeruginosa PAO1	LB	37° C	100 µg/mL	500 µg/mL
Pseudomonas putida KT2440	LB	30° C	100 µg/mL	250 µg/mL
Ruegeria sp. TM1040	½ YTSS	30° C	50 µg/mL	50 µg/mL
Sulfitobacter sp. EE-36	½ YTSS	30° C	20 µg/mL	50 µg/mL
Xanthomonas campestris ATCC 33913	LB	30° C	20 µg/mL	50 µg/mL
Aliivibrio fischeri ES114	LBS	30° C	200 µg/mL	NA

Supplementary Table 4: Electroporation Conditions

**Strain**	**Electroporation Voltage**	**Recovery Media**
Agrobacterium fabrum C58	2.20 kV, 1 pulse	LB
Burkholderia thailandensis E264	1.8 kV, 1 pulse	LSLB
Pseudomonas aeruginosa PAO1	1.8 kV, 1 pulse	LB
Pseudomonas putida KT2440	1.8 kV, 1 pulse	LB
Ruegeria sp. TM1040	2.20 kV, 1 pulse	½ YTSS
Sulfitobacter sp. EE-36	2.20 kV, 1 pulse	½ YTSS
Xanthomonas campestris ATC 33913	1.8 kV, 1 pulse	LB

Supplementary Table 5: Inducers

Inducer	Source	Solvent	Stock	Standard concentration
Arabinose	Sigma A3256	Water	20%	4 mM
hydroxytetradecanoyl-homoserine lactone (OHC14)	Sigma K3007	DMF	10 mM	10 µM
Cumate	Sigma 268402	EtOH	100 mM	100 µM
IPTG	Sigma I6758	Water	1 M	1 mM
N-(3-oxohexanoyl) homoserine lactone (OC6)	Sigma 51481	DMF	10 mM	10 µM
Salicylic acid	Acros Organics AC220980500	Water	100 mM	100 µM
Protocatechuate	Sigma P5630	EtOH	1 M	1 mM
Anhydrotetracycline	Acros Organics AC233131000	EtOH	100 µM	100 nM
Rhamnose	Sigma R3875	Water	10%	0.2%
Naringenin	Sigma N5893	DMSO	1 M	1 mM
Vanillate	Sigma V2250	EtOH	100 mM	100 µM

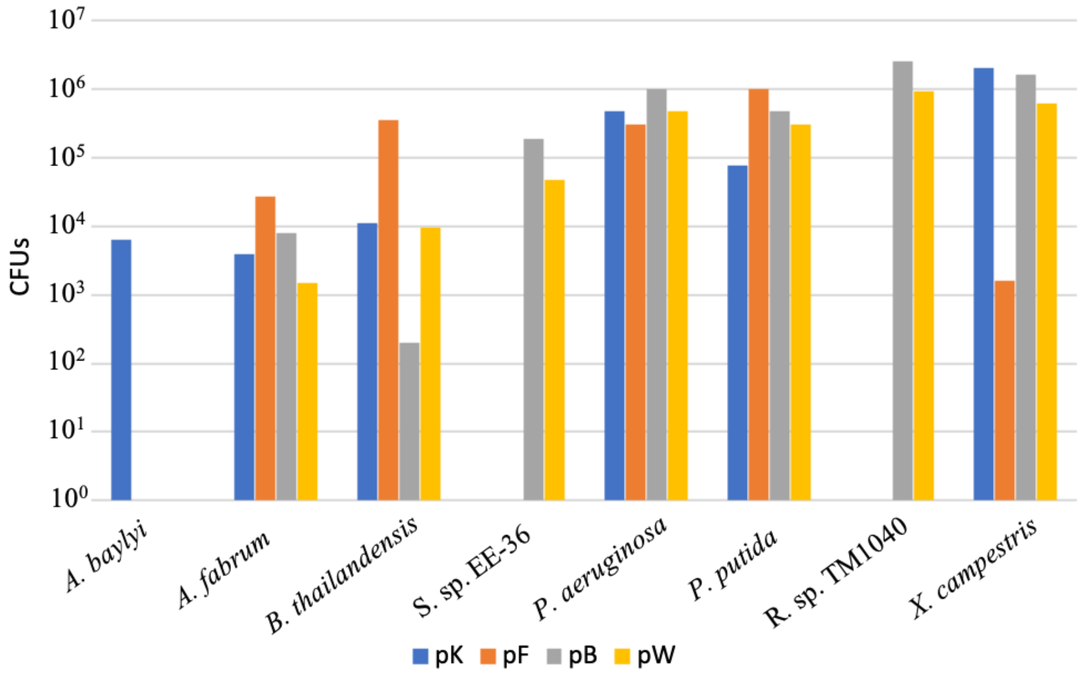

Supplementary Figure 5: Plasmid Transformation Efficiencies.
Transformation efficiency was calculated after electroporation of each strain with each of four plasmids that were identical with the exception of the origin of replication.

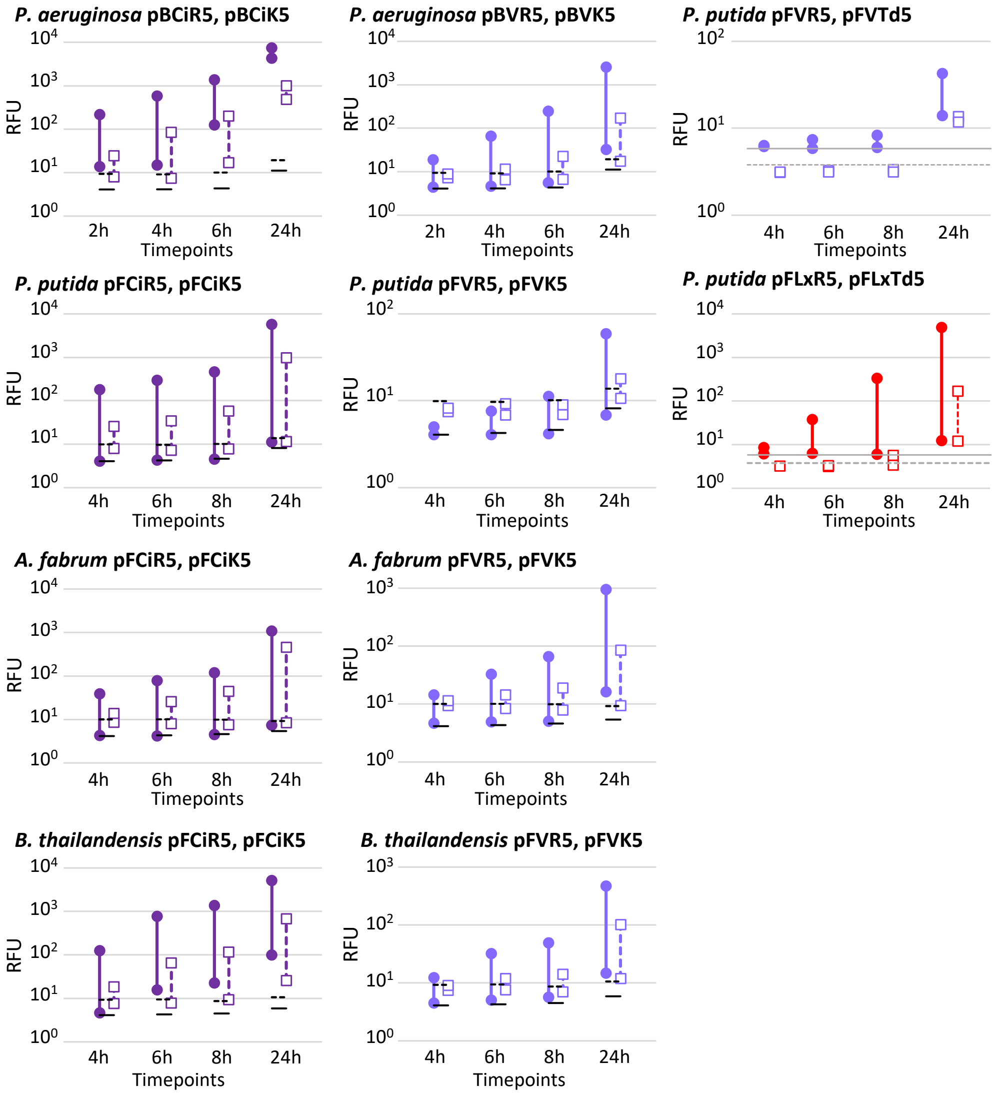

Supplementary Figure 6: Red and Far-Red Reporter Comparison.

Different fluorescent protein reporters were tested to determine which had the largest range of expression in the bacterial strains under study. Plasmid backbones are identical in each screen apart from the reporter gene. Colored vertical lines represent fluorescence from an mRFP (R) reporter and dashed vertical lines represent fluorescence from an mKelly2 (K) or tdKatushka (Td) reporter. Data is shown for four timepoints and output is in RFU. Data represents averages of three replicates. The black dashed and solid horizontal bar represents fluorescence from an empty vector control for mKelly2 or tdKatushka2 and mRFP, respectively.

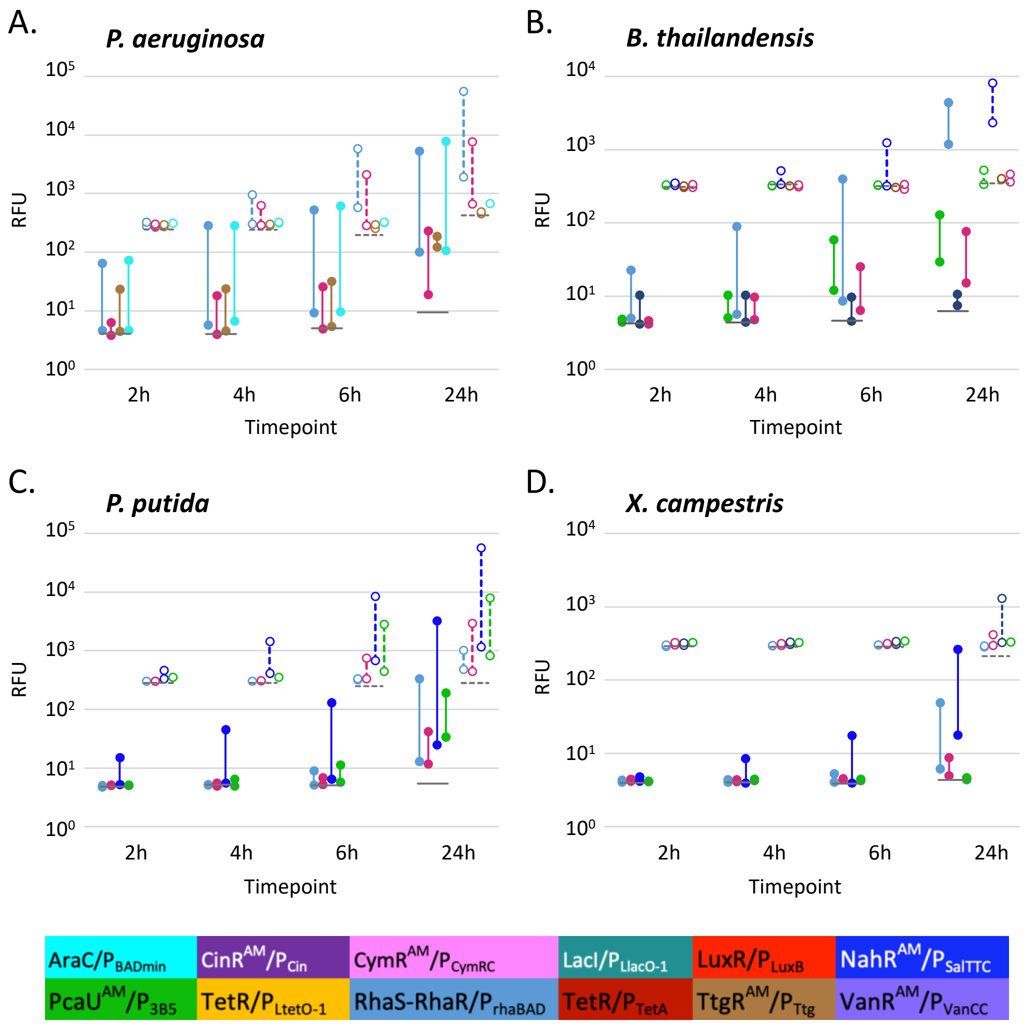

Supplementary Figure 7: Green and Red Reporter Comparison.
Expression in the presence and absence of inducer at four timepoints in *P. aeruginosa* (A.), *B. thailandensis* (B.), *P. putida* (C.), and *X. campestris* (D.). Expression from mRFP is shown in closed circles and solid lines, expression from GFPmut3 shown in open circles and dashed lines. Corresponding negative controls for mRFP and GFPmut3 are shown in horizontal solid and dashed lines, respectively. Expression plotted in RFU as an average of three replicates.

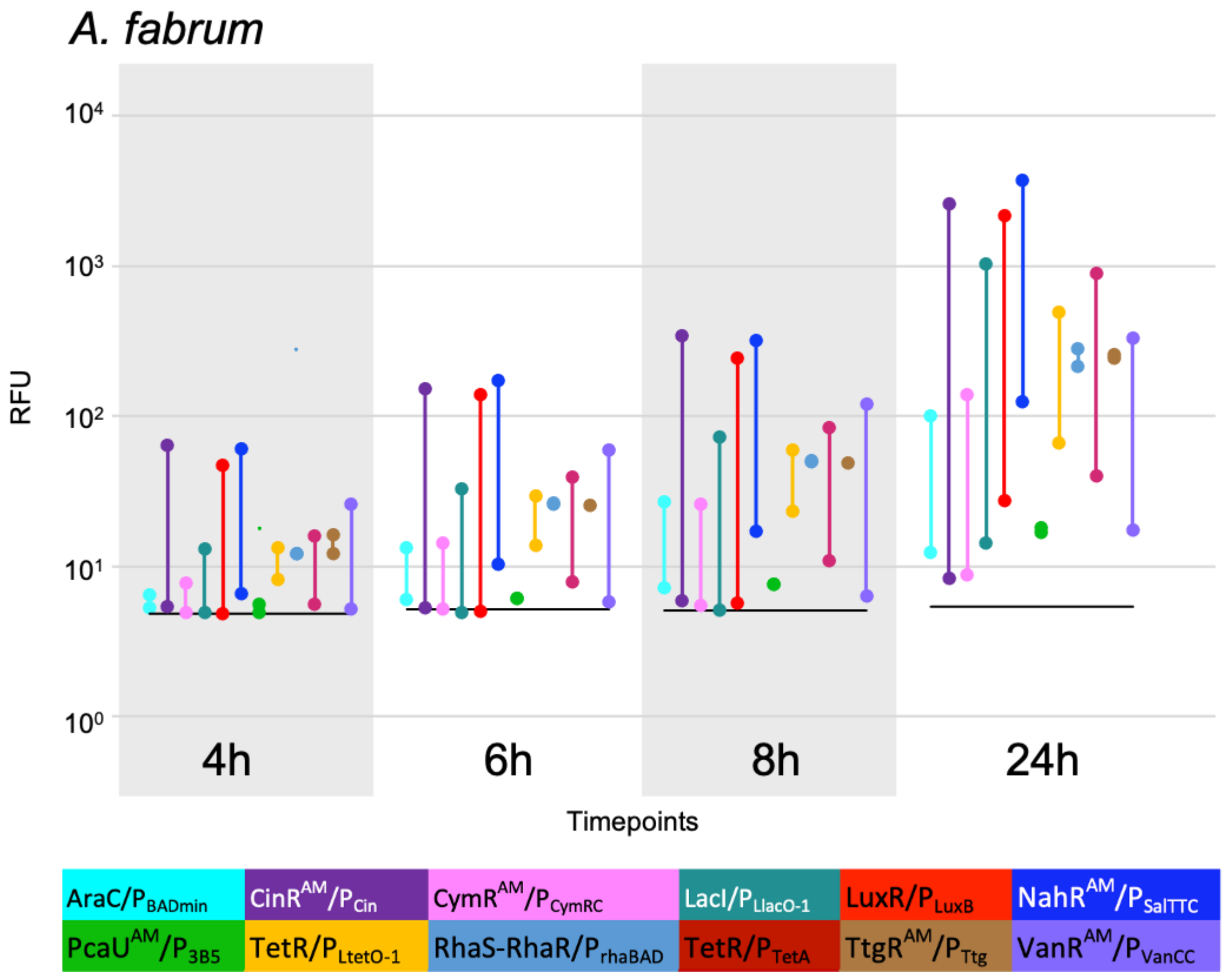

Supplementary Figure 8: 12 Inducible Systems in *A. fabrum* with plasmid pF_R5.
Data from four timepoints with and without inducer of inducible system screen given in raw average RFU. Data is an average of three replicates. Horizontal black line is the RFU from an empty vector control.

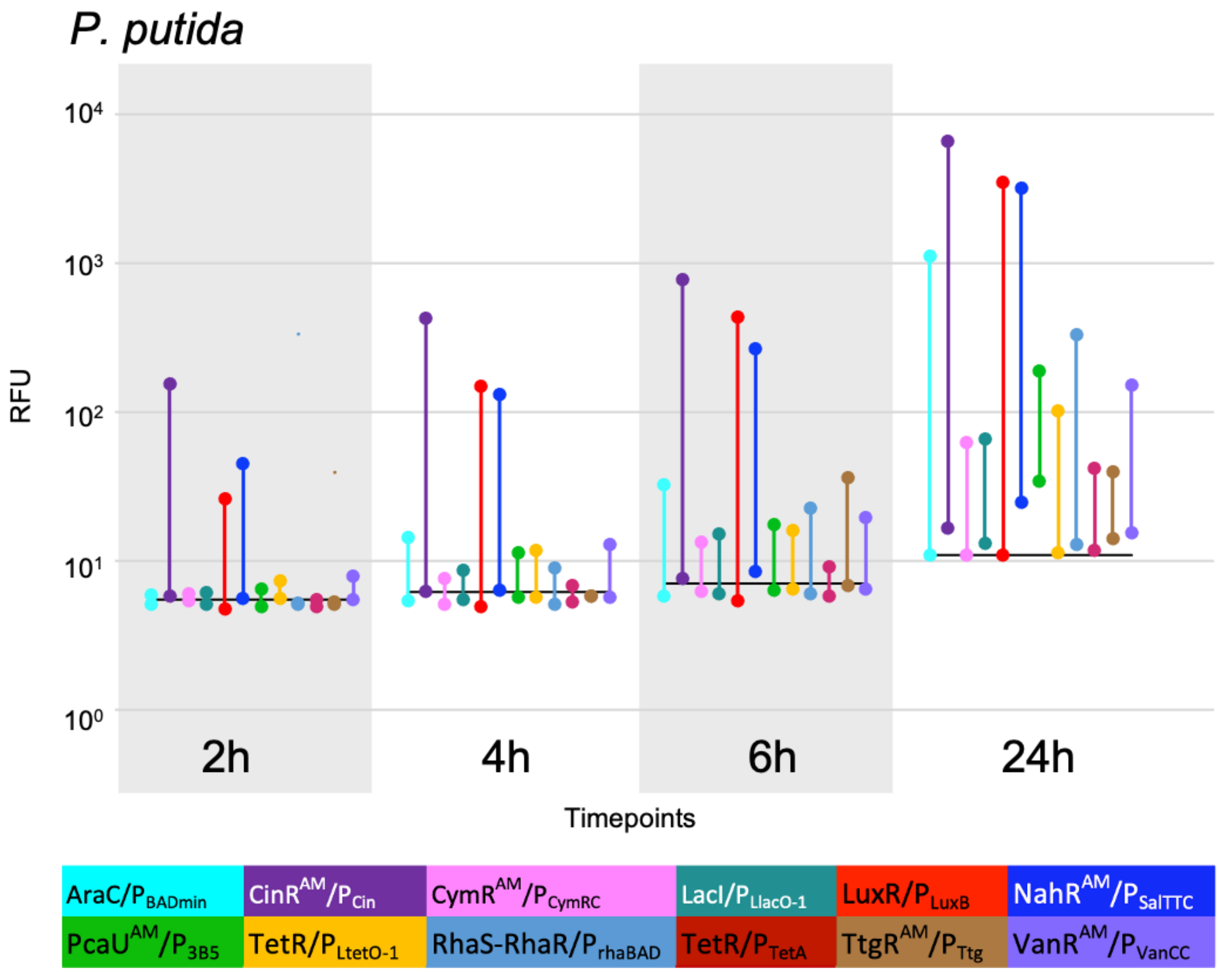

Supplementary Figure 9: 12 Inducible Systems in *P. putida* with plasmid pF_R5.
Data from four timepoints with and without inducer of inducible system screen given in raw average RFU. Data is an average of three replicates. Horizontal black line is the RFU from an empty vector control.

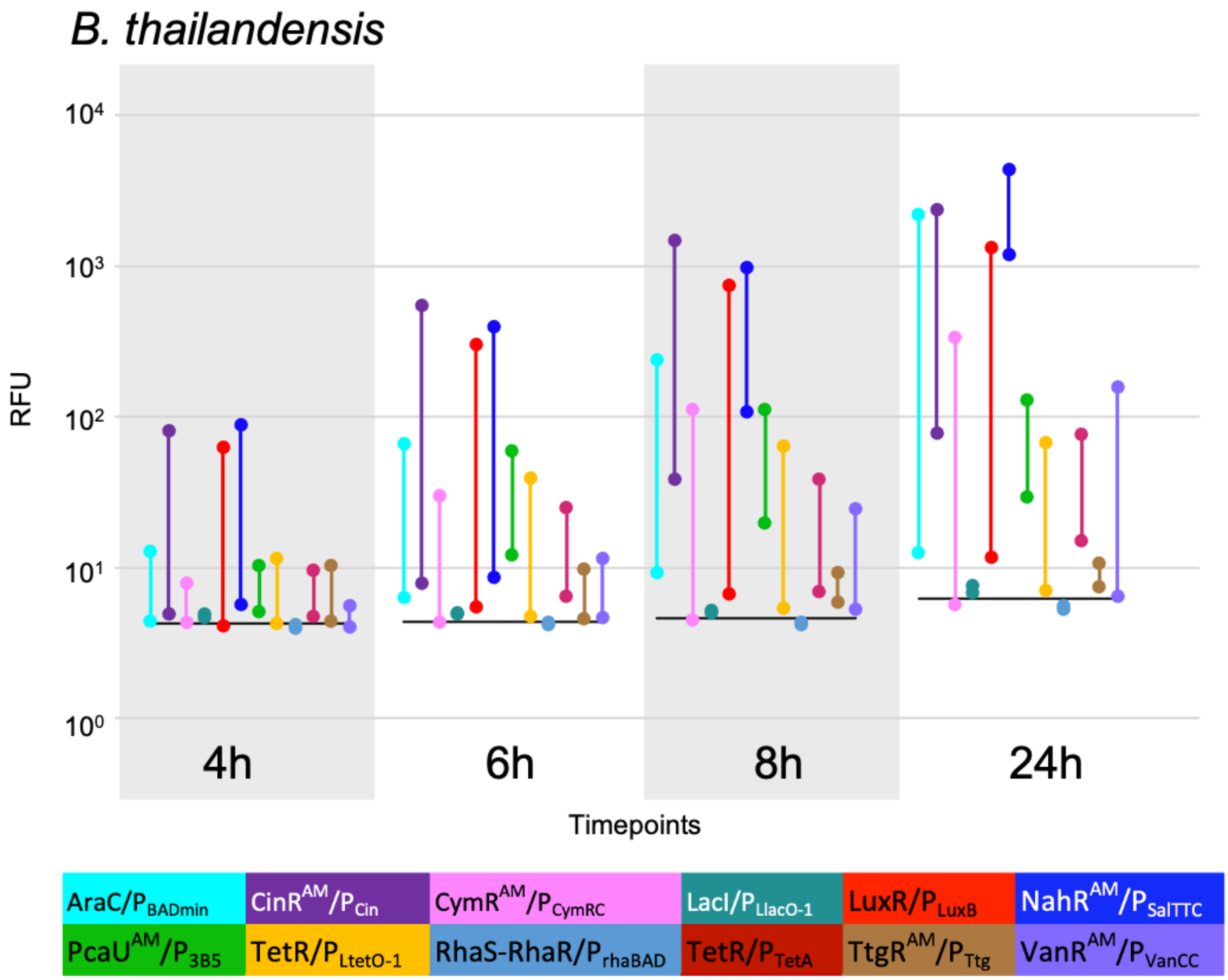

Supplementary Figure 10: 12 Inducible Systems in *B. thailandensis* with plasmid pF_R5. Data from four timepoints with and without inducer of inducible system screen given in raw average RFU. Data is an average of three replicates. Horizontal black line is the RFU from an empty vector control.

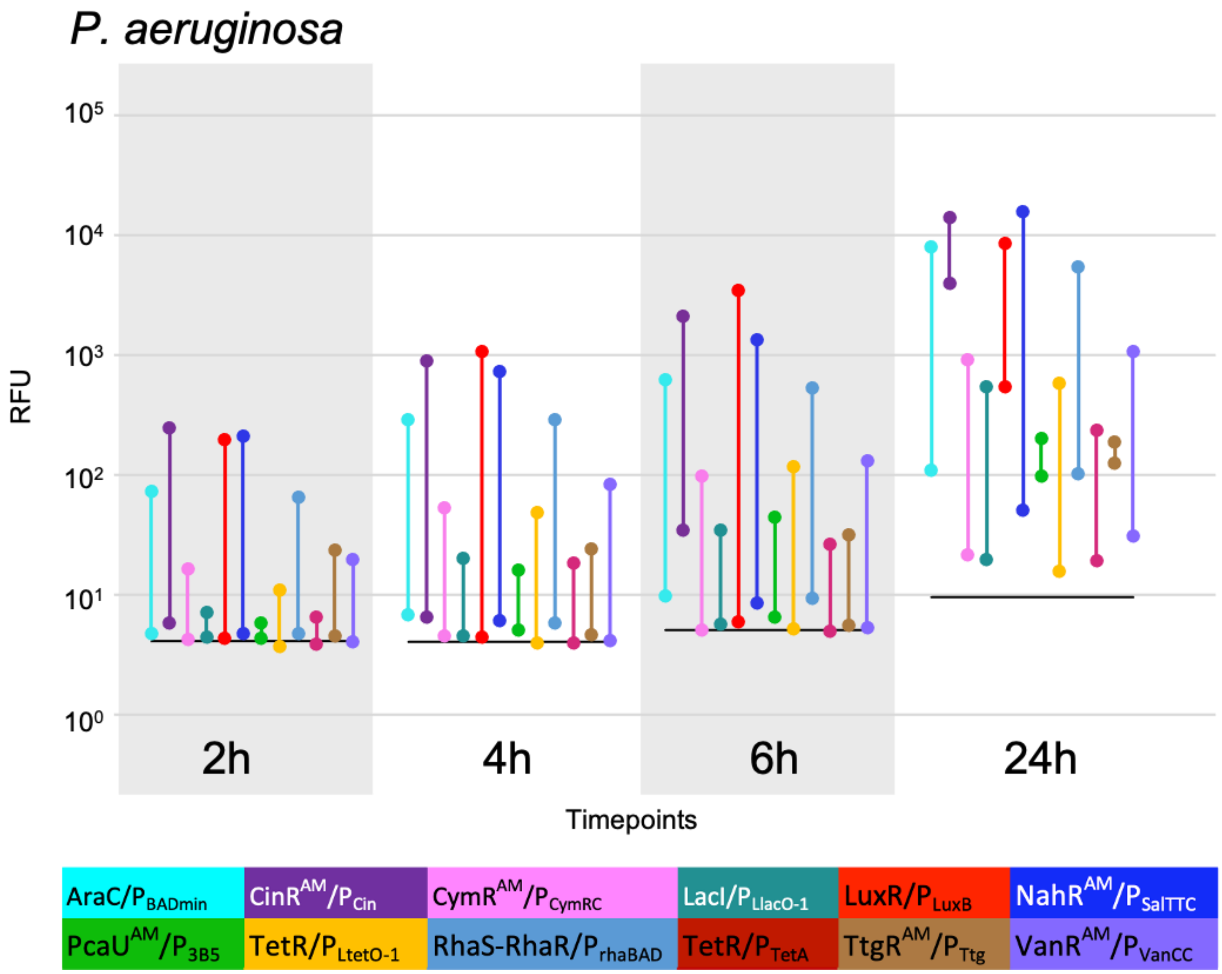

Supplementary Figure 11: 12 Inducible Systems in *P. aeruginosa* with plasmid pB_R5. Data from four timepoints with and without inducer of inducible system screen given in raw average RFU. Data is an average of three replicates. Horizontal black line is the RFU from an empty vector control.

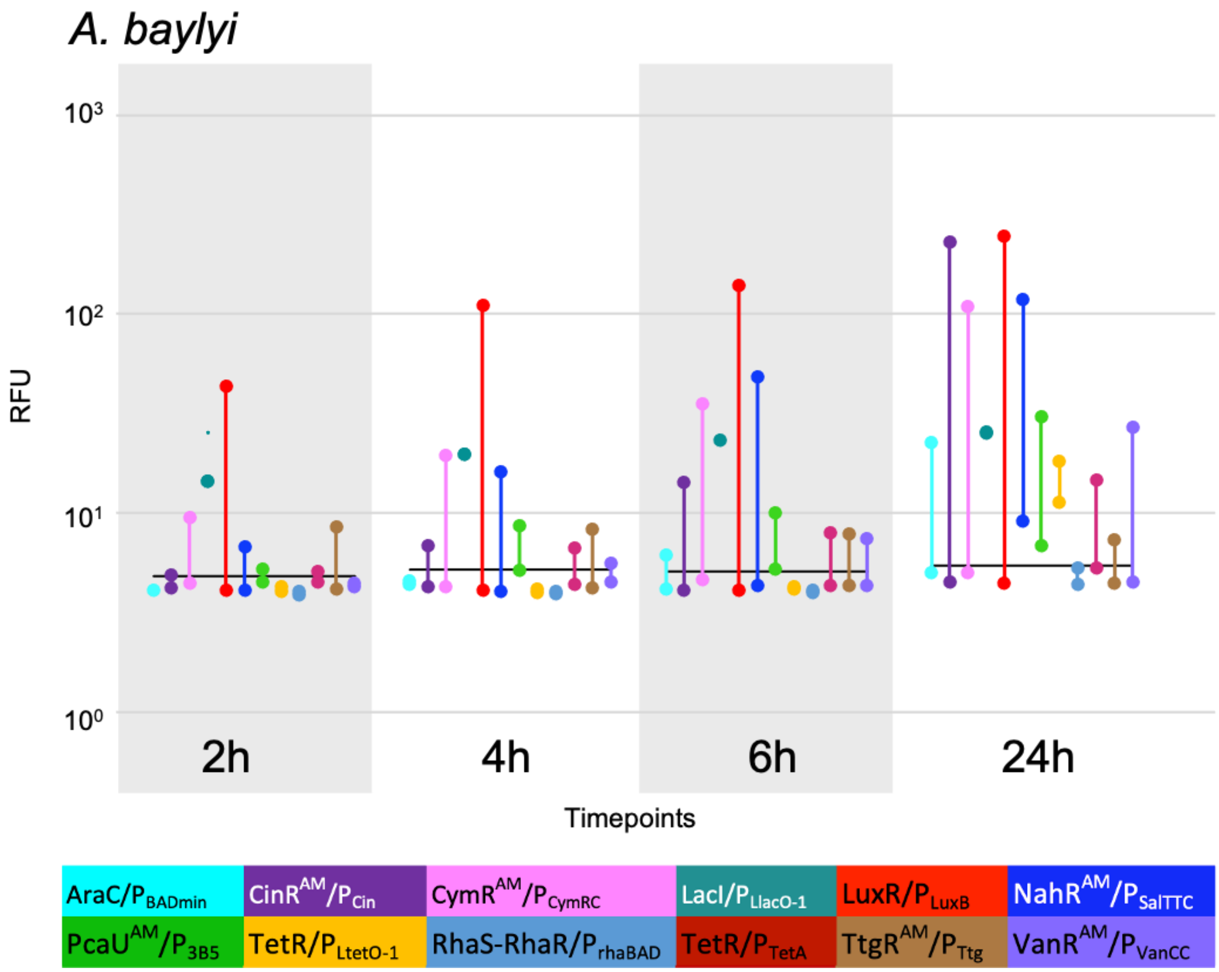

Supplementary Figure 12: 12 Inducible Systems in *A. baylyi* with plasmid pK_R5.
Data from four timepoints with and without inducer of inducible system screen given in raw average RFU. Data is an average of three replicates. Horizontal black line is the RFU from an empty vector control.

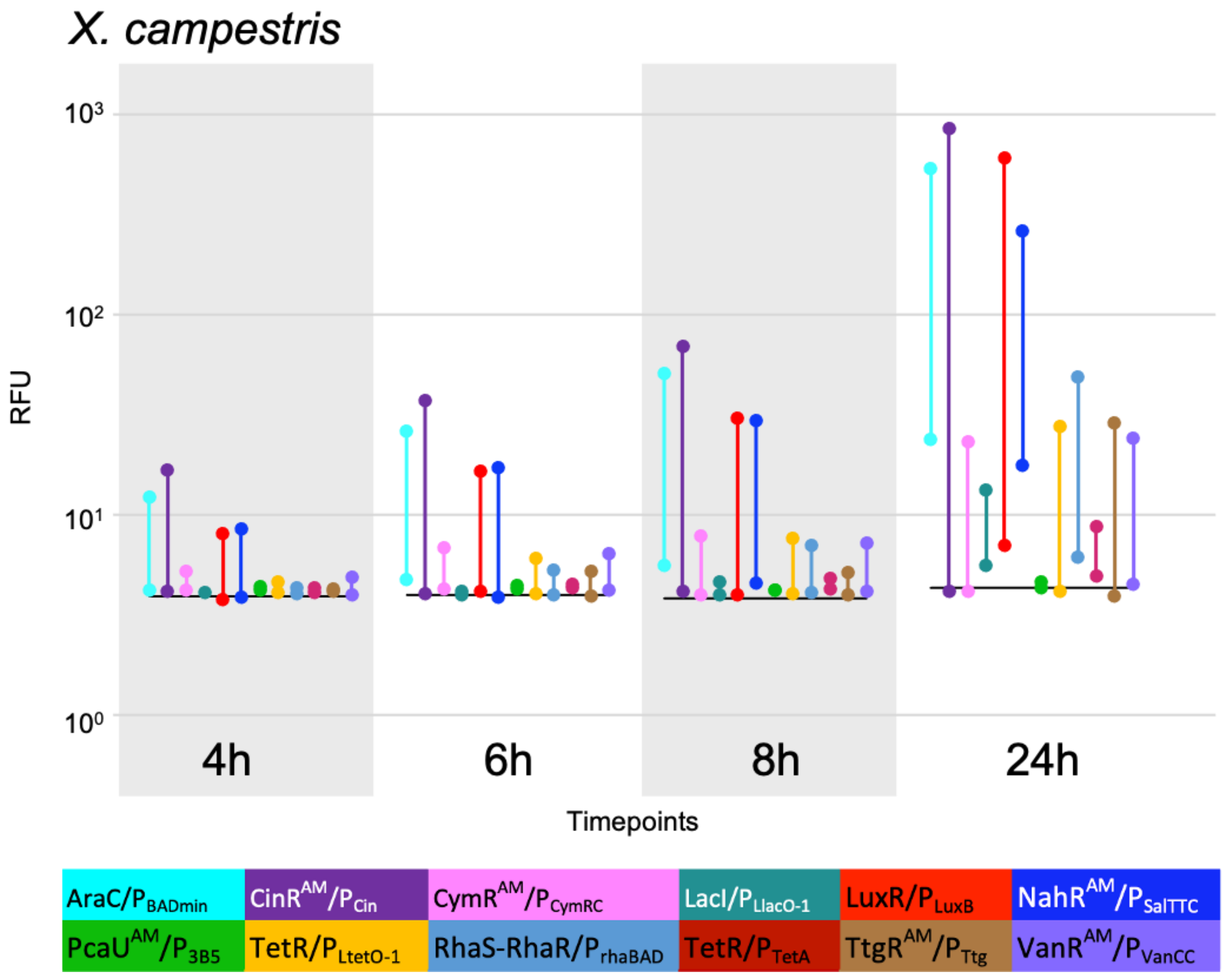

Supplementary Figure 13: 12 Inducible Systems in *X. campestris* with plasmid pK_R5. Data from four timepoints with and without inducer of inducible system screen given in raw average RFU. Data is an average of three replicates. Horizontal black line is the RFU from an empty vector control.

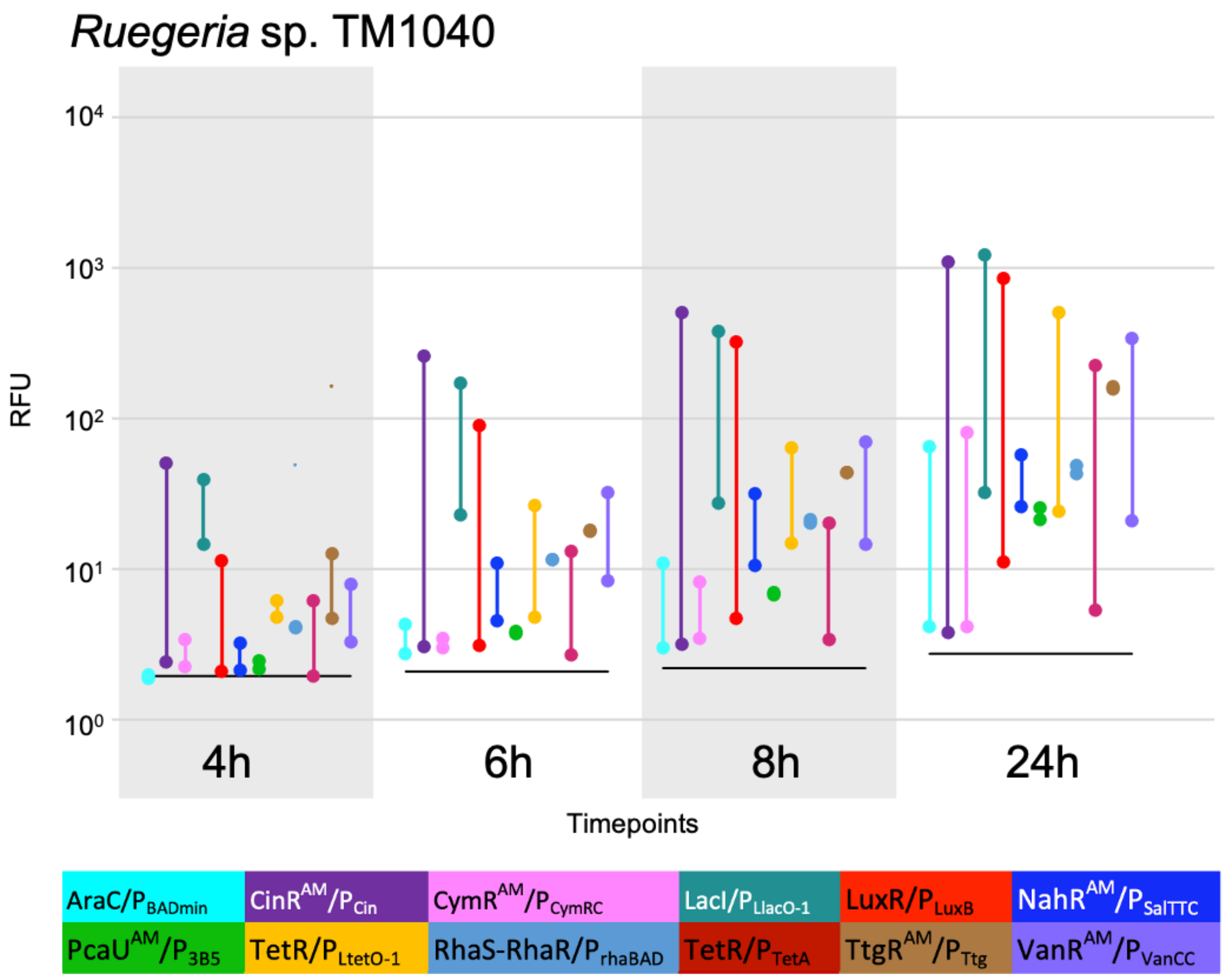

Supplementary Figure 14: 12 Inducible Systems in *Ruegeria* sp. TM1040 with plasmid pB_R5. Data from four timepoints with and without inducer of inducible system screen given in raw average RFU. Data is an average of three replicates. Horizontal black line is the RFU from an empty vector control.

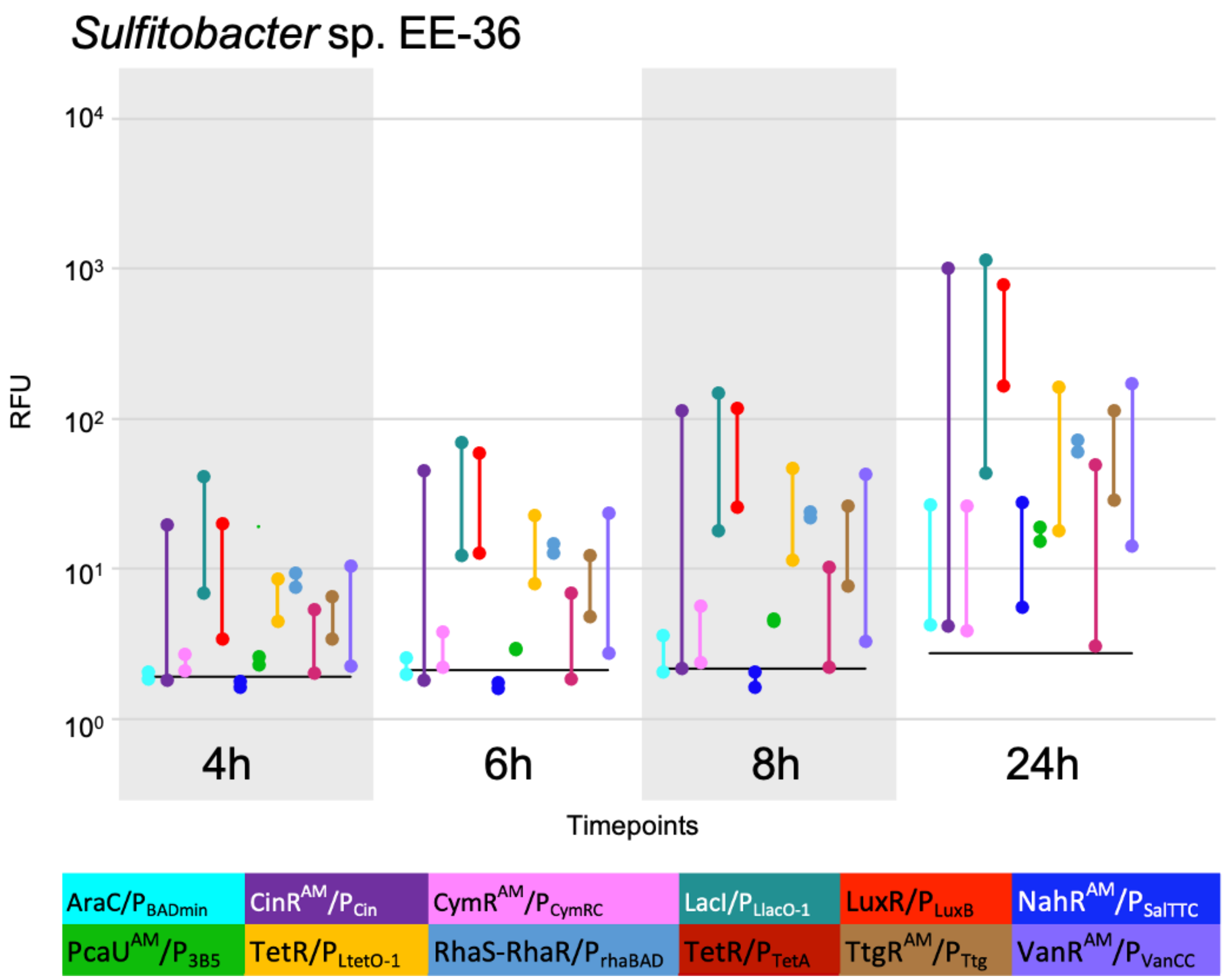

Supplementary Figure 15: 12 Inducible Systems in *Sulfitobacter* sp. EE-36 with plasmid pB_R5.
Data from four timepoints with and without inducer of inducible system screen given in raw average RFU. Data is an average of three replicates. Horizontal black line is the RFU from an empty vector control.

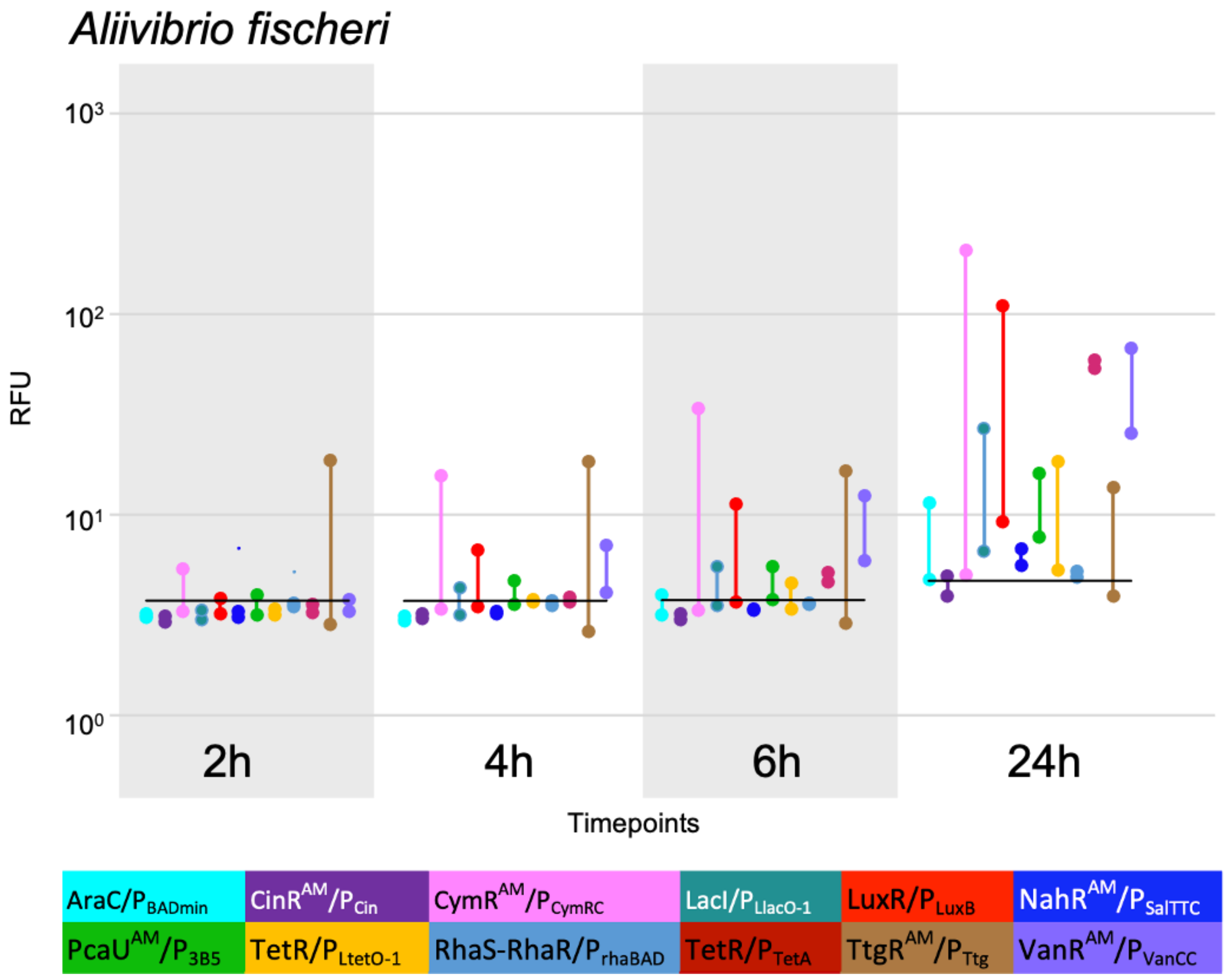

Supplementary Figure 16: 12 Inducible Systems in *Aliivibrio fischeri* with plasmid pF_R5. Data from four timepoints with and without inducer of inducible system screen given in raw average RFU. Data is an average of three replicates. Horizontal black line is the RFU from an empty vector control.

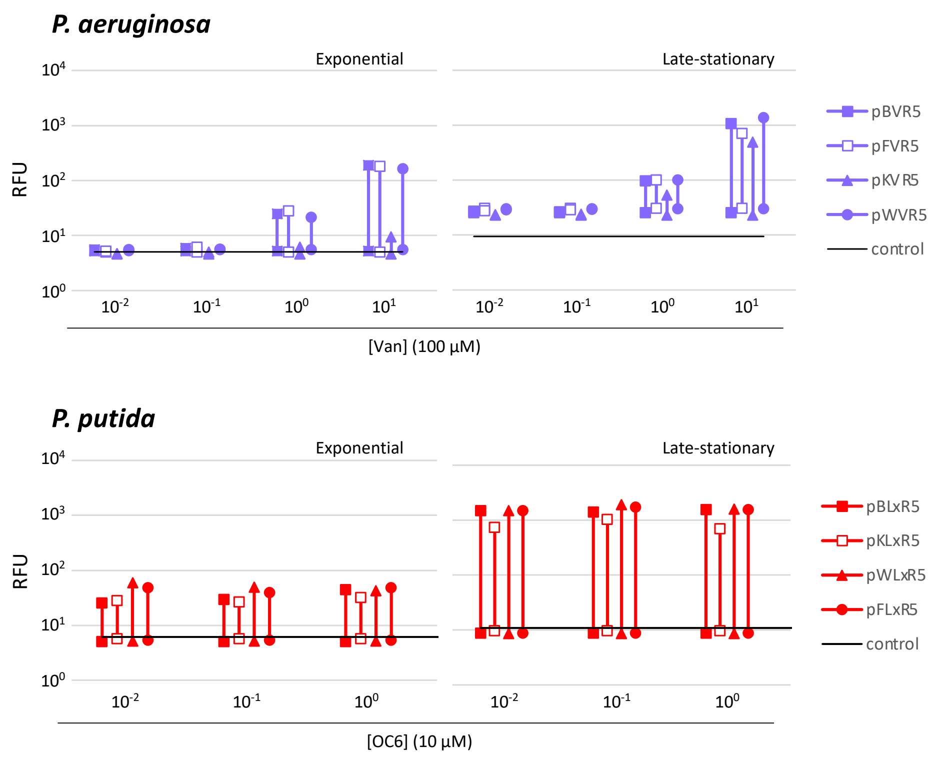

Supplementary Figure 17: Comparison of mRFP Expression with Different Origins of Replication.

Plasmid backbones in each screen are identical apart from the broad-host-range origin. Expression is given in RFU and shown without inducer or with titrated inducer concentrations in exponential and late-stationary phase for *P. aeruginosa* and *P. putida*. Data represents averages of three replicates. The negative control strain contains an identical plasmid lacking reporter gene.

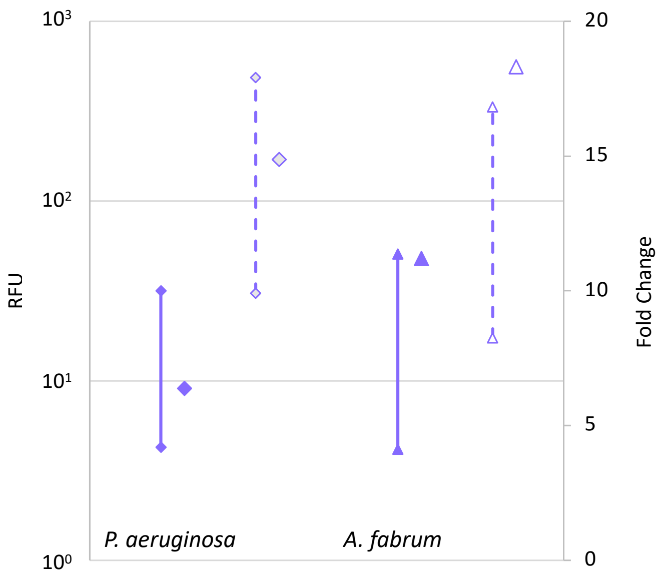

Supplementary Figure 18: Measurement of mRFP from the Tn7 Integration Vector and a replicating plasmid in *P. aeruginosa* and *A. fabrum*.

Solid vertical lines represent the induction range of mRFP in the presence and absence of inducer from integrated $VanR^{AM}/P_{VanCC}$ after overnight growth in *P. aeruginosa* (left) and *A. fabrum* (right) induced with 100 µM and 1 mM of vanillate, respectively. Dashed vertical lines represent induction range from plasmid-based $VanR^{AM}/P_{VanCC}$ systems, pBVR5 for *P. aeruginosa* and pFVR5 for *A. fabrum*. Open symbol data points represent the corresponding fold-change.

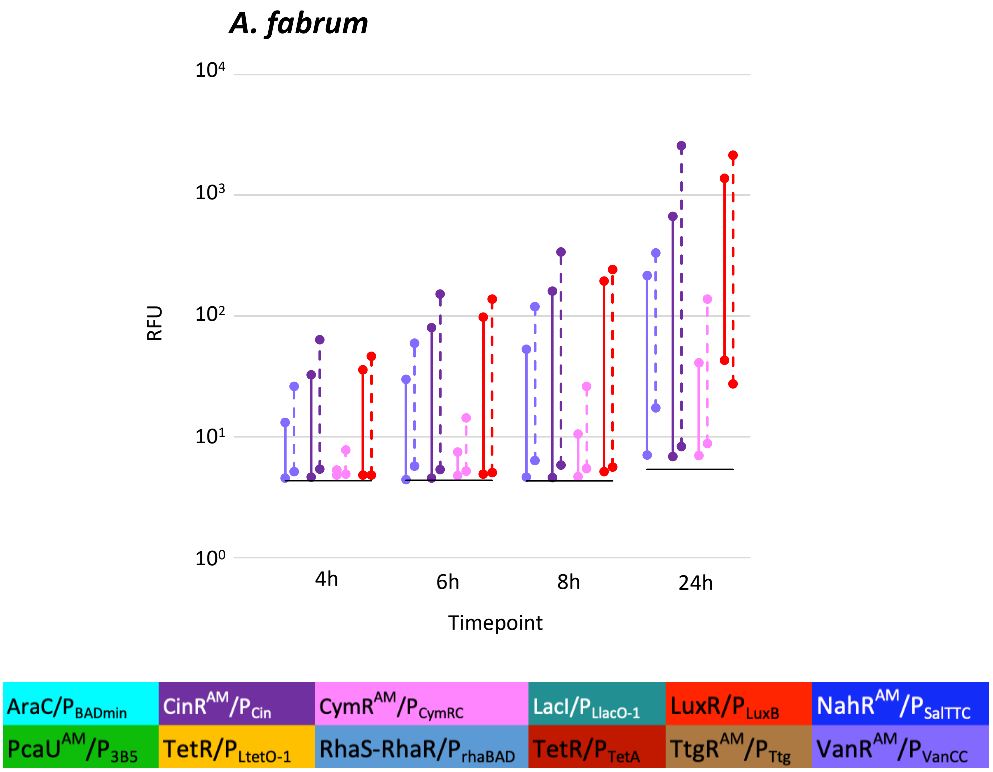

Supplementary Figure 19: Comparison of Expression with Different Antibiotic Markers. Data from four promoter-regulator pairs on a plasmid backbone with a kanamycin resistance marker shown in solid lines. Data from identical plasmids on a gentamicin resistance marker is shown in dashed lines. Fluorescence is measured in raw RFU and represented as the average of three technical replicates. Horizontal black bar represents fluorescence from a plasmid lacking a reporter gene.

Supplementary Table 6: Independent Expression Experiments

A. fabrum	*P. putida*	*P. aeruginosa*
pBCiG2 + pFLlR5	pBLlG2 + pFLtR5	pBAR5 + pKCyG2
pBCiG2 + pFAR5	pBLlG2 + pFLxR5	pBLlR5 + pKCyG2
pBCiG2 + pFCyR5	pBLlG2 + pFVR5	pBNR5 + pKCyG2
pBCiG2 + pFVR5	pBLlG2 + pFRR5	pBLtR5 + pKCyG2
pBCiG2 + pFLxR5	pBLlG2 + pFNR5	pBRR5 + pKCyG2
pBLlG2 + pFAR5	pBLlG2 + pFCiR5	pWCyG2 + pBRR5
	pBLlG2 + pFAR5	pBVR5 + pKCyG2
		pBLxR5 + pKCyG2
		pKLxG2 + pBRR5

Plasmid combinations for two-plasmid system experiments listed top to bottom in the order they appear on Figure 4.

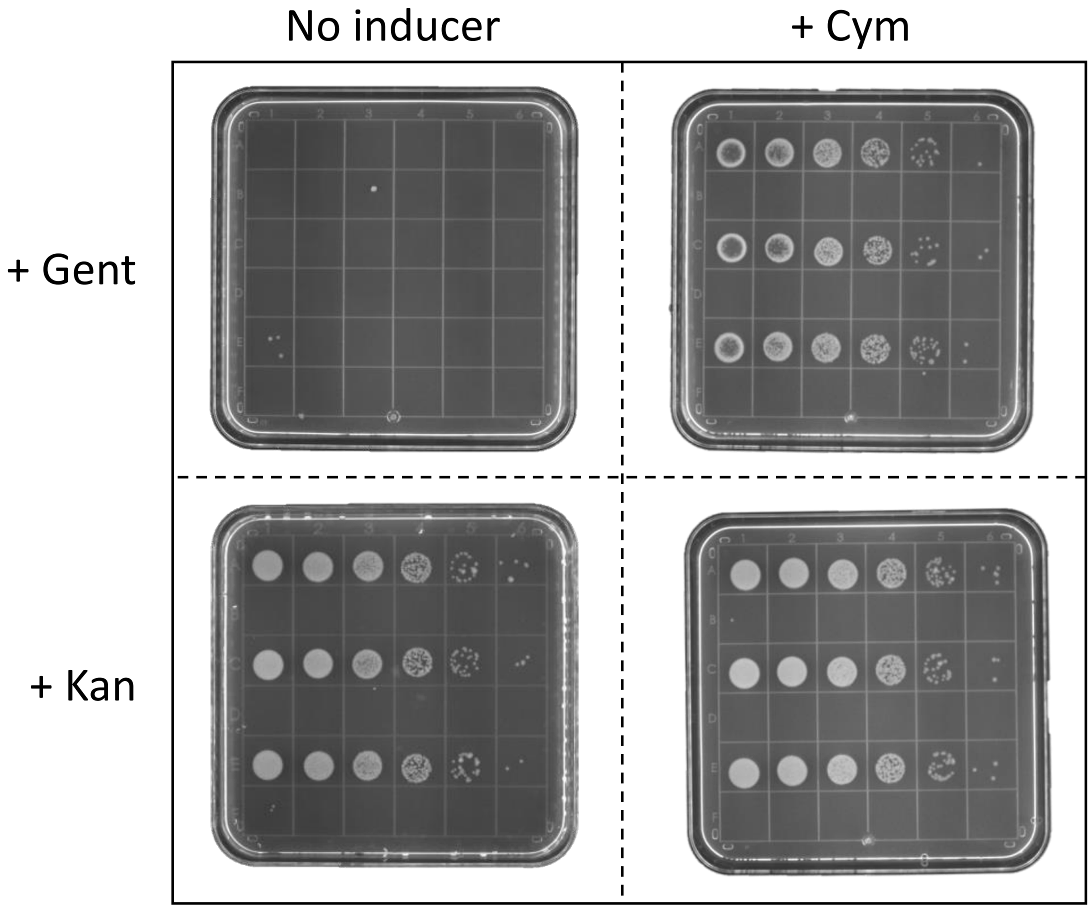

Supplementary Figure 20: Controlled Expression of the *aacC1* Conditionally Essential Gene. *B. thailandensis* pFCyGe2 spotted during mid-exponential growth. Cultures that grew in the absence of inducer were spotted on both an agar plate containing gentamicin (top left quadrant) and a plate containing kanamycin (bottom left quadrant). Induced cultures were spotted on an agar plate containing gentamicin and 100 µM cumate (top right quadrant) and a plate containing kanamycin and 100 µM cumate (bottom right quadrant). Three replicates are spotted on to each plate, with undiluted cultures spotted on to the first, third, and fifth rows and serially diluted ten-fold through 10^{-11}.

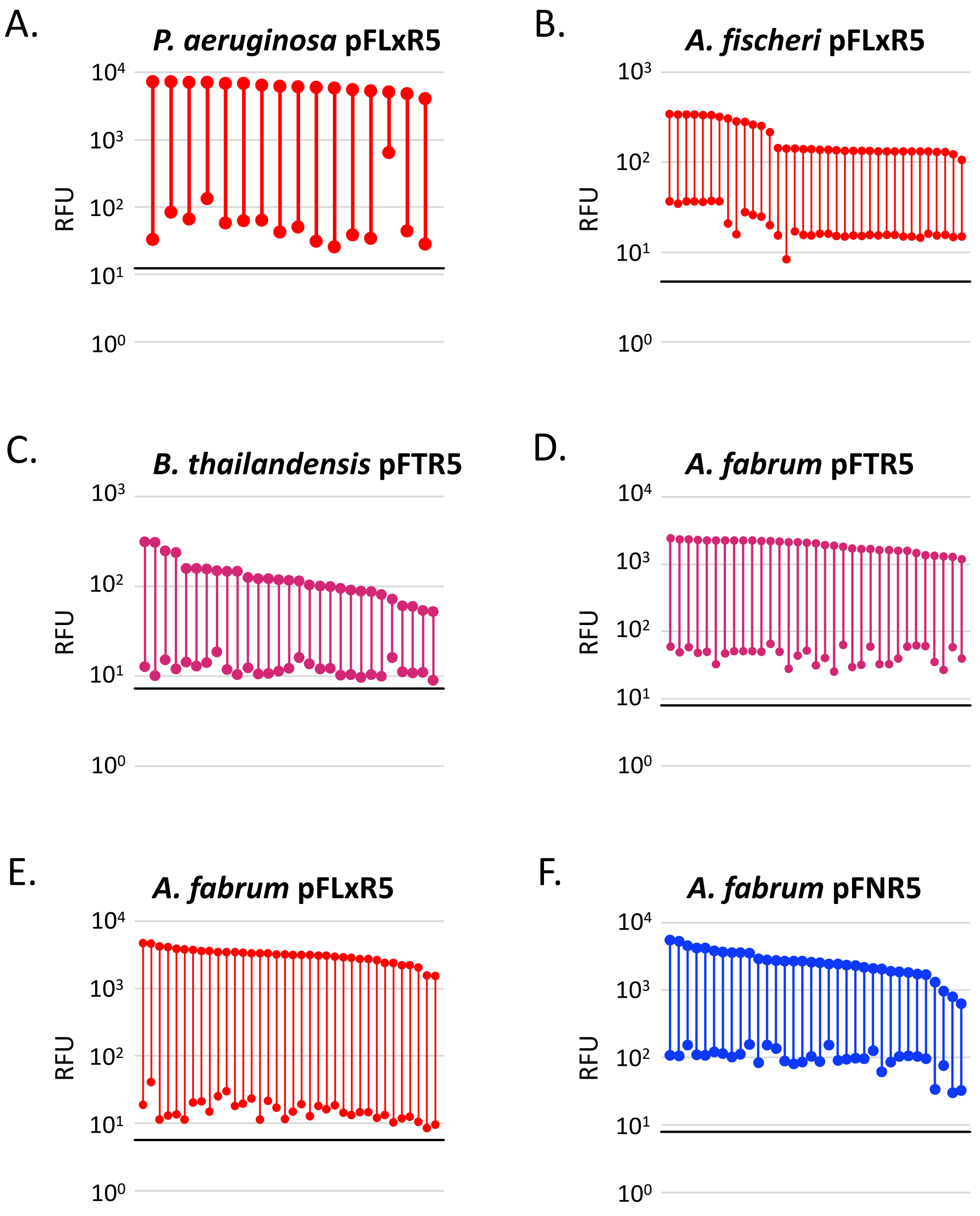

Supplementary Figure 21: Expression Range of Select Library Isolates.
Fluorescence data for LuxR/P_{LuxB} library isolates in *P.aeruginosa* (A.), *A. fischeri* (B.), and *A. fabrum* (E.), TetR/P_{TetA} library isolates in *B. thailandensis* (C.) and *A. fabrum* (D.), and NahR^{AM}/P_{SalTTC} library isolates in *A. fabrum* (F). Range of expression from original plasmid represented in shaded box, fluorescence from empty vector control in black horizontal line. Data is sorted by induced RFU and is the average of three replicates.

Supplementary Note 1: Comparison between Vector Assembly Standards

Top panel: Standard European Vector Architecture (SEVA) platform includes a standardized parts database and design principles for part assembly. Utilizing classical restriction enzyme cloning, three variable regions (cargo, replication, and antibiotic resistance marker) can be exchanged on a backbone of three connector parts (T_0 and T_1 terminators, oriT). Parts are obtained on pSEVA vectors through the SEVA website (a moderate number distributed for free) or can be generated in-house, following the SEVA format. Domestication of parts requires the removal of internal interfering restriction enzyme sites including: HindIII, PstI, XbaI, BamHI, SmaI, KpnI, SacI, SalI, EcoRI, SfiI, SphI, AvrII, PshAI, SwaI, AscI, FseI, PacI, SpeI, SanDI and NotI, with addition of the required restriction sites that flank each part category. Plasmids not adhering to the SEVA format are also available through the SEVA website as pSEVA-sib plasmids. Benefits – a large variety of the three standardized part types are available and widespread use of the SEVA plasmids. Drawbacks - part domestication requires re-coding, inefficient cloning methods, limited distribution of plasmids, limited regulators available as cargo, customization of DNA flanking the cargo is not inherent to the system.

Center panel: Modular cloning utilizes Golden Gate to assemble multigene constructs in a hierarchical fashion. First, genetic elements including promoters (P), 5' untranslated regions (U), signal peptides (SP), coding sequences (CDS) and terminators (T) are domesticated through the removal of internal interfering type IIS restriction sites and individually cloned into level 0 destination vectors to generate level 0 modules, each flanked by fusion sites containing BsaI type IIS restriction sites. Level 1 transcriptional units are then combined with end-linker destination vectors and digested with Bpil type IIS restriction enzyme to generate level 2 constructs. End-linker vectors with different type IIS restriction sites can be included at each level 2 cloning step to increase the number of possible level 1 modules included in the final construct. Destination vectors at levels 0, 1, and 2 contain different antibiotic and color- selectable markers for ease of cloning. Parts and assemblies are generated by researchers in-house. Benefits – the ability to customize the genetic parts around the CDS, a variable number of functional elements can be included, efficient combinatorial assembly with Golden Gate Cloning, and entire toolkits are available through Addgene. Drawbacks – requires several cloning steps and intermediate vector construction that are time-consuming and laborious, and part domestication is required.

Bottom panel: The combinatorial assembly strategy described in this work utilizes overlap cloning to generate vectors consisting of four genetic parts wherein shared intergenic regions between each part enable swapping of part variants. Vectors can be assembled in four parts, two halves, or as a backbone of three parts and one insert. Parts can be generated in-house through initial part amplification with primers containing a 3' end that anneals to the part and the conserved sequence on the 5' overhanging end. Plasmids containing the 12 regulator-promoter parts, seven reporter parts, eight marker parts, and five origin parts are available through Addgene. Benefits - ease of vector construction, no constraints for the addition of variant parts.. Drawbacks – customization of components outside of four main parts is not inherent in the system.

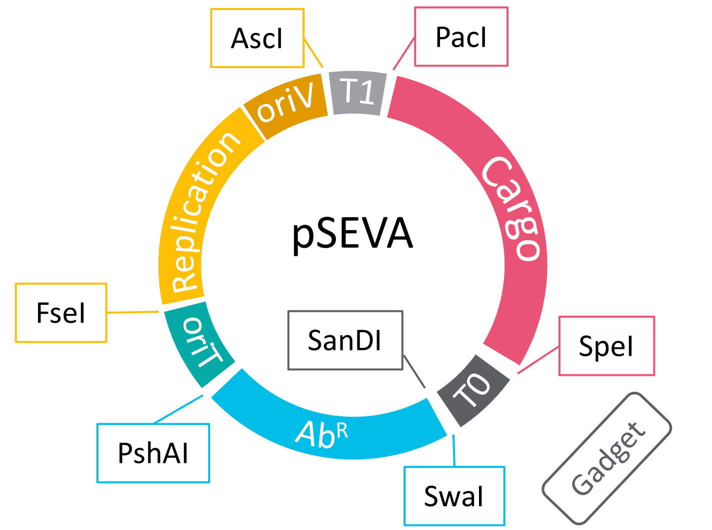

SEVA

- Platform including design concept for standardized vectors and repository of parts
- Assembly via classical restriction enzyme cloning
- Domestication of parts:
  - Removal of interfering restriction enzyme sites
  - Flanking of part with appropriate restriction sites

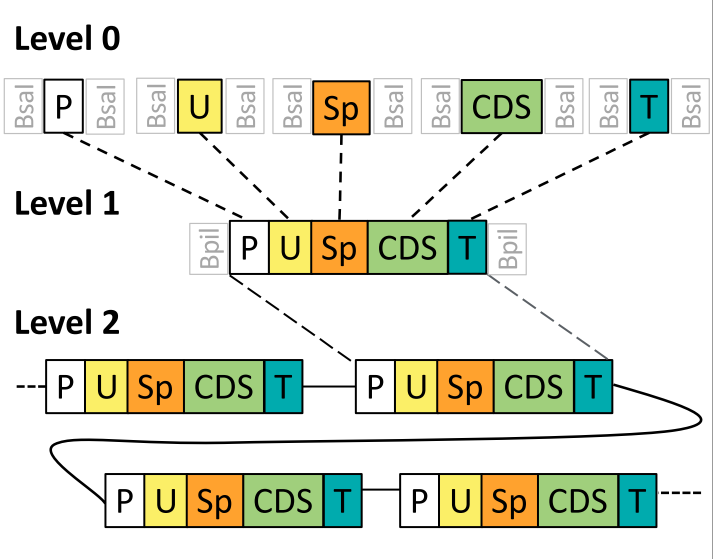

Modular Cloning (MoClo)

- System for modular part assembly in hierarchical fashion
- Assembly via Golden Gate cloning
- Domestication of parts:
  - Removal of interfering type IIS restriction enzyme sites
  - Addition of fusion sites and type IIS recognition site

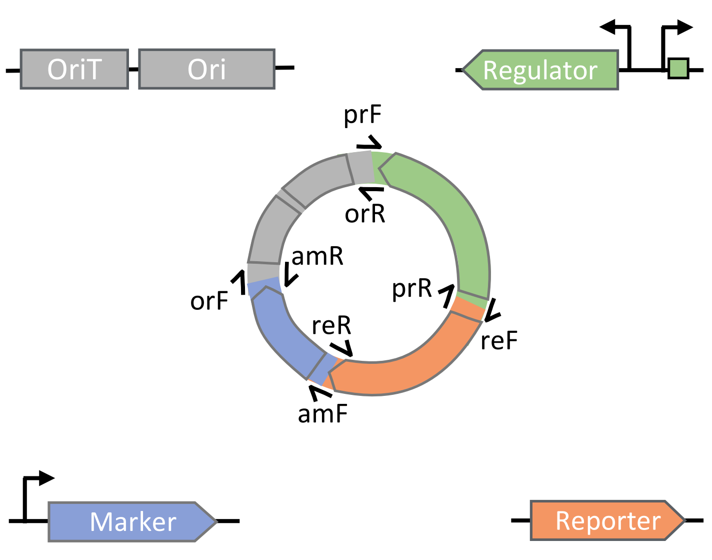

Combinatorial Assembly of Broad Host Range Vectors

- System for modular part assembly in combinatorial fashion
- Assembly via HiFi assembly
- Domestication of parts:
  - Addition of shared intergenic region during initial part amplification

Supplementary Note 2: Plasmid Assembly Protocol and Cost Analysis

1. PCR Amplification of Parts

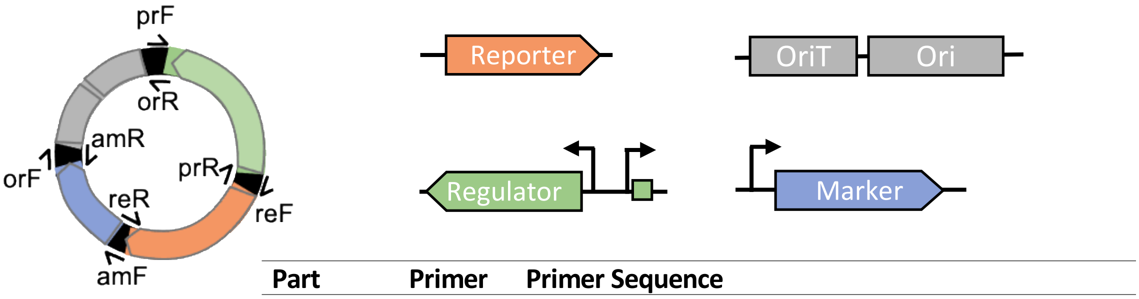

Part	Primer	Primer Sequence
Origin	orF	ggtggcacttttcgggga-------
	orR	tcactggccgtcgttttac-------
Regulator	prF	gtaaaacgacggccagtga-------
	prR	catctagtatttctcctctttaatctgcgctcttcccagttc-------
Reporter	reF	gcagattaaagaggagaaatactag(atg)-------
	reR	caggtttcccgactggaaagc-------
Marker	amF	gctttccagtcgggaaacctg-------
	amR	tccccgaaaagtgccacc-------

The schematic above shows the four primer pair sequences that bind to conserved intergenic regions of the broad host-range plasmids.

- Recombination of existing parts into a new vector

  The four primer pair sequences (underlined in black) are the standard primers that can used to assemble any variation of the plasmid from parts taken from a vector compatible with the system. These primers can be used to amplify one, two, or three consecutive parts to perform two, three, or four part assemblies.

- New part addition

  For each new origin, regulator, reporter/gene of interest, or maker part to be amplified, the corresponding standard primer sequence should be added to the 5'end of a primer with a 3' end that binds to the new part. The reF primer contains a start codon (highlighted in yellow) to demonstrate where the start codon of the new gene should be placed, care should be taken to ensure that this is the only start codon of the gene. The longer length of primer prR is necessary to avoid disruption of the RBS sequence of the reporter/gene of interest.

  After this initial amplification, the part is now compatible with the combinatorial assembly system.

We recommend a 50 µL PCR reaction using NEB's Q5® High-Fidelity 2X Master Mix or other high-fidelity polymerase of choice, following the manufacturer's instructions. DpnI restriction enzyme (New England Biolabs) is added directly to the reaction tube following

thermocycling and incubated for at least 15 minutes to digest the plasmid used as template for the PCR. We found that 0.5 µL of Dpn1 was sufficient for template digestion.

2. **Gel purification**

   After PCR amplification, parts are visualized with a blue-light compatible DNA stain and gel purified using commercial kits with micro-volume elution columns to obtain highly concentrated DNA. The purifications are eluted in 6-11 µL elution buffer and directly used in the assembly reaction.

3. **Vector Assembly**

   We recommend using NEB's NEBuilder® HiFi DNA Assembly Master Mix.
   In our hands, efficient assembly was achieved with a 2 µL total assembly reaction, reducing the cost of each assembly by ten-fold. We found that efficient assembly can be performed without normalizing the molar ratio of DNA parts. We recommend that at least 20 ng of each part is added, though less may be sufficient. We generally mix 0.25 µL of each part when performing a four-part assembly with 1 µL of the HiFi Assembly Master Mix and incubate at 50°C for 1 hour.

4. **Transformation**

   The total volume of the assembly reaction is used to transform chemically competent cells of NEB5α strain of *E. coli*. After one hour of recovery in 1 mL of Super Optimal Broth, 200 µL of the recovery is plated on to LB plates with selection. The remaining 800 µL can be saved for additional plating if no colonies are obtained after overnight incubation.

Cost Analysis for Assembly of 4-part Construct

Reagent	Manufacturer	Product Amount (Number)	Cost per reaction	Total Cost for first assembly	Cost per additional assembly
Primers	Eurofins	40-bp custom oligo (2)	$12	$48	-
Q5® High-Fidelity 2X Master Mix	NEB	2 x 1.25 ml (M0492S)	$1.85	$7.40	-
Dpn1	NEB	1,000 units (R0176S)	$0.67	$2.68	-
QIAquick Gel Purification Kit	Qiagen	250 reactions (28706)	$2.38	$9.54	-
NEBuilder® HiFi DNA Assembly Master Mix	NEB	50 reactions (E2621L) (1 µL per rxn)	-	$1.26	$1.26
		**Total Cost**	$16.9	$68.88	$1.26

Cost analysis for amplification and gel purification of first assembly assumes that each part is used to assemble one plasmid. As only 0.25 µL of each part is sufficient for a four-part assembly, 24-44 plasmids can be assembled from each 6-11 µL part elution. Additional assemblies require only the NEB HiFi Assembly Master Mix.

Supplementary Note 3: OD in Titrated Inducer Concentrations
Colored floating bars show the range of expression in RFU for 5 concentrations of inducer. All systems with the exception of RhaS-RhaR/P_{RhaBAD} were induced with standard inducer concentration, 10-fold and 100-fold higher than standard concentration, and 10-fold and 100-fold lower than standard concentration. RhaS-RhaR/P_{RhaBAD} was induced with standard inducer concentration, 10-fold higher than standard inducer concentration, and 10-fold, 100-fold, and 1,000-fold lower than standard concentration. Gray dots represent the corresponding optical density (660 nm) at each inducer concentration. Standard error bars shown for optical density measurements. All data is the average of three replicates. Data is shown for measurements taken during exponential phase and late stationary phase for each of the bacteria screened. Fluorescence data from induced cultures with lower RFU than those grown in the absence of inducer due to growth defects are omitted from the graphs.
*Naringenin precipitates out of solution and skews OD readings above 10 mM, data not shown.

A. fabrum

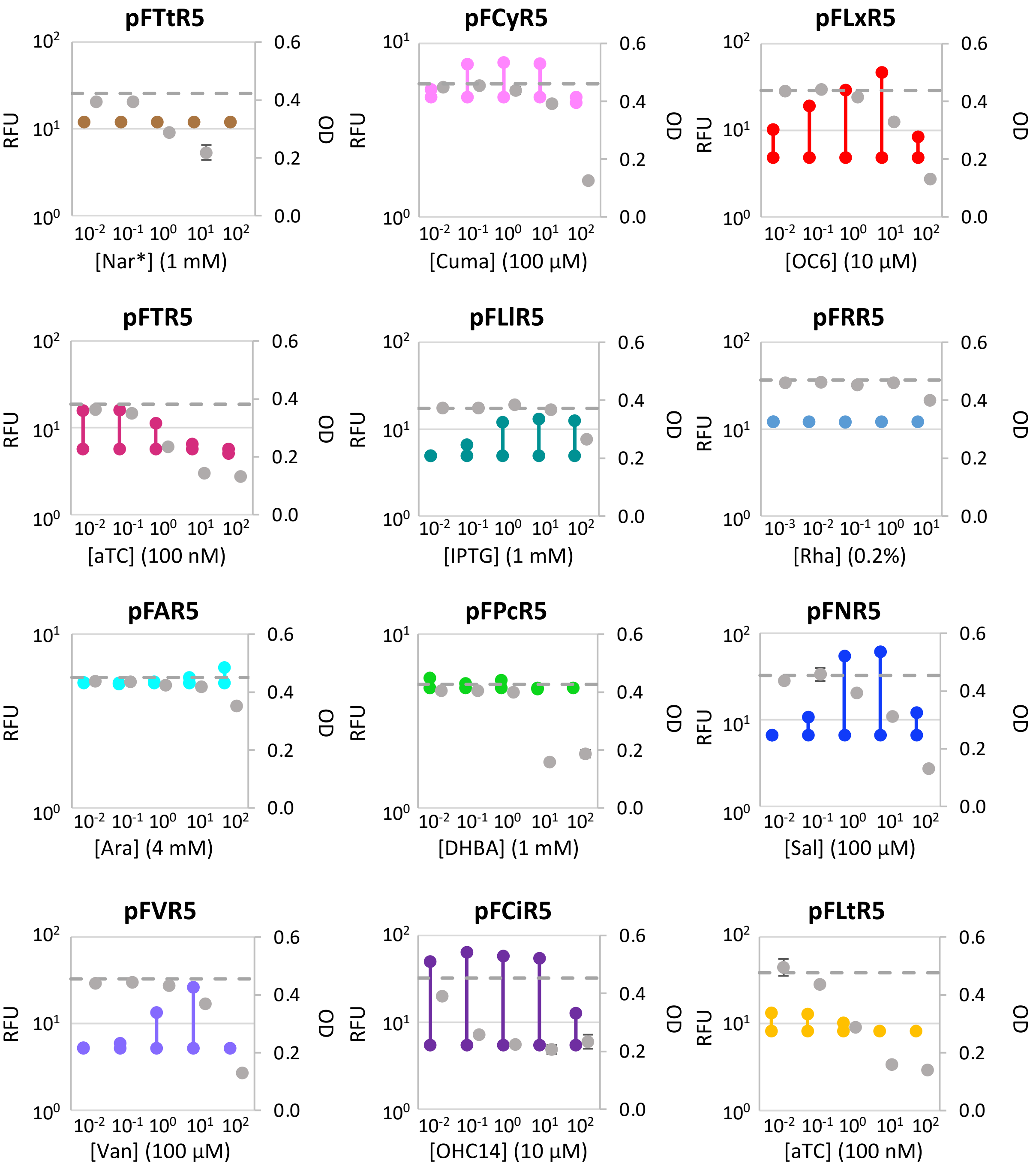

A. fabrum in Exponential Phase.

A. fabrum

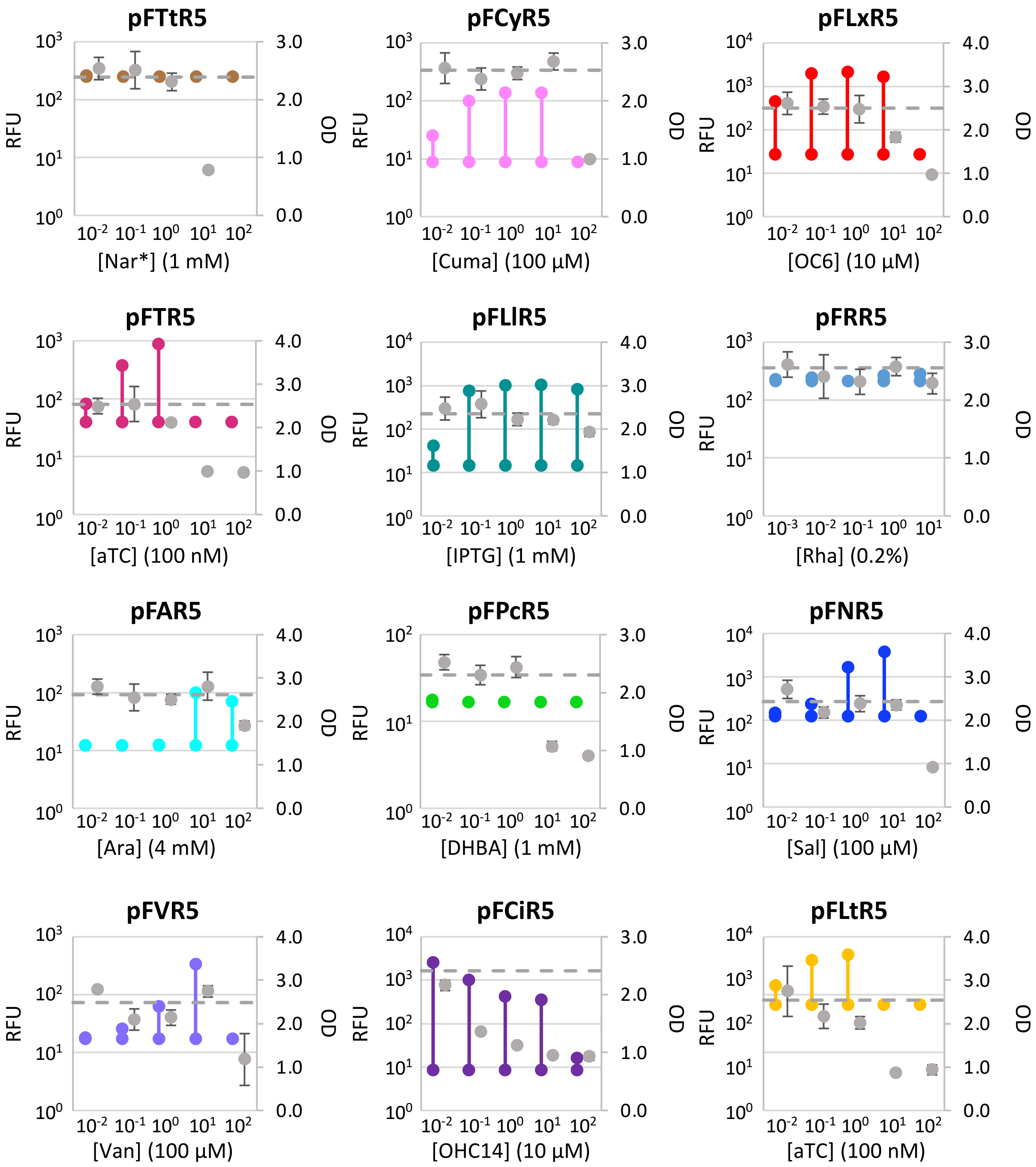

A. fabrum in Stationary Phase.

P. putida

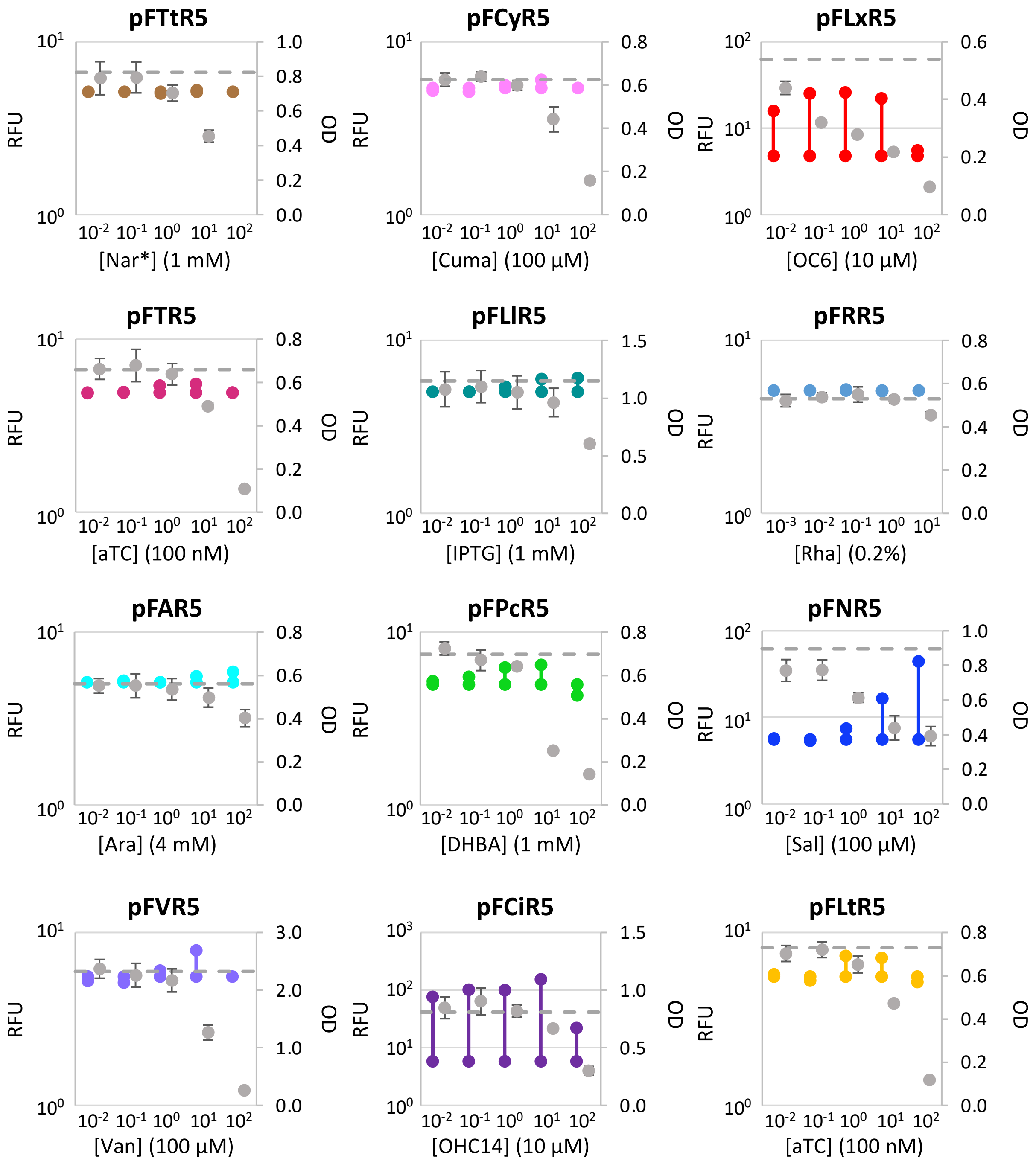

P. putida in Exponential Phase.

P. putida

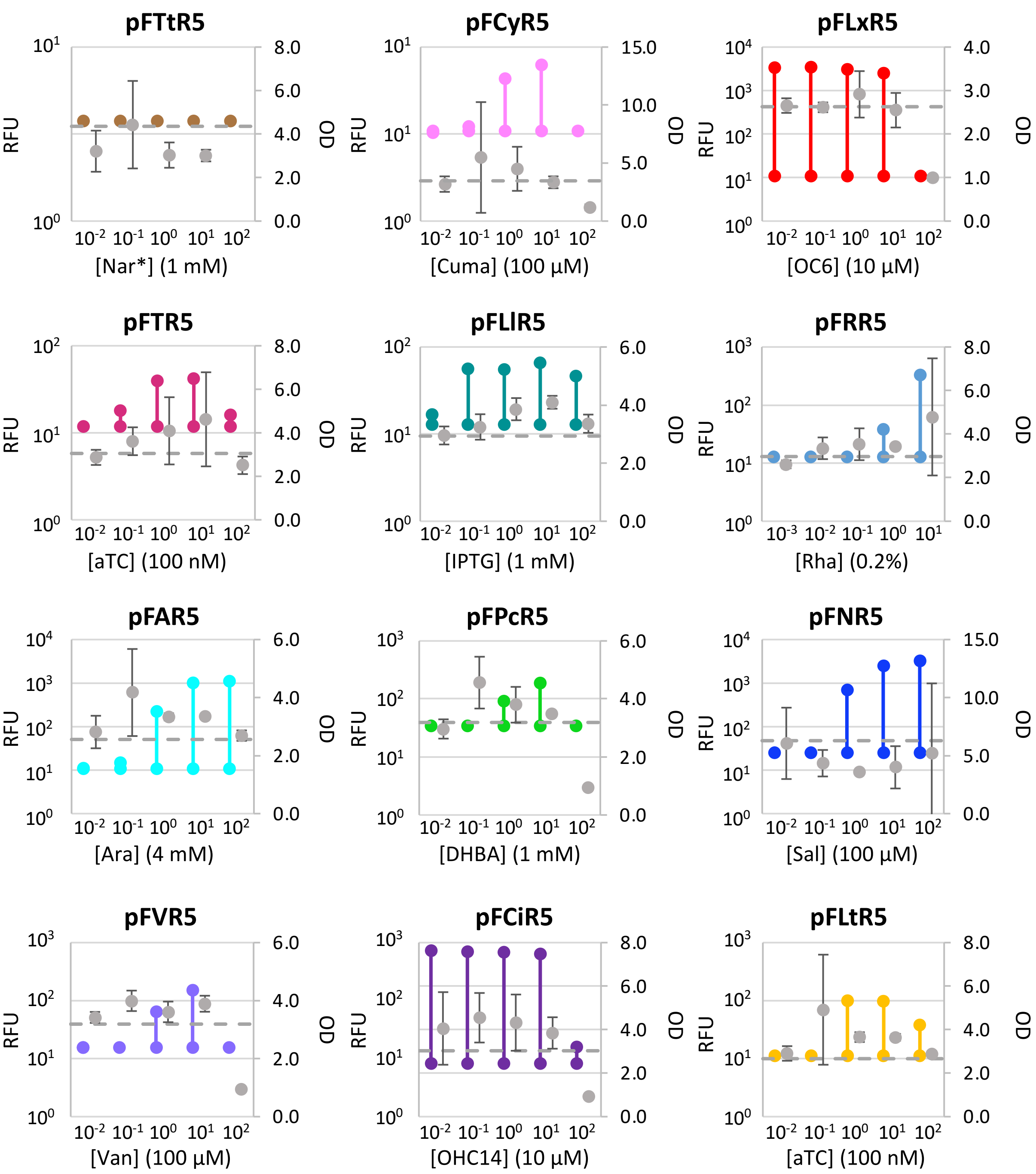

P. putida in Stationary Phase.

X. campestris

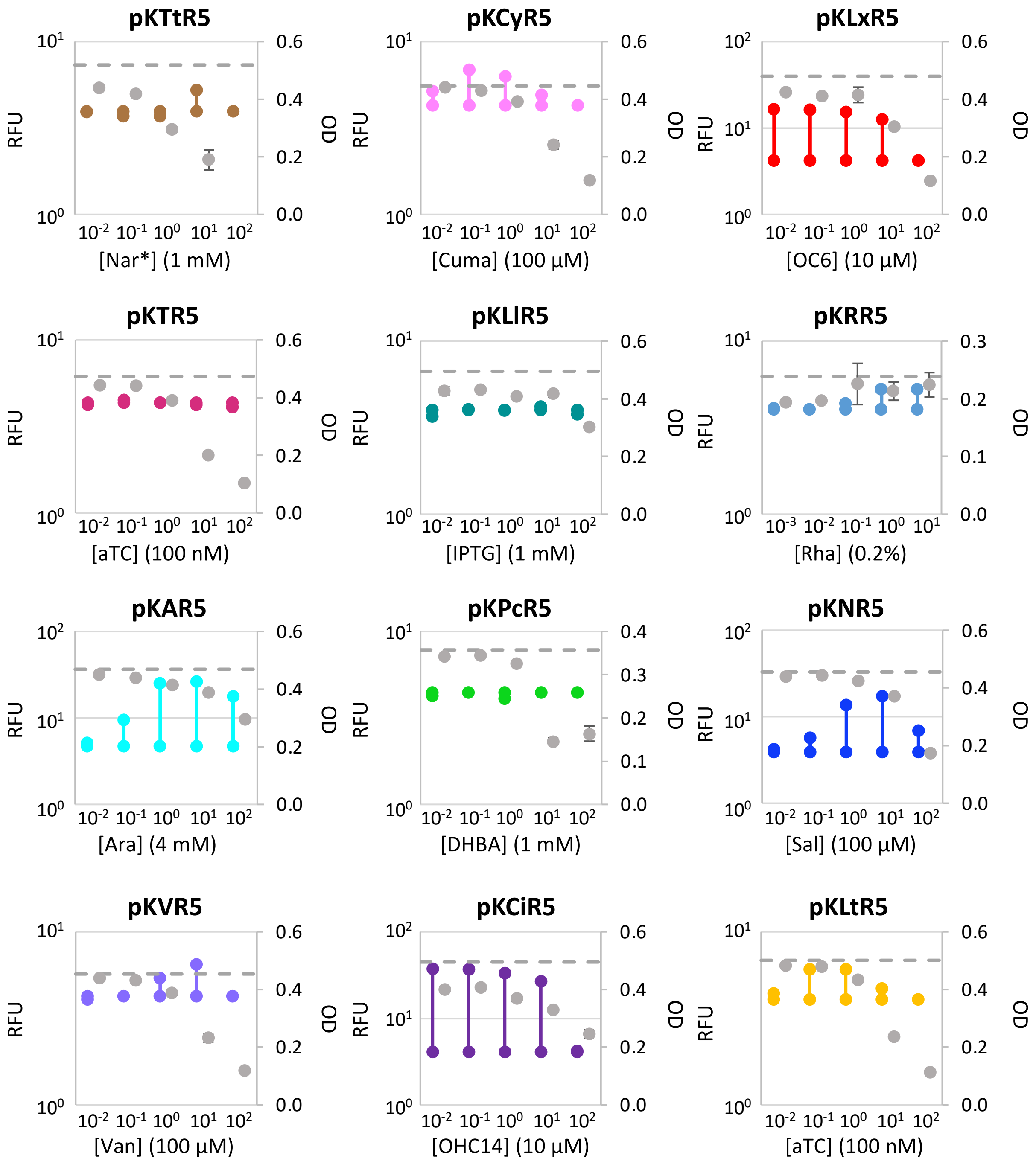

X. campestris in Exponential Phase.

X. campestris

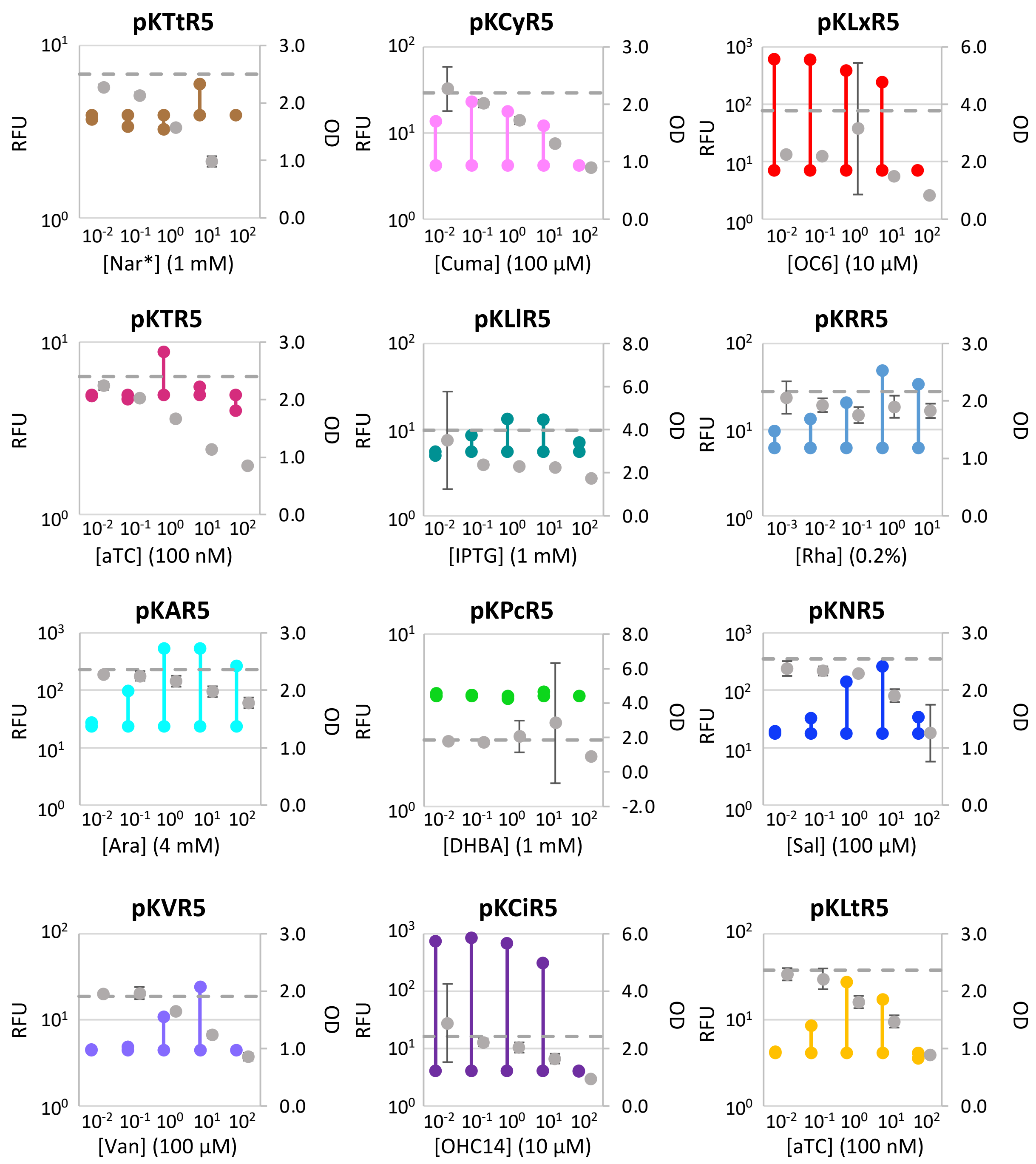

X. campestris in Stationary Phase.

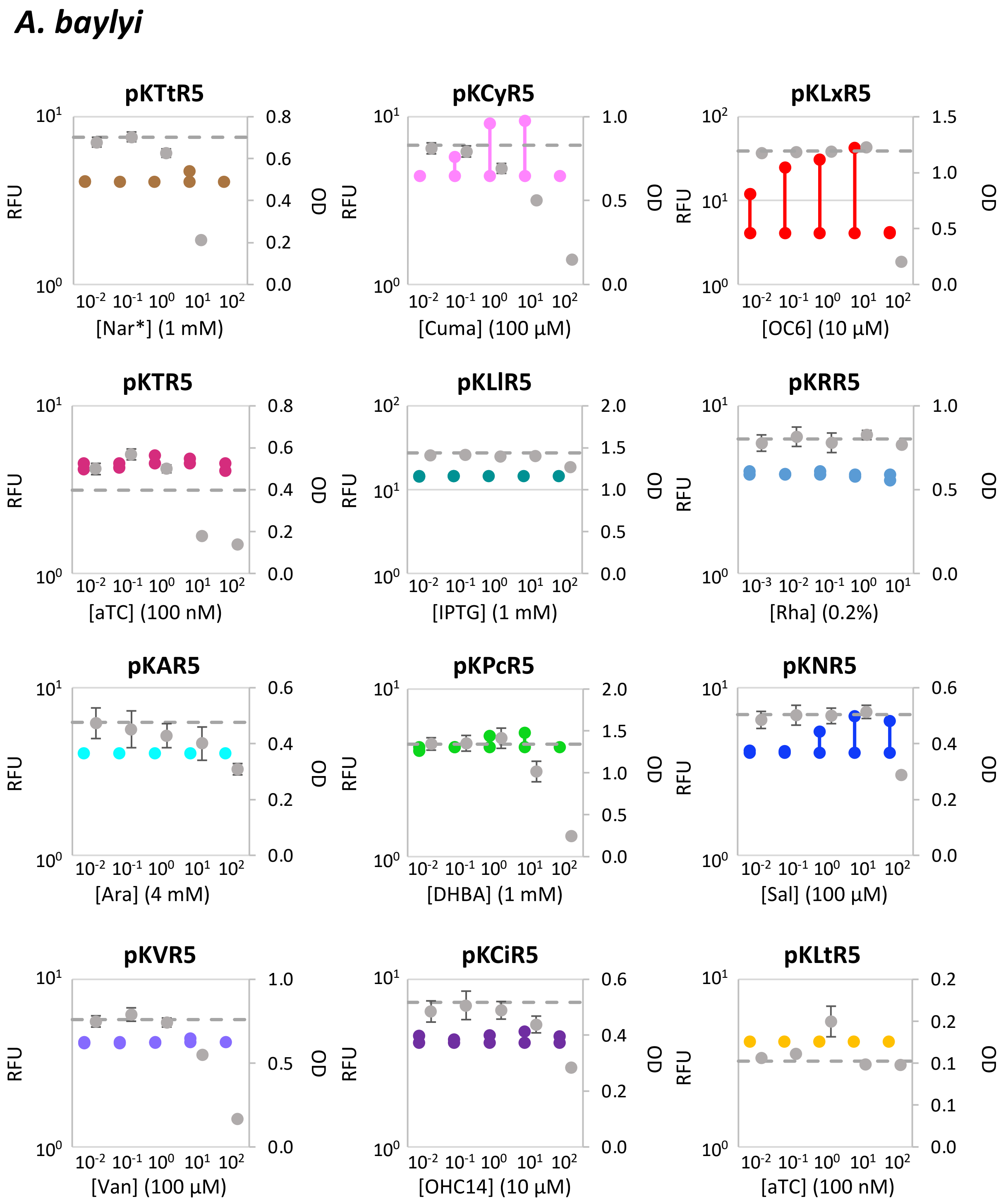

A. baylyi in Exponential Phase.

A. baylyi

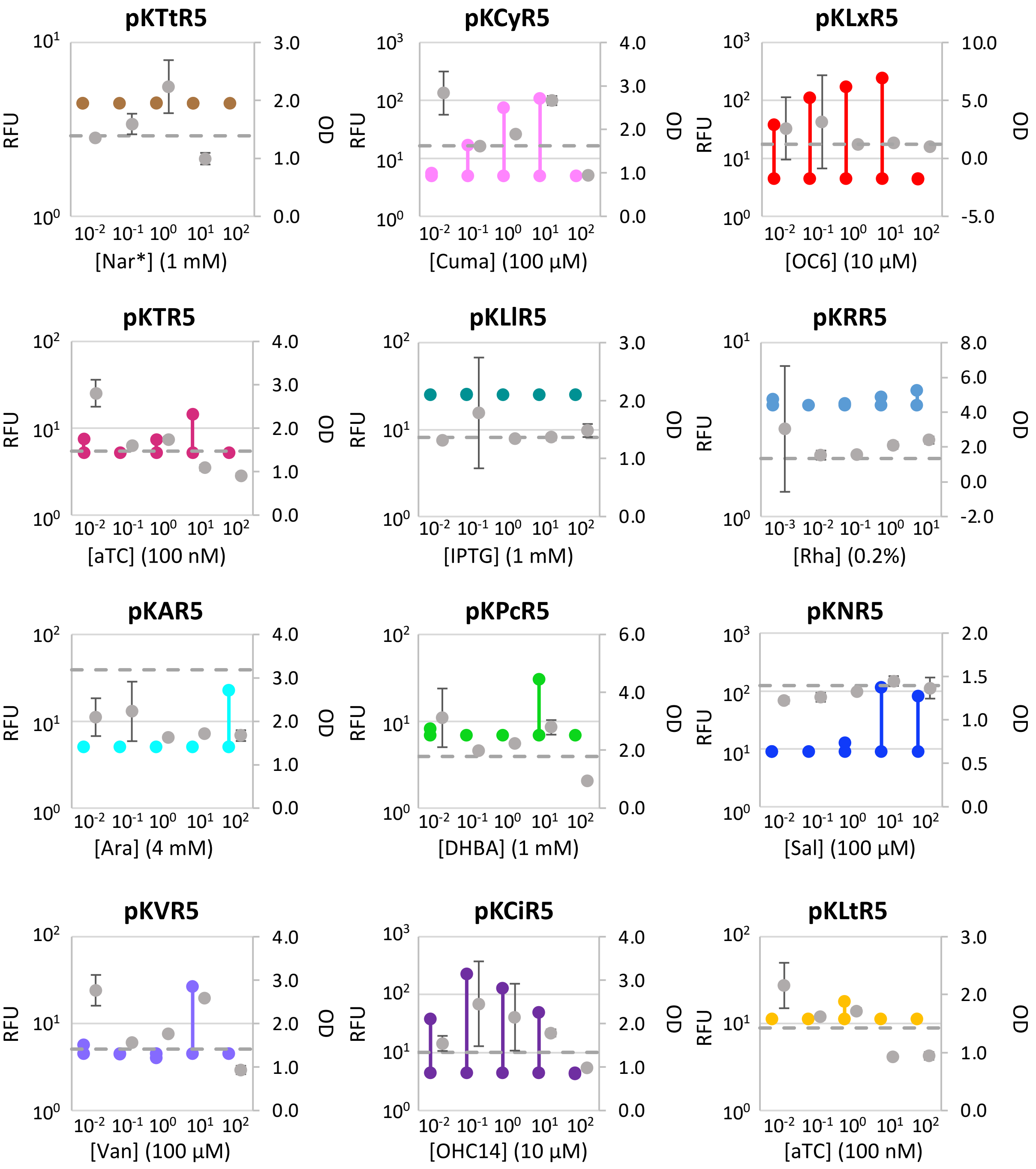

A. baylyi in Stationary Phase.

P. aeruginosa

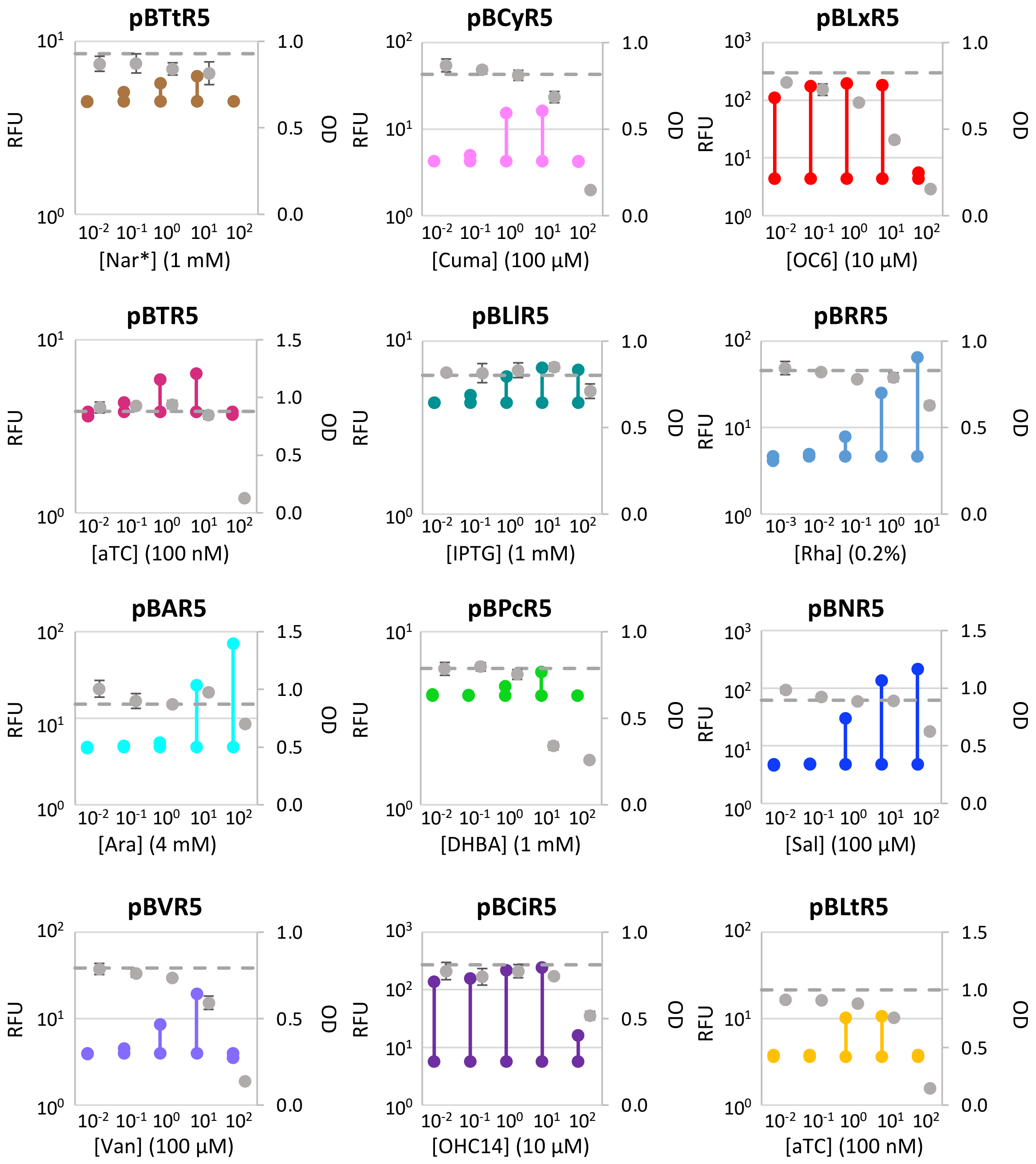

P. aeruginosa in Exponential Phase.

P. aeruginosa

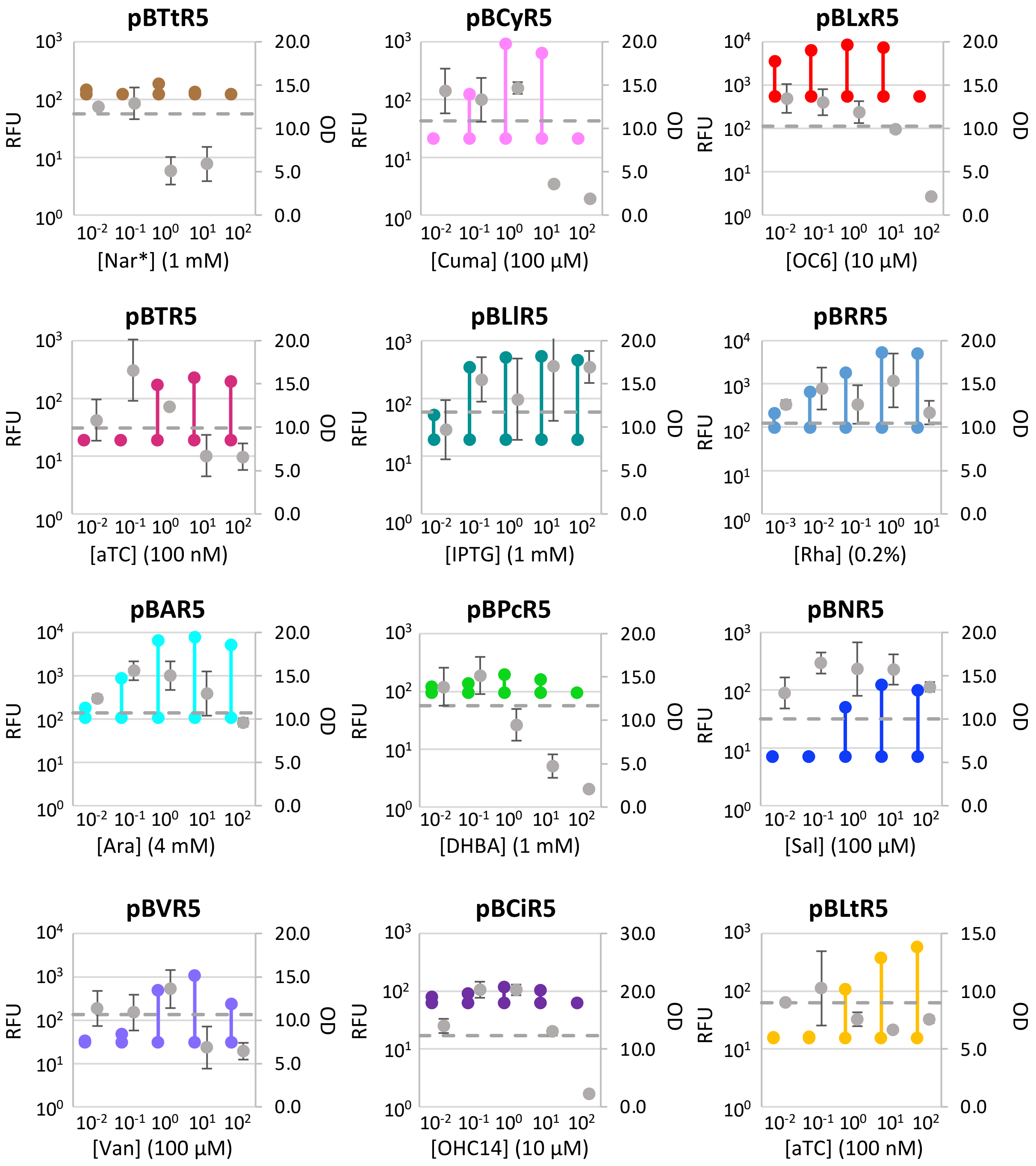

P. aeruginosa in Stationary Phase.

B. thailandensis

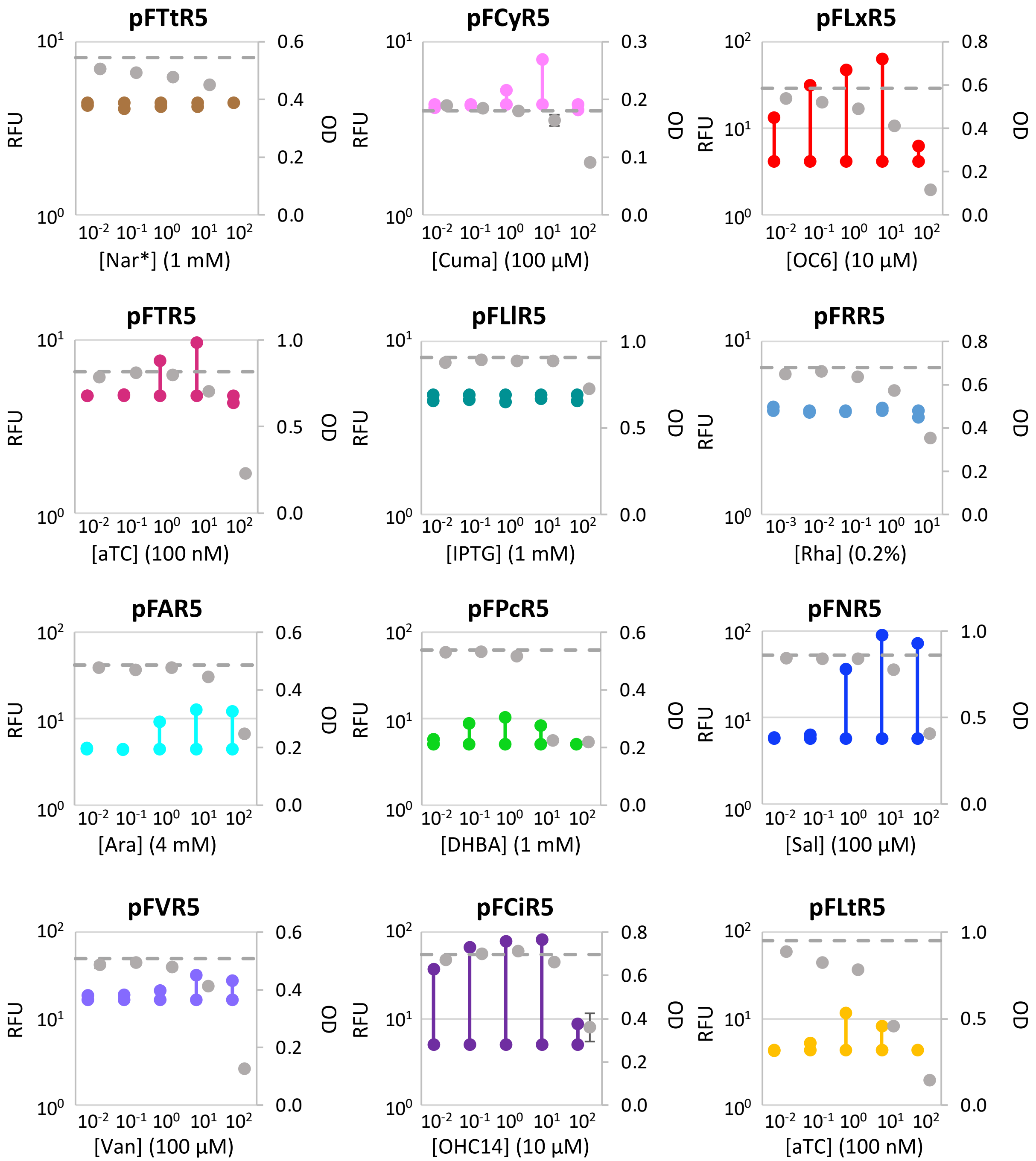

B. thailandensis in Exponential Phase.

B. thailandensis

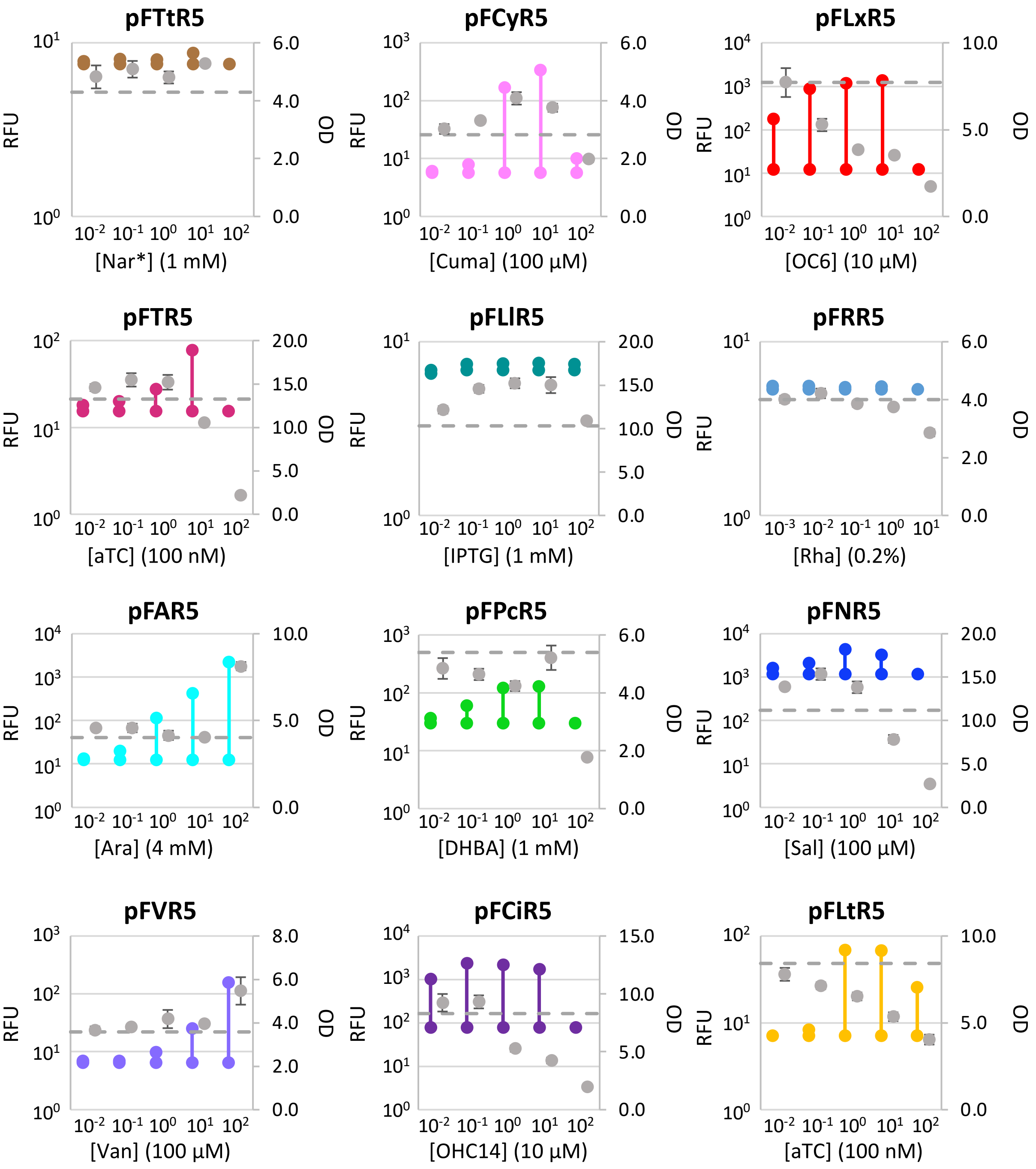

B. thailandensis in Stationary Phase.

Sulfitobacter sp. EE-36

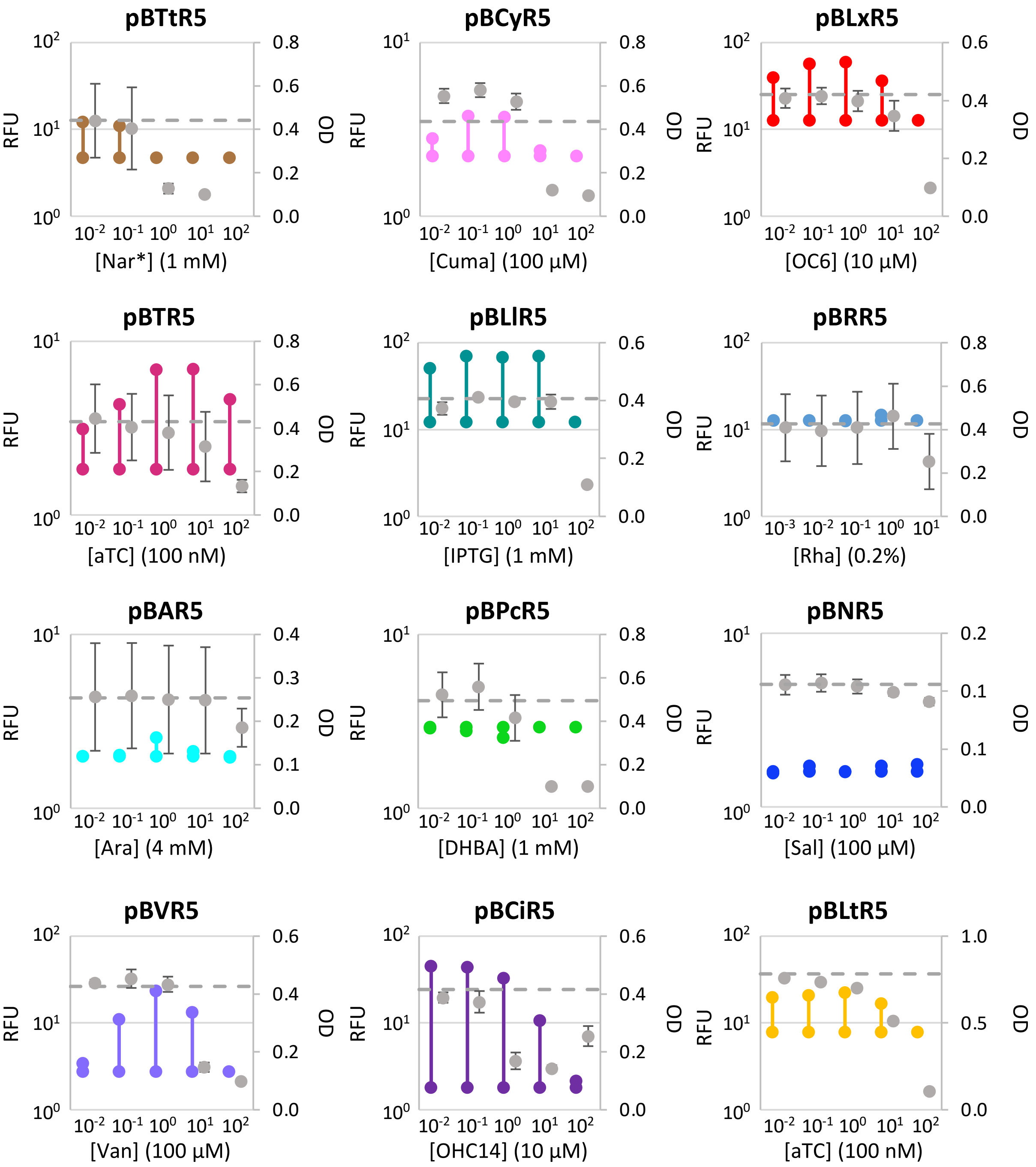

Sulfitobacter sp. EE-36 in Exponential Phase.

Sulfitobacter sp. EE-36

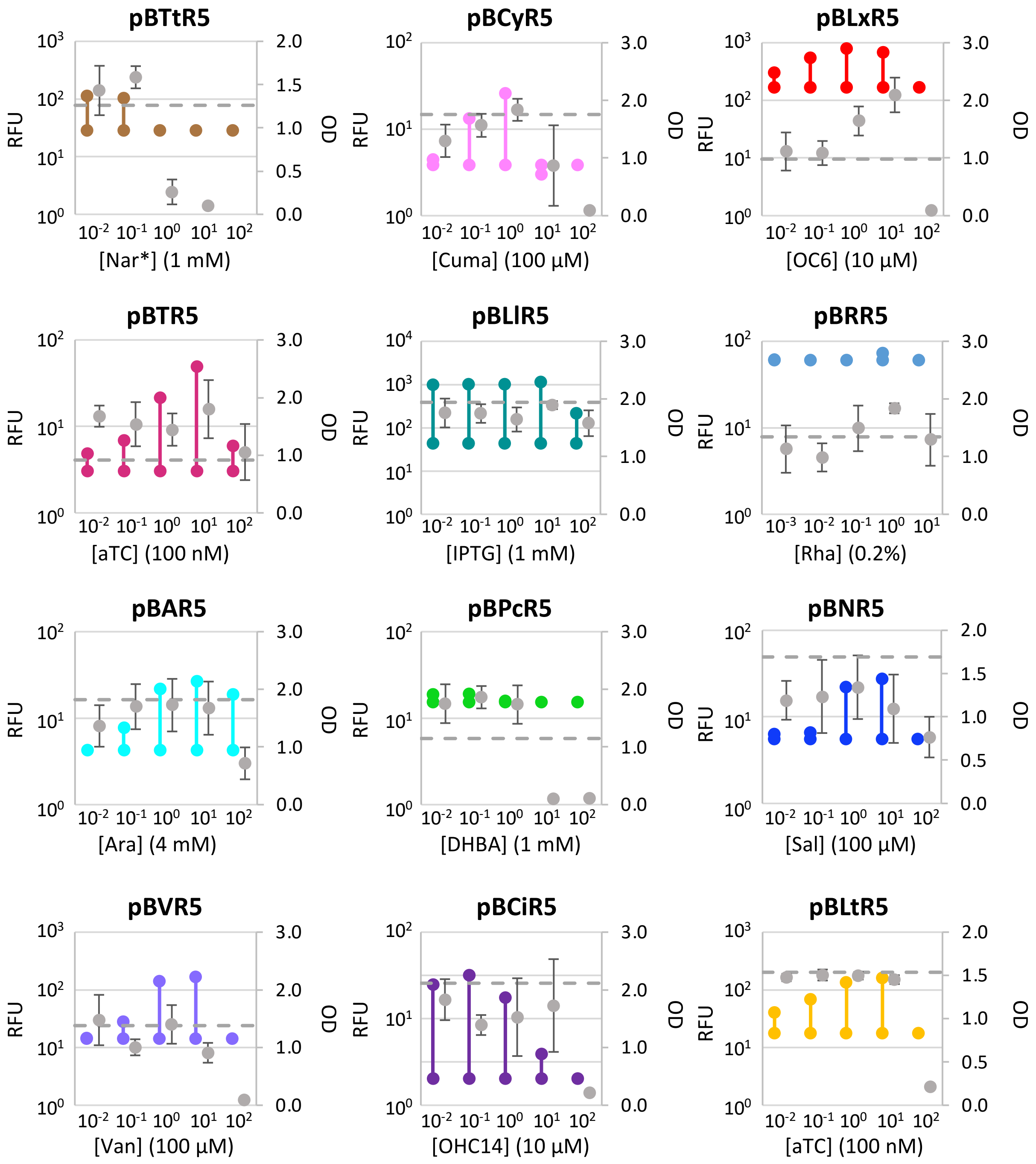

Sulfitobacter sp. EE-36 in Stationary Phase.

***Ruegeria* sp. TM1040**

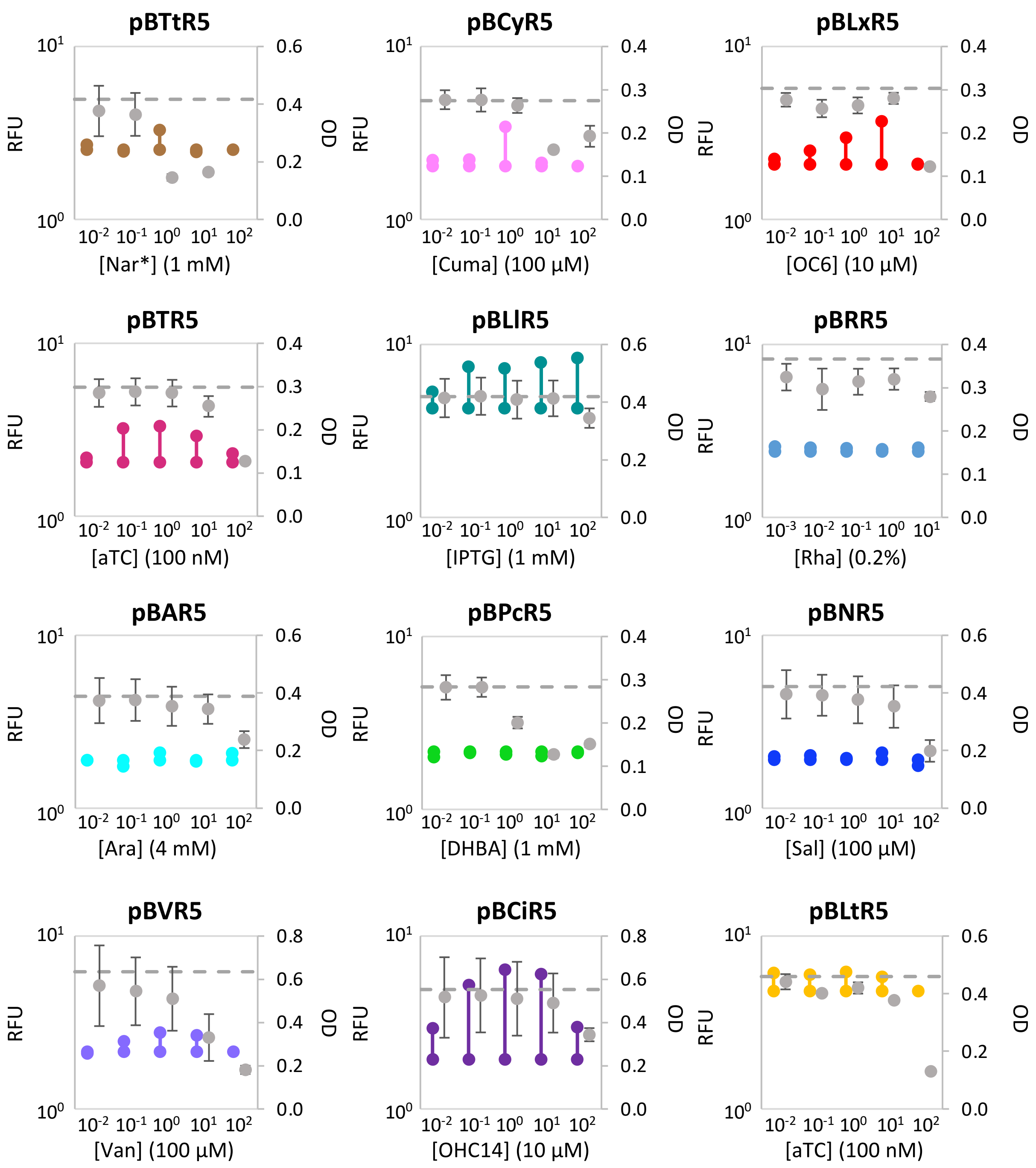

Ruegeria sp. TM1040 in Exponential Phase.

Ruegeria sp. TM1040

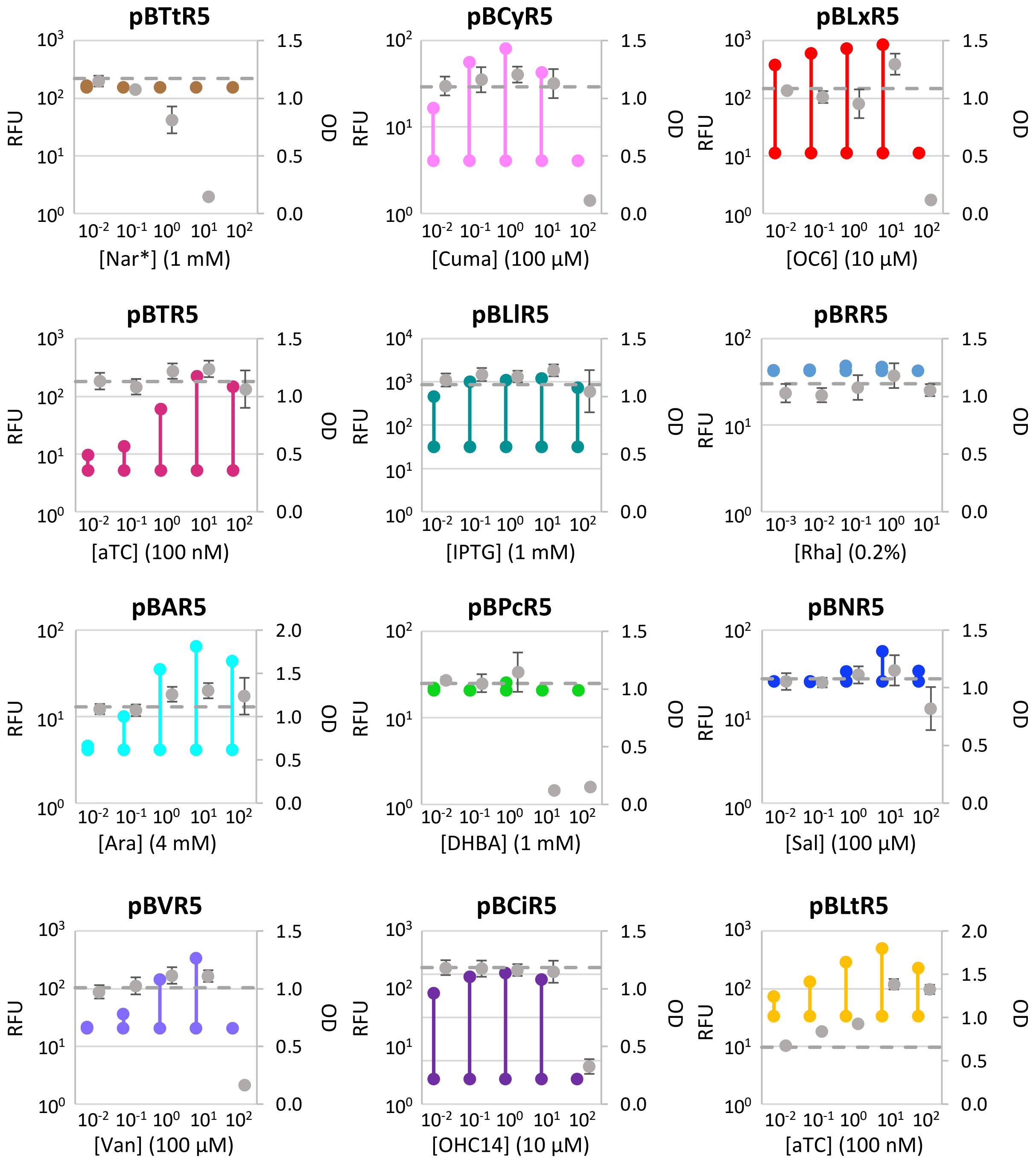

Ruegeria sp. TM1040 in Stationary Phase.

A. fischeri

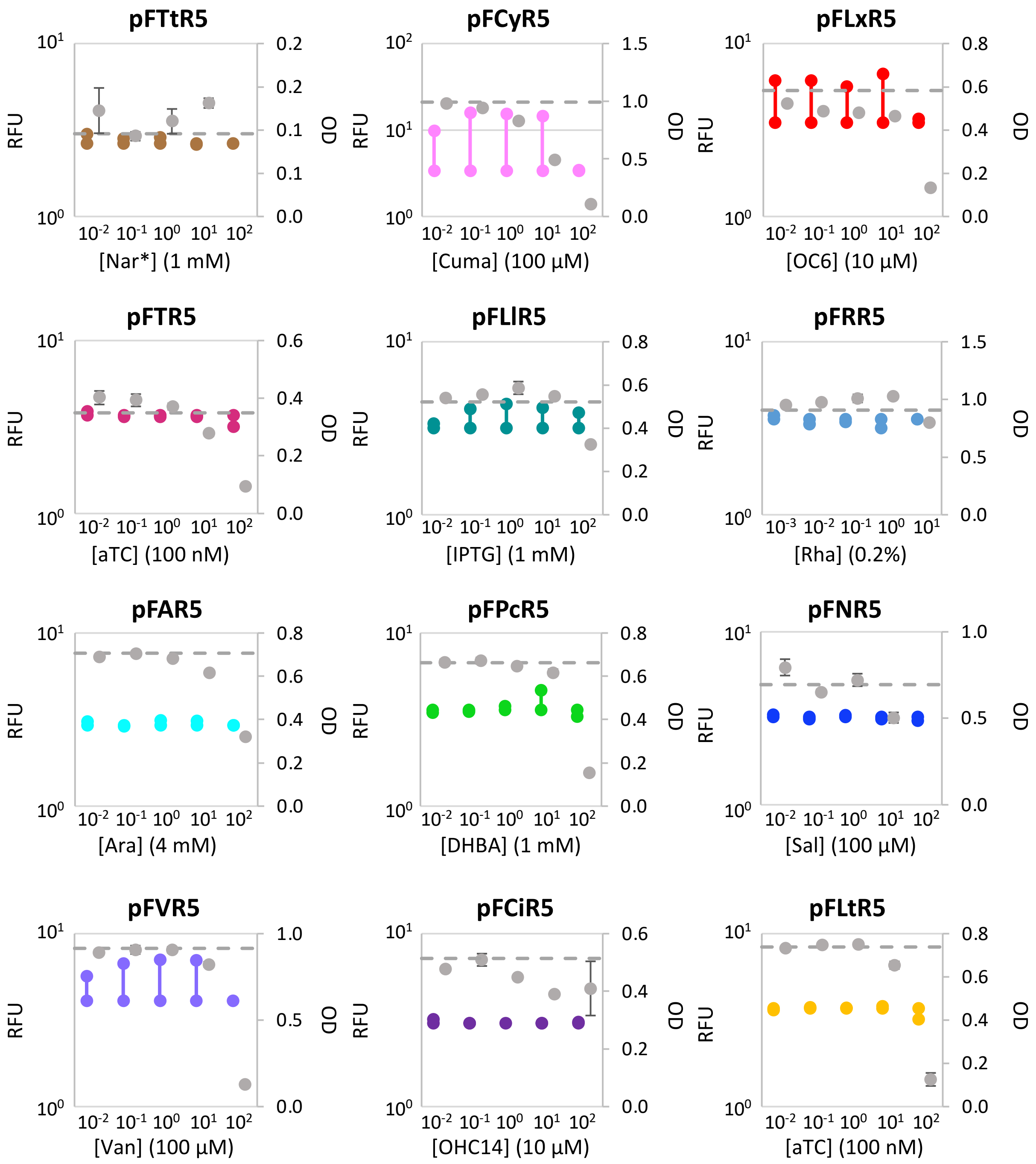

A. fischeri in Exponential Phase.

A. fischeri

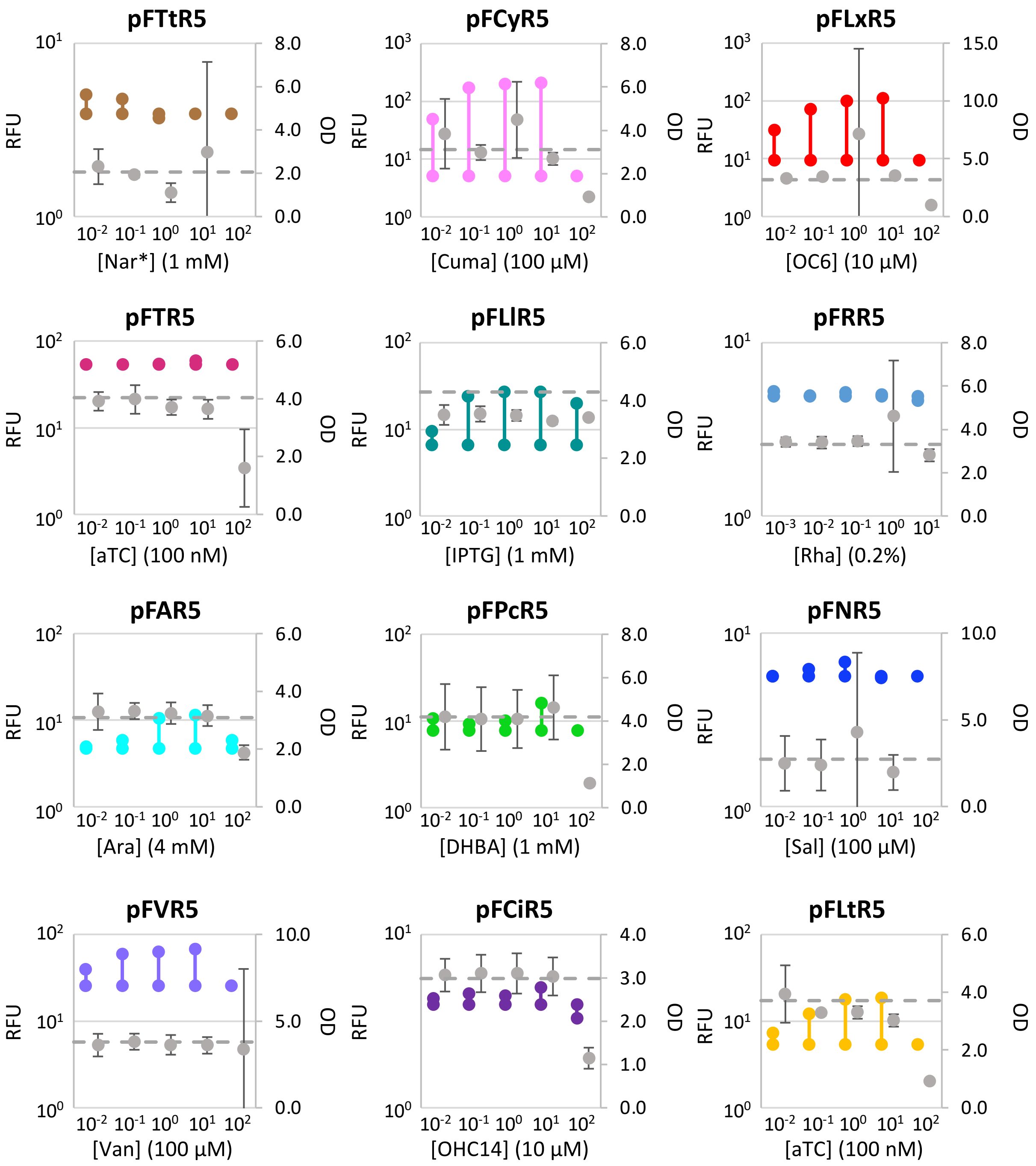

A. fischeri in Stationary Phase.

Supplementary Note 4: RFU Output Over Titrated Inducer Concentrations
Strains containing each of 12 inducible systems were screened in the presence of inducer titrated at five concentrations. All systems except for RhaS-RhaR/P_{RhaBAD} were induced with standard inducer concentration, 10-fold and 100-fold higher than standard concentration, and 10-fold and 100-fold lower than standard concentration. RhaS-RhaR/P_{RhaBAD} was induced with standard inducer concentration, 10-fold higher than standard inducer concentration, and 10-fold, 100-fold, and 1,000-fold lower than standard concentration. Data from cultures grown in the absence of inducer are included on each graph labeled “U”. Fluorescence data from TtgR/P_{Ttg} induced with 100x inducer concentration is not shown due to skewed RFU readings. All data is displayed as RFU normalized to OD_{660}. Closed circles and open squares on the graphs represent data points from the mid-log and late stationary phase of growth. All data is the average of triplicates.

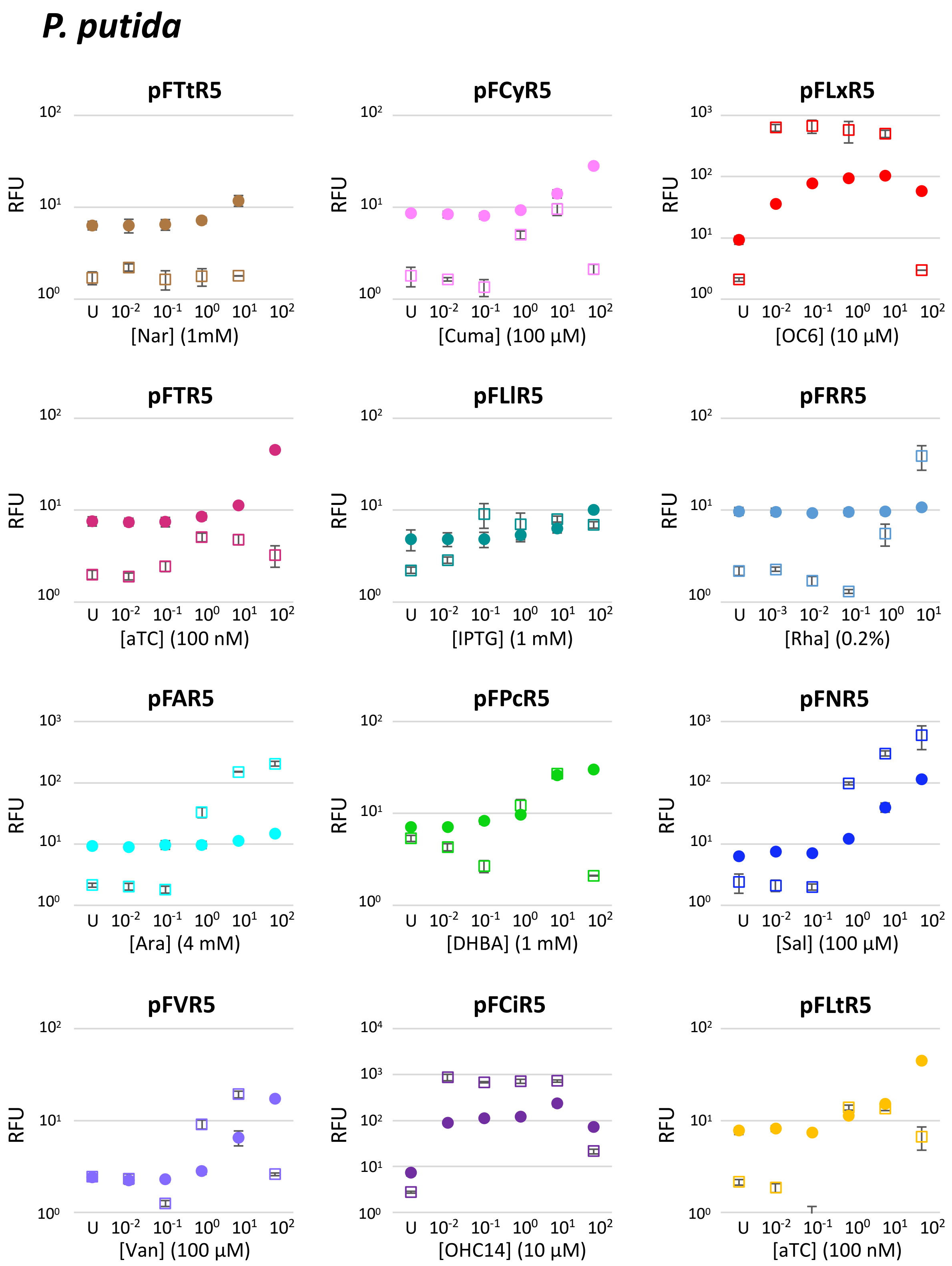
P. putida
pFTtR5
[Nar] (1mM)
pFCyR5
[Cuma] (100 µM)
pFLxR5
[OC6] (10 µM)
pFTR5
[aTC] (100 nM)
pFLlR5
[IPTG] (1 mM)
pFRR5
[Rha] (0.2%)
pFAR5
[Ara] (4 mM)
pFPcR5
[DHBA] (1 mM)
pFNR5
[Sal] (100 µM)
pFVR5
[Van] (100 µM)
pFCiR5
[OHC14] (10 µM)
pFLtR5
[aTC] (100 nM)
RFU
U
P. putida

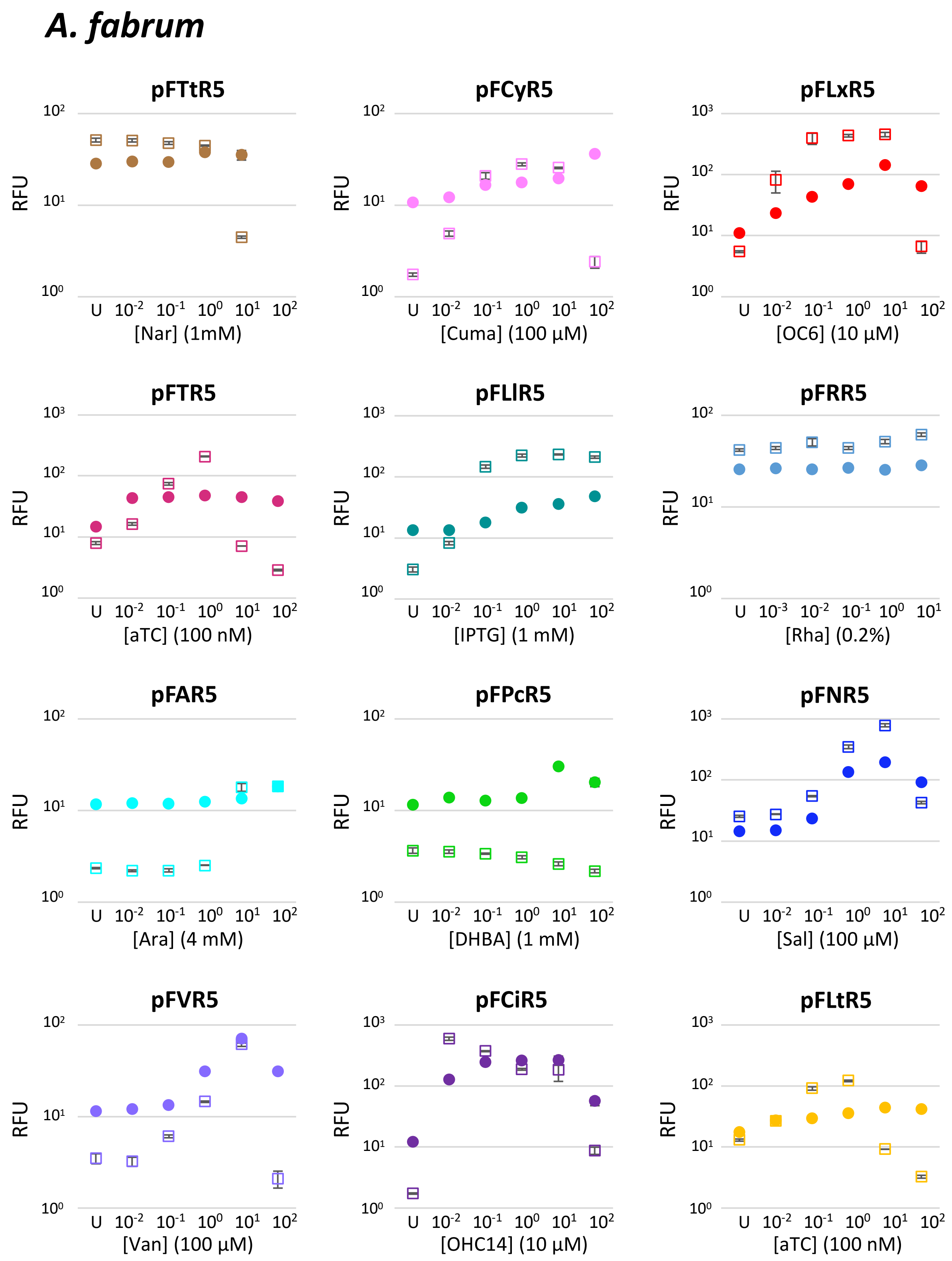
A. fabrum
pFTtR5
[Nar] (1mM)
pFCyR5
[Cuma] (100 µM)
pFLxR5
[OC6] (10 µM)
pFTR5
[aTC] (100 nM)
pFLlR5
[IPTG] (1 mM)
pFRR5
[Rha] (0.2%)
pFAR5
[Ara] (4 mM)
pFPcR5
[DHBA] (1 mM)
pFNR5
[Sal] (100 µM)
pFVR5
[Van] (100 µM)
pFCiR5
[OHC14] (10 µM)
pFLtR5
[aTC] (100 nM)
RFU
U

A. fabrum

A. baylyi

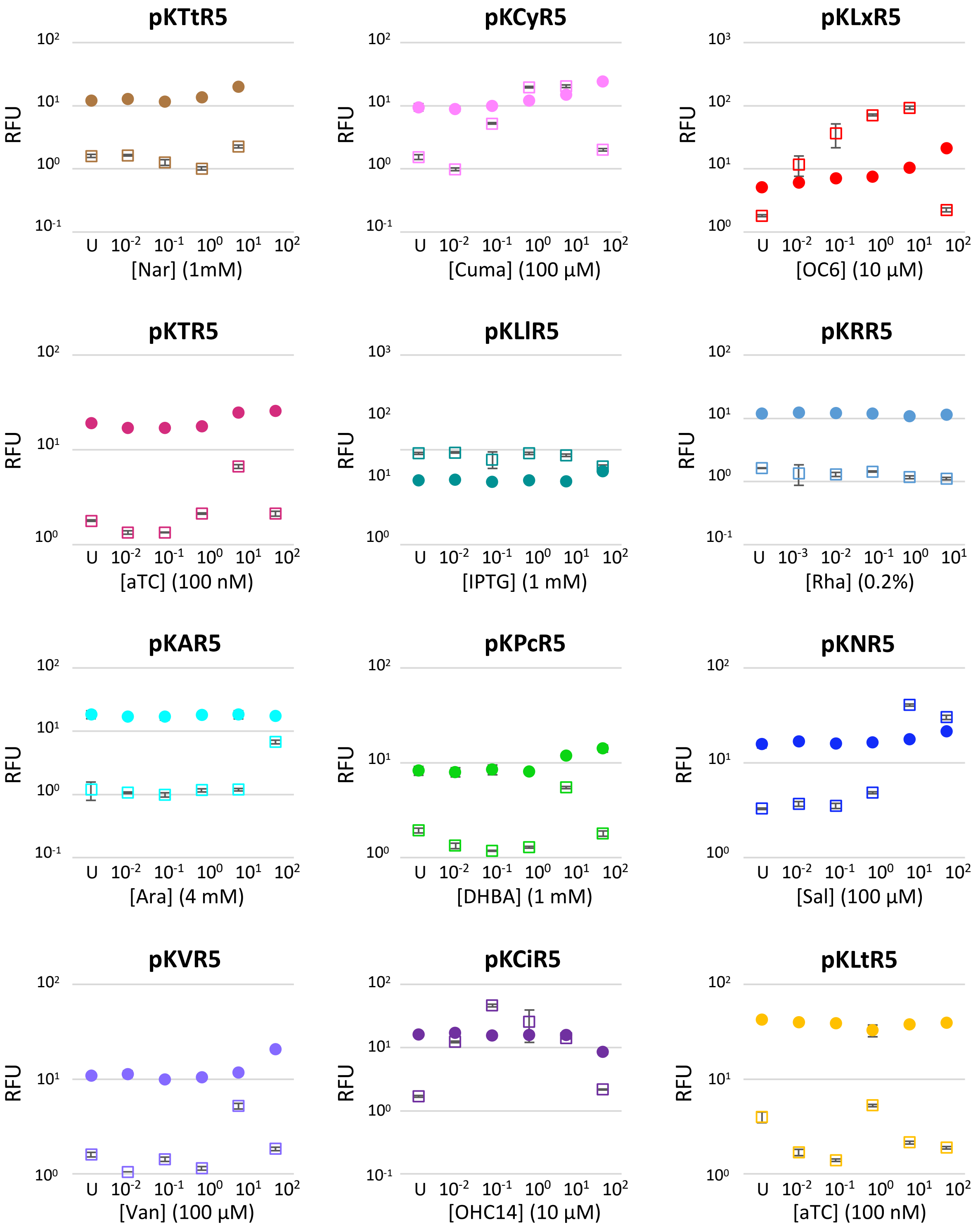

A. baylyi

X. campestris

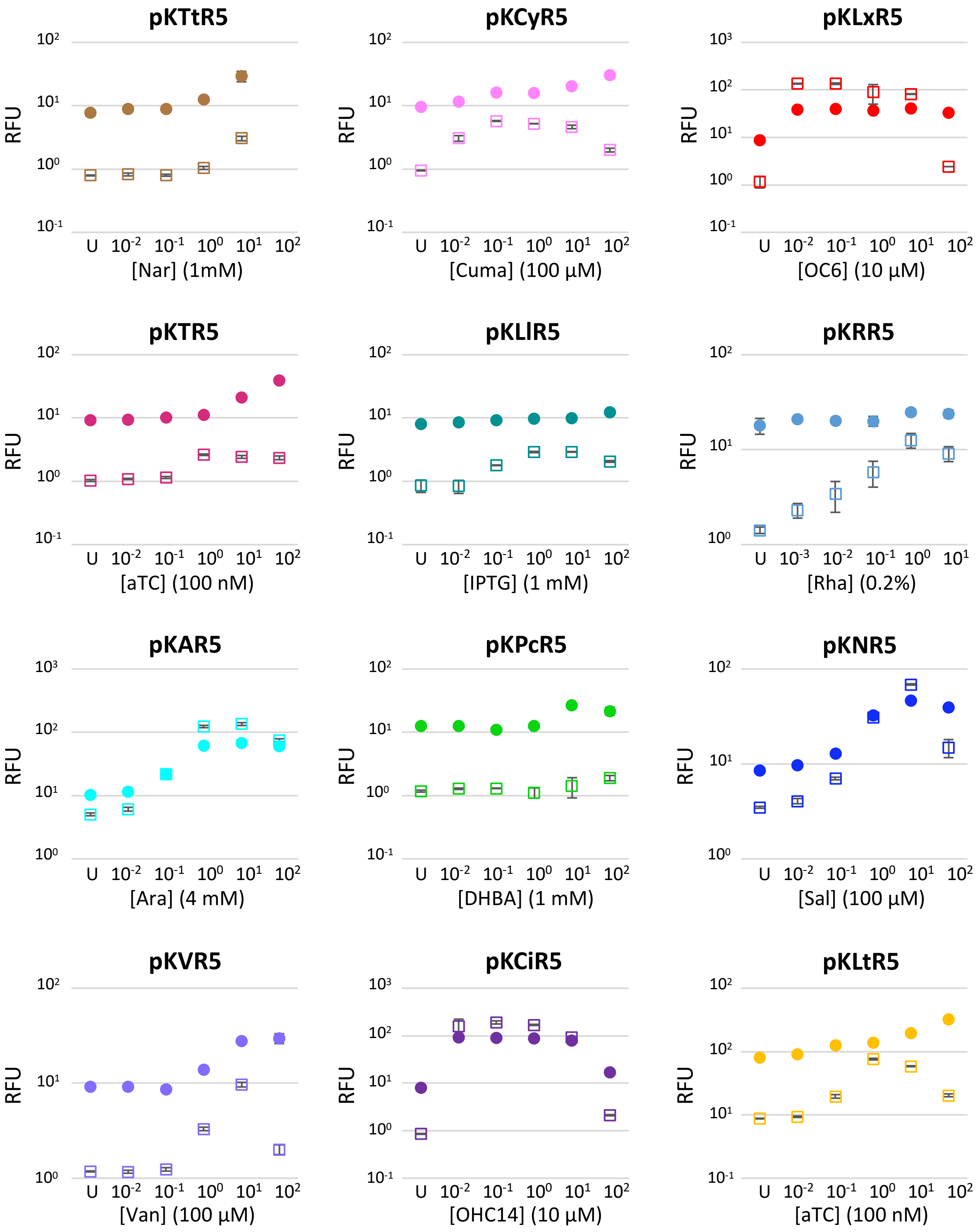

X. campestris

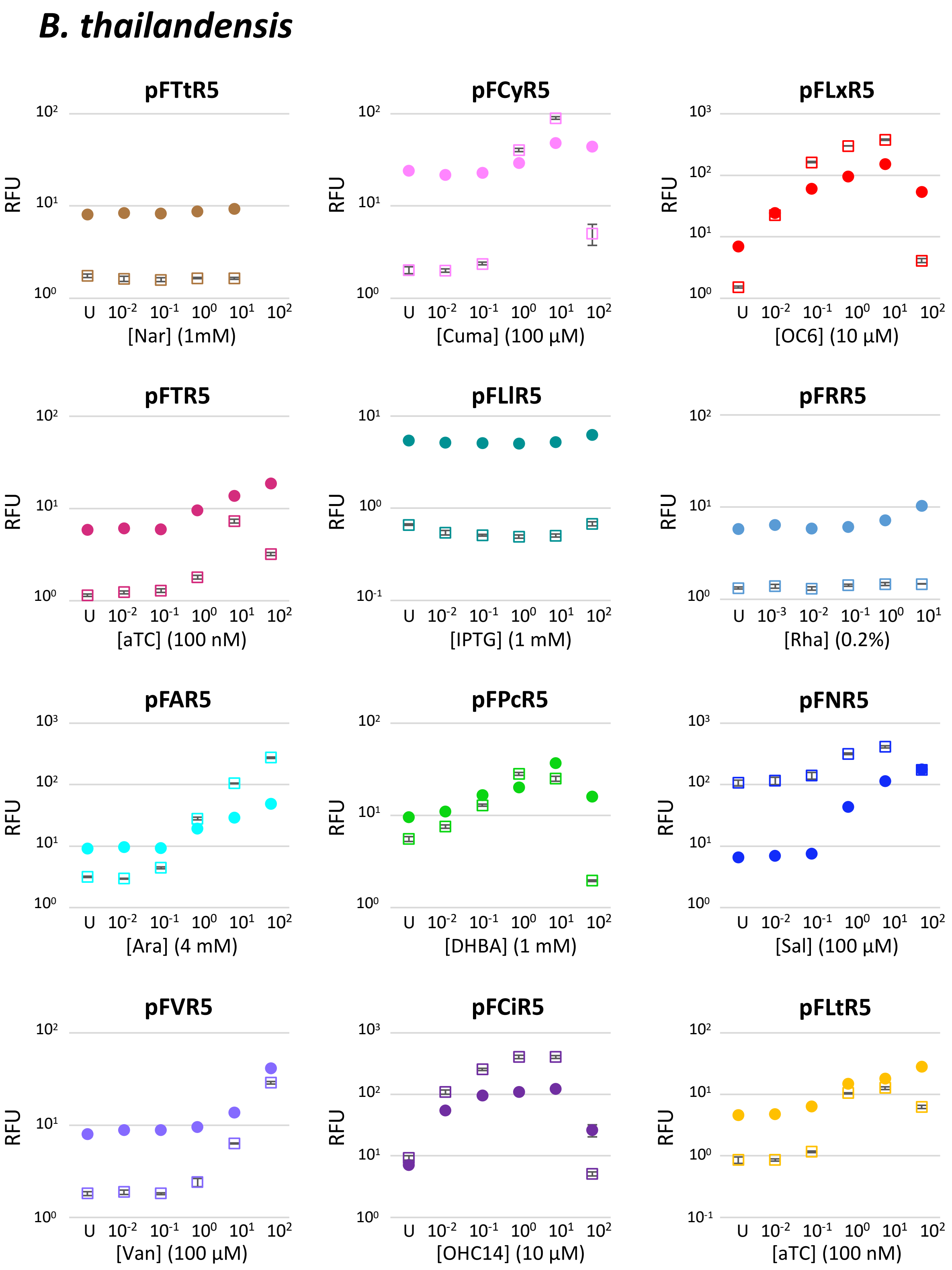

B. thailandensis

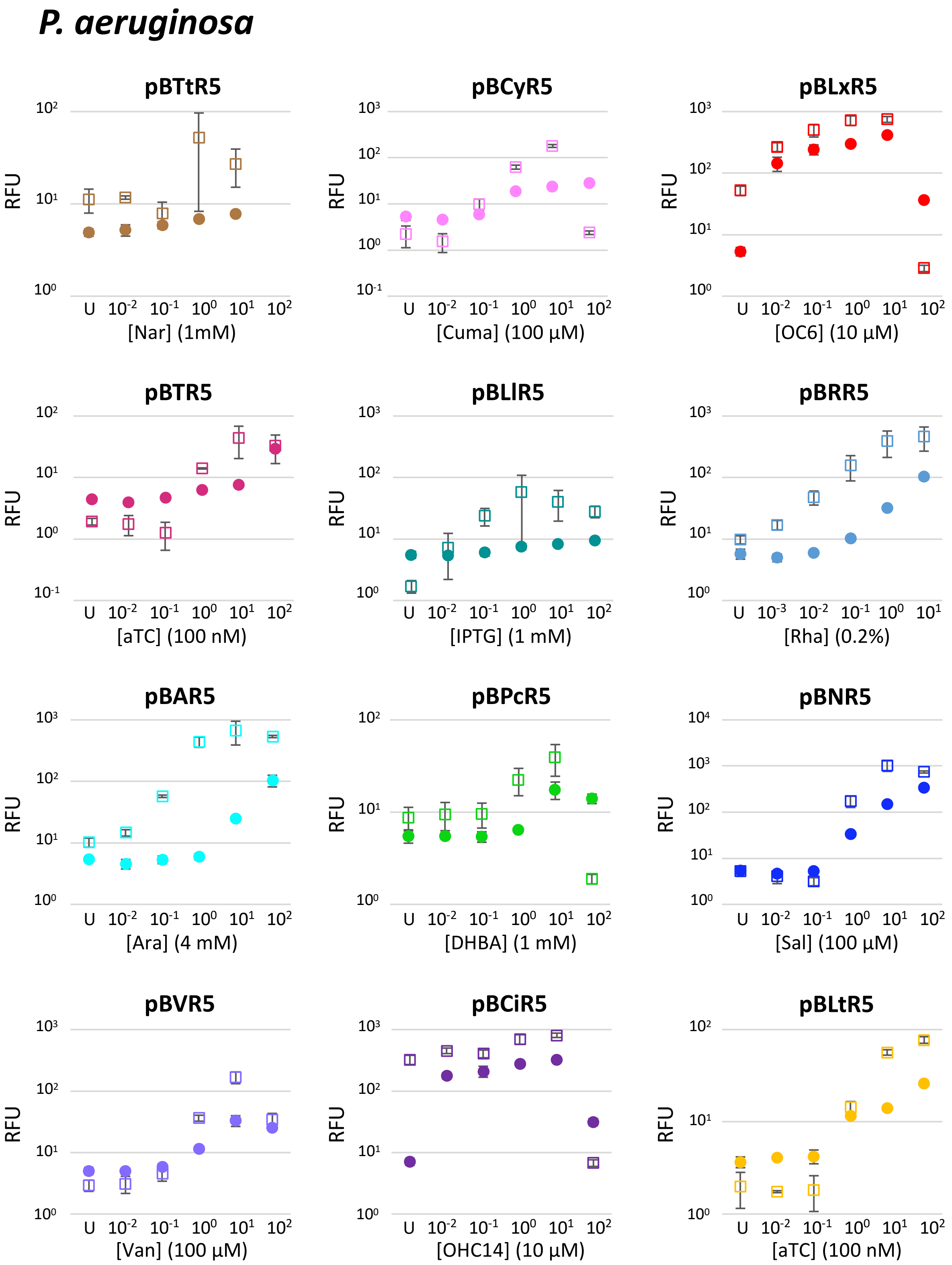

P. aeruginosa

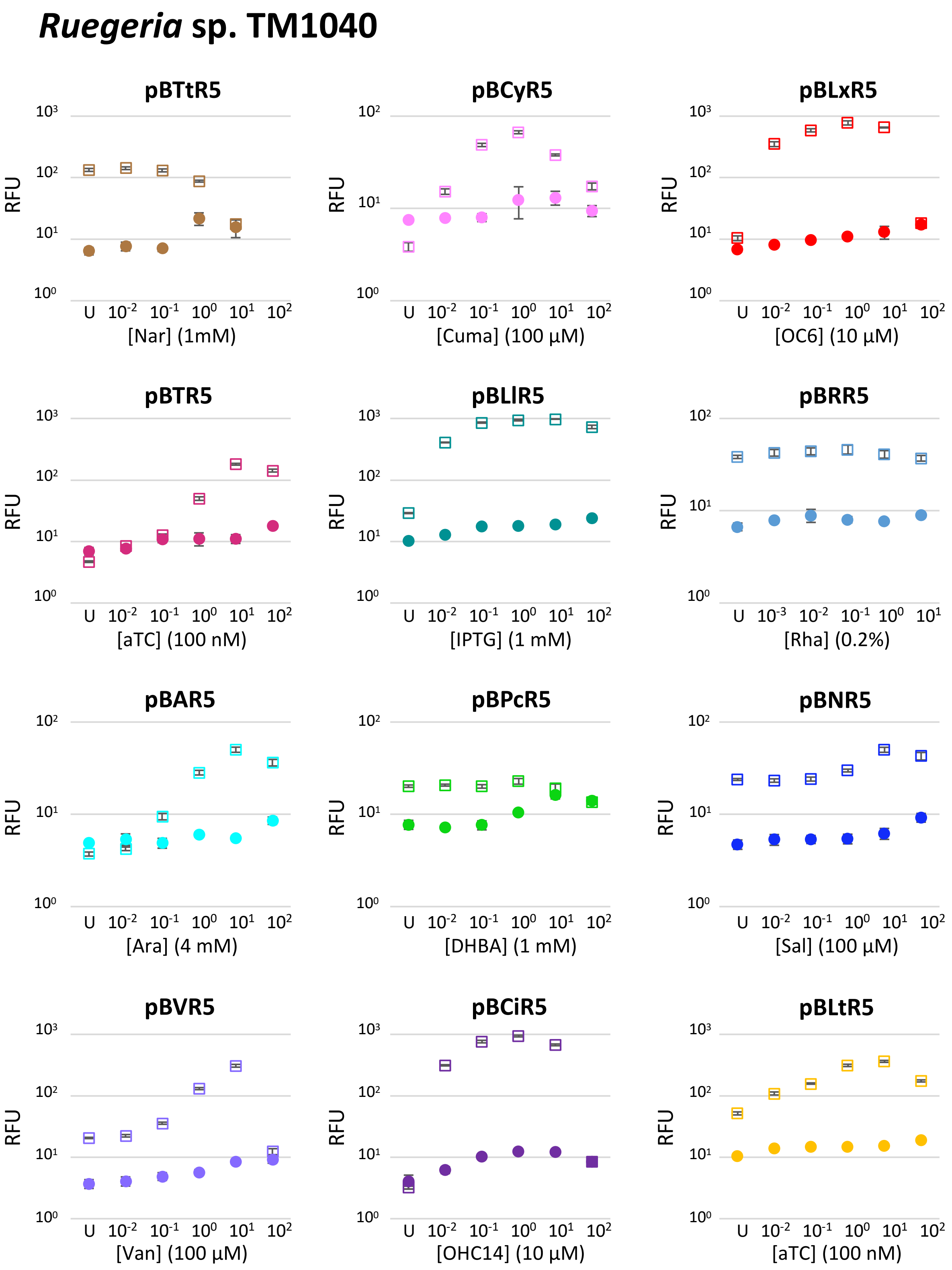

Ruegeria sp. TM1040

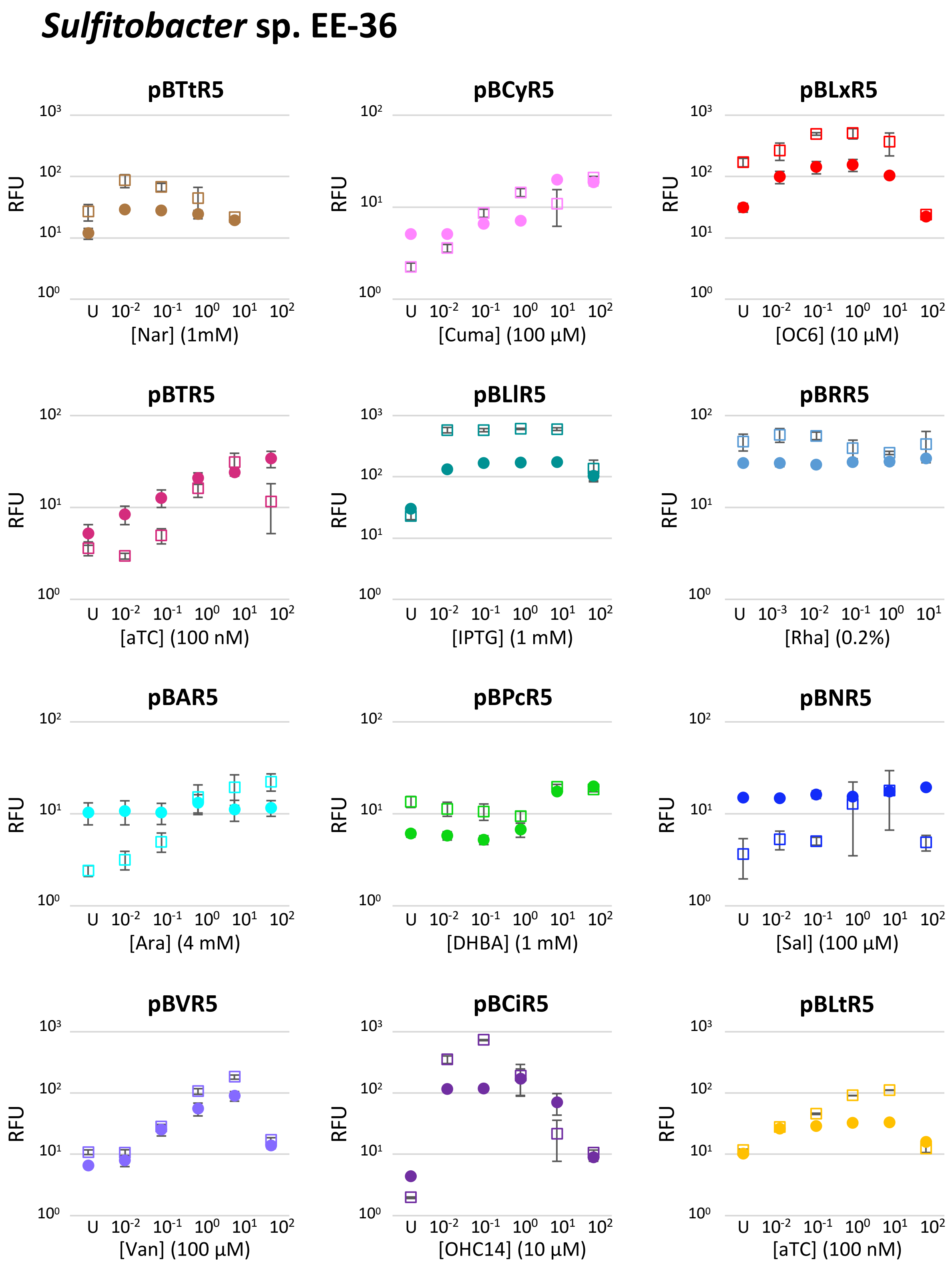

Sulfitobacter sp. EE-36

A. fischeri

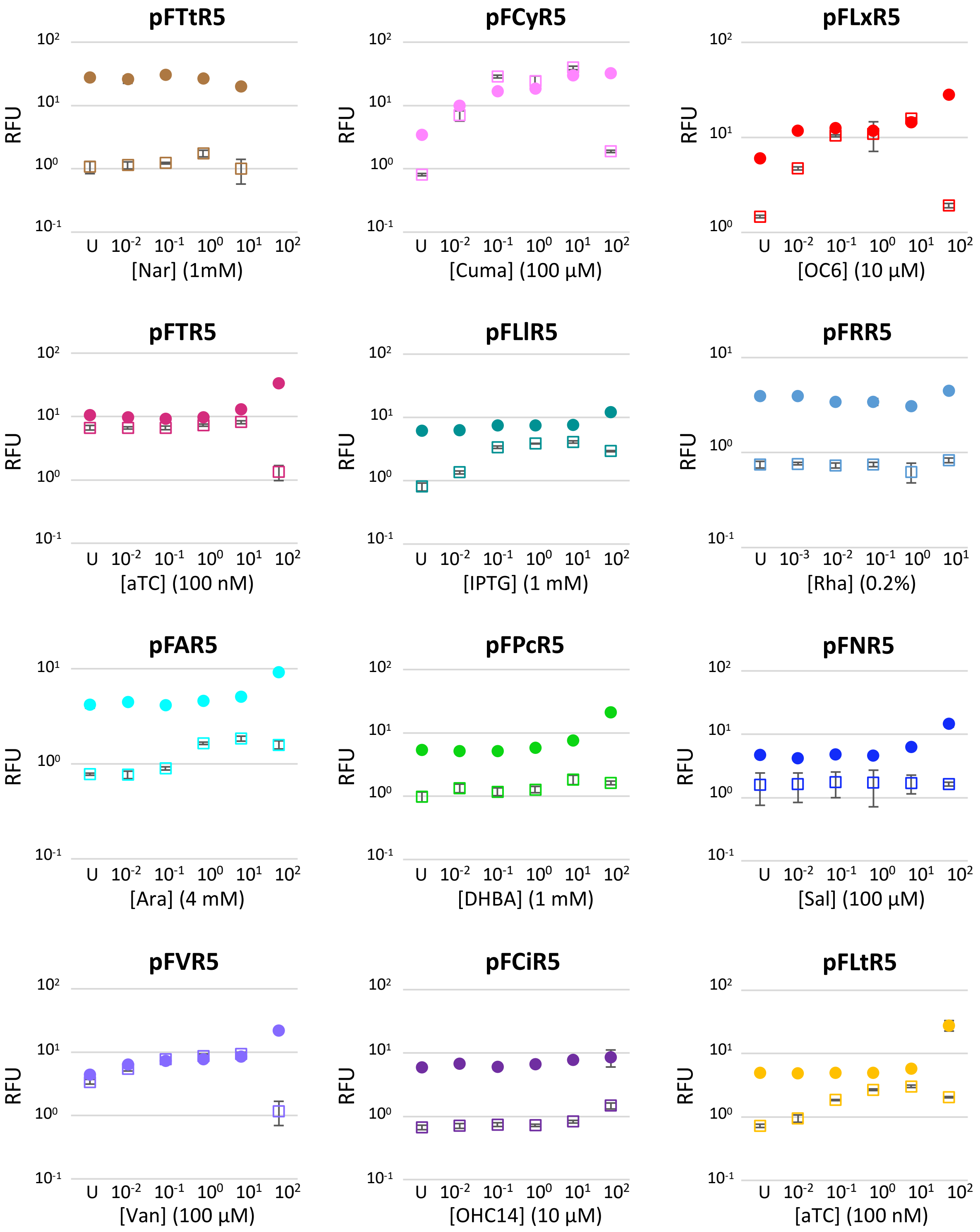

A. fischeri

Supplementary Note 5: Library Construction

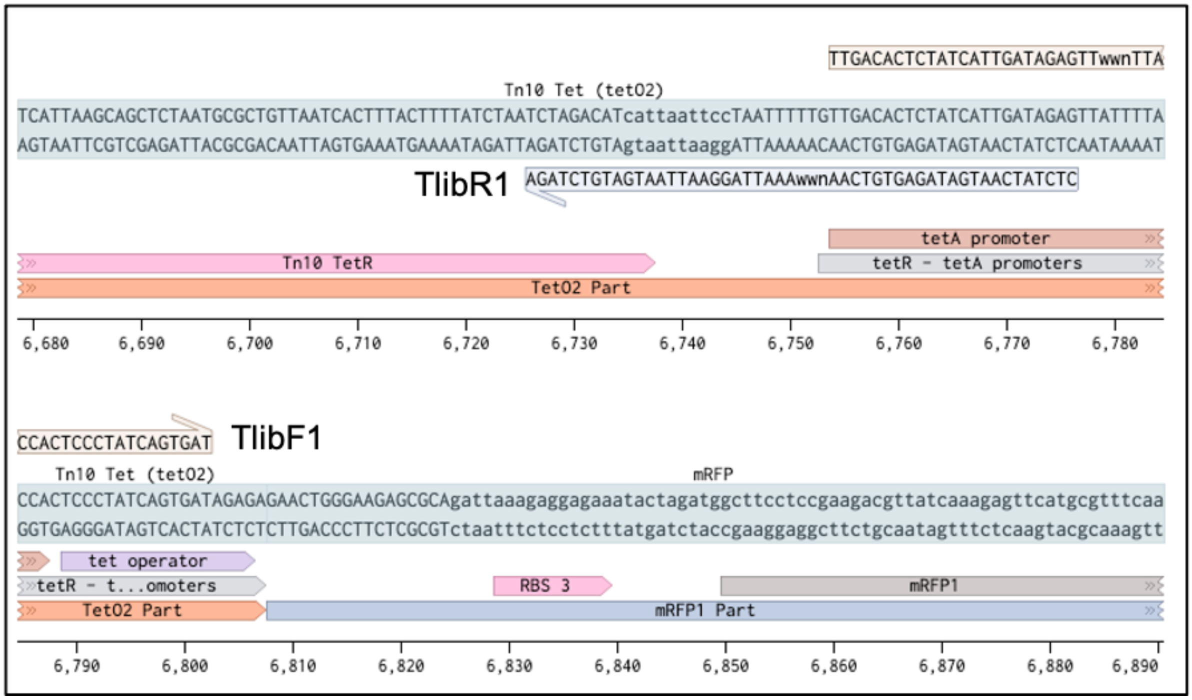

pFTR5 library assembly

Part amplification for library assembly included standard primers to amplify standard parts and non-standard library primers to create parts that included degeneracies. Non-standard library primers are labeled in the figure above and shown in the table below; degeneracies are underlined. Original plasmid pFTR5 was used as the template.

Library Primers	
TlibF1	ttgacactctatcattgatagagttwwnttaccactccctatcagtgat
TlibR1	ctctatcaatgatagagtgtcaanwwaaattaggaattaatgatgtctaga

Here, primer pairs TlibF1-reR and TlibR1-prF were used to create the variant pieces. The origin was amplified with standard primers orF and orR and the marker with amF and amR. A four-piece assembly was performed following protocols in Supplementary Note 2.

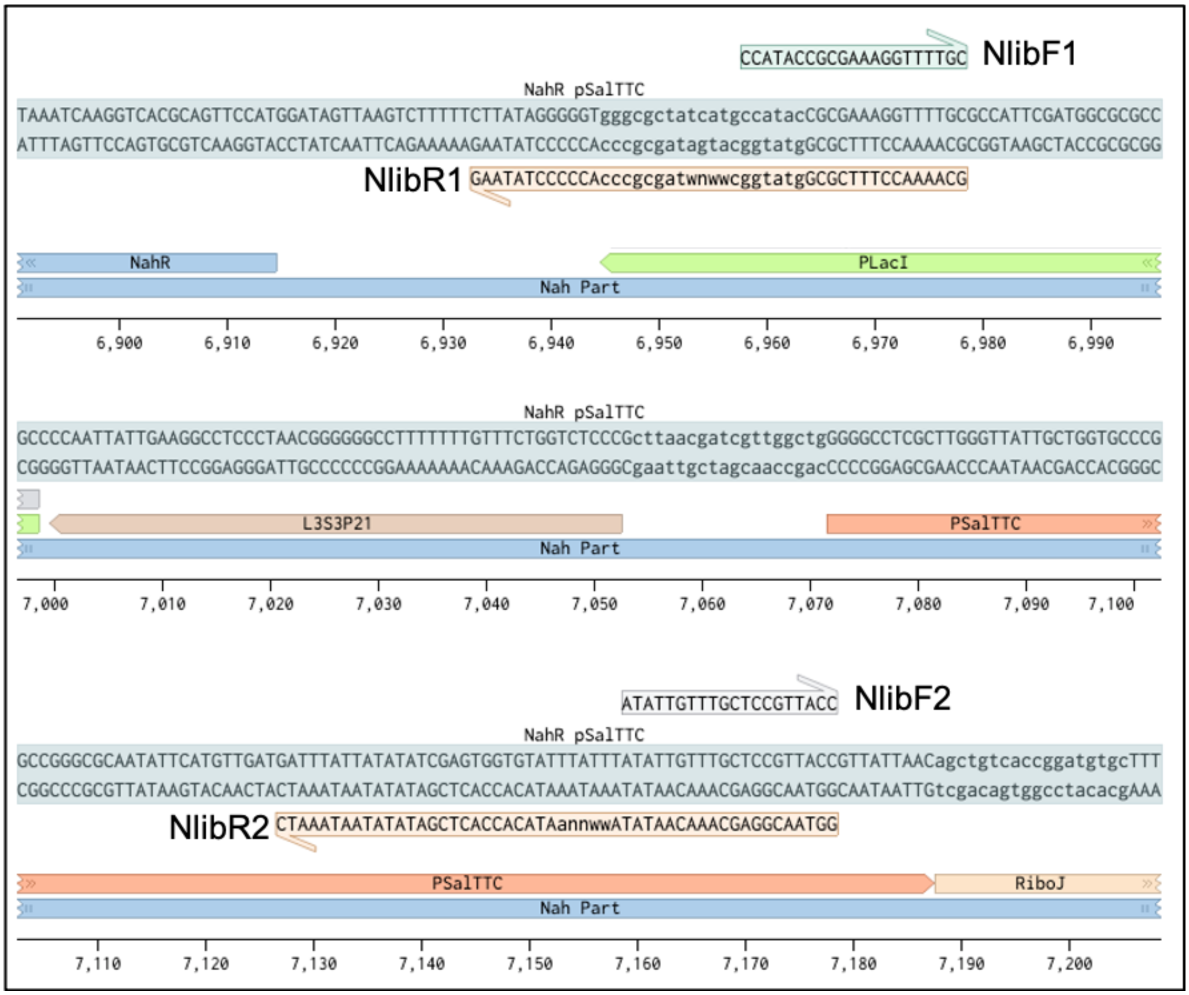

pFNR5 library assembly

Part amplification for library assembly includes both standard primers to amplify whole parts and non-standard library primers to create parts with added degeneracies. Non-standard library primers are labeled in the figure above and shown in the table below; degeneracies are underlined. Original plasmid pFNR5 is used as the template.

Library Primers	
NlibF1	ccataccgcgaaaggttttgc
NlibR1	gcaaaacctttcgcggtatggcwwnwtagcgcccaccccctataag
NlibF2	atattgtttgctccgttacc
NlibR2	ggtaacggagcaaacaatatawwnnaatacaccactcgatatataataaatc

Here, primer pairs NlibR1-prF, NlibF2-reR, and NlibF1-NlibR2 amplify library-specific parts. The origin was amplified with standard primers orF and orR and the marker with amF and amR. The five-piece assembly was performed following protocols in Supplementary Note 2.

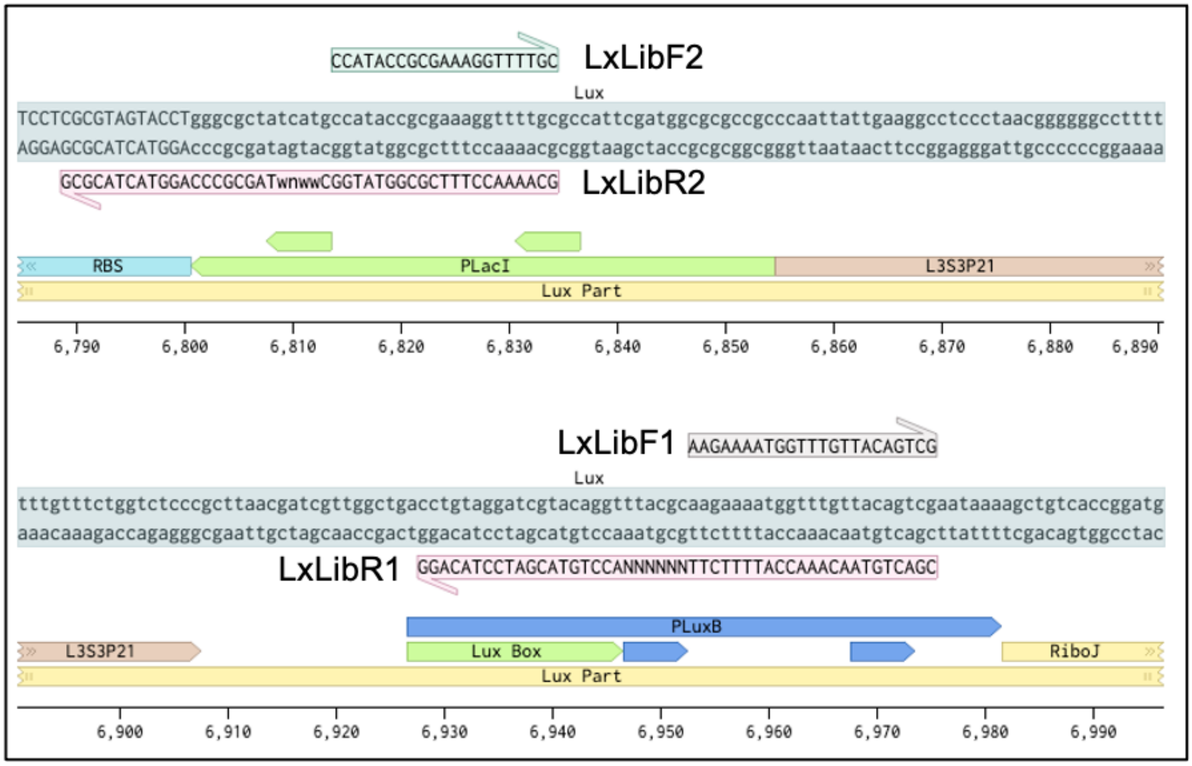

pFLxR5 library assembly

Part amplification for library assembly includes both standard primers to amplify whole parts and non-standard library primers to create parts with added degeneracies. Non-standard library primers are labeled in the figure above and shown in the table below; degeneracies are underlined. The degenerate P_{LuxB} library uses the original plasmid pFLxR5 as a template. The library with degeneracies in P_{LacI} is built using the P_{LuxB} library as the template, resulting in a library of degeneracies in both the regulator and regulated promoters.

Library Primers	
LxlibF1	aagaaaatggtttgttacagtcg
LxlibR1	cgactgtaacaaaccattttcttnnnnnnacctgtacgatcctacagg
LxlibF2	ccataccgcgaaaggttttgc
LxlibR2	gcaaaacctttcgcggtatggcwwnwtagcgcccaggtactacgcg

pFLxR5 library was constructed first constructed as a P_{LuxB} degenerate library assembled from three parts. The first part was amplified with primer LxlibF1 paired with standard primer reR, the second part was amplified with standard primer orF paired with LxlibR1, and the third part amplified with standard primers amF and amR. Using this library as template the parts were amplified using primer pairs LxlibF2 with standard primer amR, LxlibR2 with standard primer prF, and orF with orR. All reactions were performed following protocols in Supplementary Note 2.

Supplementary Note 6: Violacein Pathway Expression Experiments

While fluorescence offers an adequate measurement of induced expression, background fluorescence from a rich growth medium can mask low expression levels. For this reason, we were interested in an alternative reporter that still allowed for quick measurements that were directly comparable across our strains. We chose to use the violacein production pathway, an antibacterial compound from *Pseudoalteromonas luteoviolacea* that is the product of a five gene operon (4). A water-insoluble pigment, violacein can be easily extracted from cells and measured via absorbance in a spectrophotometer (26). To test whether this was a viable alternative to mRFP in our strains, we cloned violacein into three plasmid backbones and measured uninduced violacein production over time in *P. aeruginosa* and found that it followed the same rank order as mRFP.

While these results suggest a linear relationship between inducible system leakiness and violacein output, that relationship breaks down when systems are induced to high levels of expression. Unlike fluorescent proteins, which are largely disconnected from cellular metabolism, violacein is built from tryptophan and likely presents a metabolic burden on the cell when it is highly expressed (26). Accordingly, we saw lower levels of violacein from induced systems than from those that were never induced (data not shown) and proceeded to use this reporter only as a measure of basal expression. Four members of the 2-promoter pFLxR5 library in *P. aeruginosa* had the violacein pathway cloned in place of the mRFP reporter. All four of these isolates had much lower levels of violacein production than the original plasmid. To verify that these isolates were still functional and inducible to a level comparable to the original pFLxR5 plasmid, we replaced violacein with mRFP once more and screened for induced expression. All four isolates turned on to a level comparable to the original vector but were up to 194-fold less leaky at the 24-hour timepoint. These results show that violacein expression is an effective way to sensitively measure basal expression and add support to the library technique's effectiveness to find mutants with an improved dynamic range of expression.

P. aeruginosa

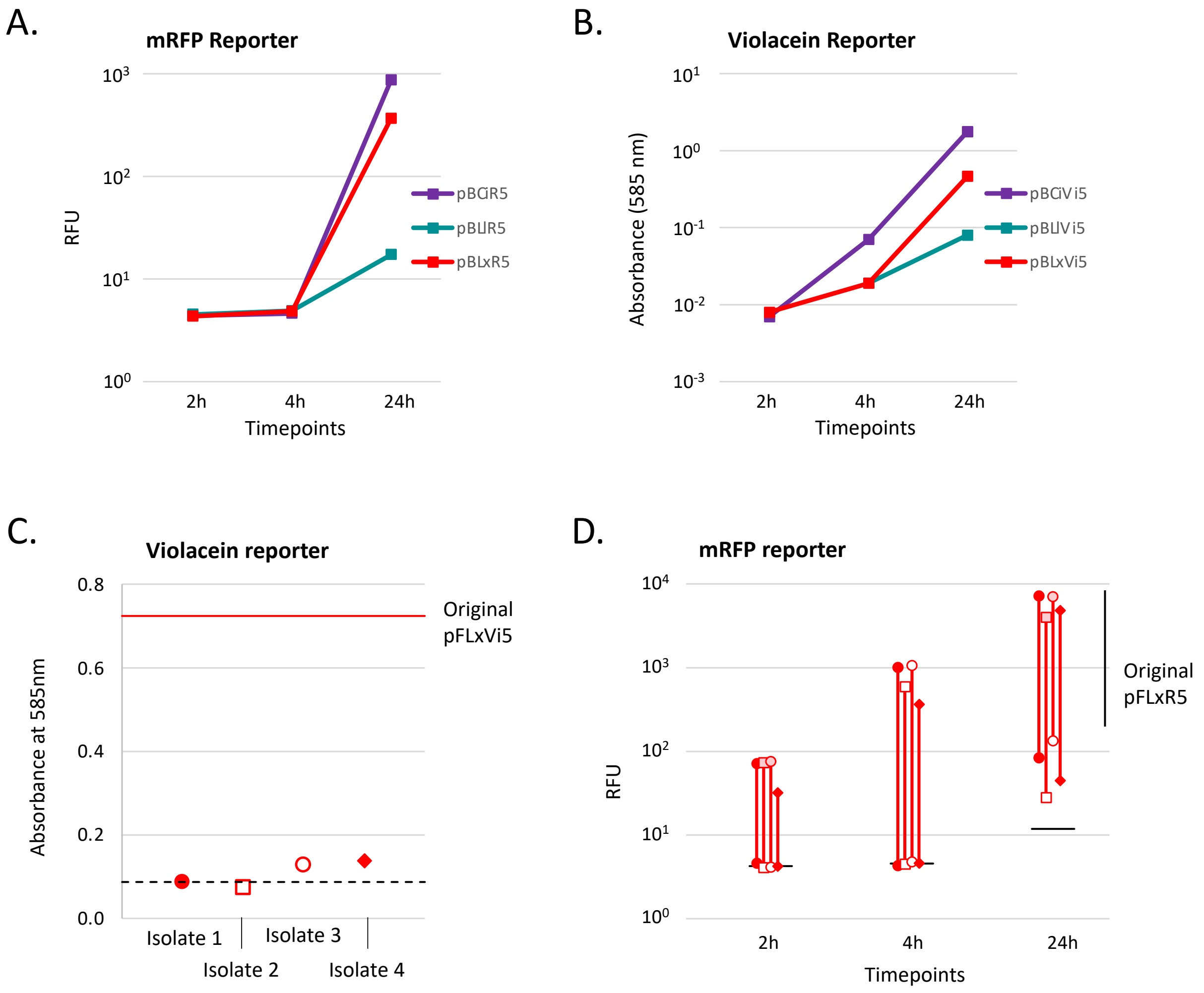

Comparing violacein production to mRFP expression in *P. aeruginosa*.
Uninduced expression of mRFP (A.) compared to violacein pathway (B.). Three inducible systems were screened in *P. aeruginosa* using mRFP as a reporter or violacein. Systems remained uninduced for the duration of the three timepoints at 2 h, 4 h, and 24 h. Fluorescence data is raw RFU and background absorbance was subtracted from the violacein dataset. LuxR/P_{LuxB} library isolates in *P. aeruginosa* screened with violacein (C.) and mRFP reporter (D.). C. Absorbance at 585 nm of four pFLxVi5 library isolates after violacein extraction grown in the absence of inducer overnight. Absorbance of the uninduced control strain (pFLxVi5 non-mutant) shown with a dashed line. D. Same four mutants in C. with mRFP as a reporter. Vertical lines represent expression in the presence and absence of inducer at three timepoints, 2 h, 4 h, and 24 h. Range of expression of original pFLxR5 strain represented by the shaded box, fluorescence of empty vector control is shown as black horizontal lines. All data is the average of three replicates.

Supplementary References

1. Kovach,M.E., Elzer,P.H., Hill,D.S., Robertson,G.T., Farris,M.A., Roop,R.M. and Peterson,K.M. (1995) Four new derivatives of the broad-host-range cloning vector pBBR1MCS, carrying different antibiotic-resistance cassettes. *Gene*, **166**, 175–176.
2. Segall-Shapiro,T.H., Sontag,E.D. and Voigt,C.A. (2018) Engineered promoters enable constant gene expression at any copy number in bacteria. *Nat. Biotechnol.*, **36**, 352–358.
3. Kaczmarczyk,A., Vorholt,J.A. and Francez-Charlot,A. (2013) Cumate-inducible gene expression system for sphingomonads and other Alphaproteobacteria. *Appl. Environ. Microbiol.*, **79**, 6795–6802.
4. Zhang,J.J., Tang,X., Zhang,M., Nguyen,D. and Moore,B.S. (2017) Broad-host-range expression reveals native and host regulatory elements that influence heterologous antibiotic production in Gram-negative bacteria. *MBio*, **8**, 1–16.
5. Choi,K.H., Gaynor,J.B., White,K.G., Lopez,C., Bosio,C.M., Karkhoff-Schweizer,R.A.R. and Schweizer,H.P. (2005) A Tn7-based broad-range bacterial cloning and expression system. *Nat. Methods*, **2**, 443–448.
6. Held,D., Yaeger,K. and Novy,R. (2003) New coexpression vectors for expanded compatibilities in E. coli. *Innovations*.
7. Dunn,A.K., Millikan,D.S., Adin,D.M., Bose,J.L. and Stabb,E. V. (2006) New rfp- and pES213-derived tools for analyzing symbiotic Vibrio fischeri reveal patterns of infection and lux expression in situ. *Appl. Environ. Microbiol.*, **72**, 802–810.
8. Thomason,L.C., Sawitzke,J.A., Li,X., Costantino,N. and Court,D.L. (2014) Recombineering: Genetic engineering in bacteria using homologous recombination.
9. Kim,J., Webb,A.M., Kershner,J.P., Blaskowski,S. and Copley,S.D. (2014) A versatile and highly efficient method for scarless genome editing in Escherichia coli and Salmonella enterica. *BMC Biotechnol.*, **14**, 1–13.
10. Meyer,A.J., Segall-Shapiro,T.H., Glassey,E., Zhang,J. and Voigt,C.A. (2019) Escherichia coli "Marionette" strains with 12 highly optimized small-molecule sensors. *Nat. Chem. Biol.*, **15**, 196–204.
11. Khlebnikov,A., Datsenko,K.A., Skaug,T., Wanner,B.L. and Keasling,J.D. (2001) Homogeneous expression of the PBAD promoter in Escherichia coli by constitutive expression of the low-affinity high-capacity araE transporter. *Microbiology*, **147**, 3241–3247.
12. Chen,Y., Kim,J.K., Hirning,A.J., Josić,K. and Bennett,M.R. (2015) Emergent genetic oscillations in a synthetic microbial consortium. *Science (80-.).*, **349**, 986–989.
13. Lithgow,J.K., Wilkinson,A., Hardman,A., Rodelas,B., Wisniewski-Dyé,F., Williams,P. and Downie,J.A. (2000) The regulatory locus cinRI in Rhizobium leguminosarum controls a network of quorum-sensing loci. *Mol. Microbiol.*, **37**, 81–97.
14. Choi,Y.J., Morel,L., François,T. Le, Bourque,D., Lucie,B., Groleau,D., Massie,B. and Miguez,C.B. (2010) Novel, versatile, and tightly regulated expression system for Escherichia coli strains. *Appl. Environ. Microbiol.*, **76**, 5058–5066.
15. Stanton,B.C., Nielsen,A.A.K., Tamsir,A., Clancy,K., Peterson,T. and Voigt,C.A. (2014) Genomic mining of prokaryotic repressors for orthogonal logic gates. *Nat. Chem. Biol.*, **10**, 99–105.

16. Moon,T.S., Lou,C., Tamsir,A., Stanton,B.C. and Voigt,C.A. (2012) Genetic programs constructed from layered logic gates in single cells. *Nature*, **491**, 249–253.
17. Salis,H.M., Mirsky,E.A. and Voigt,C.A. (2009) Automated design of synthetic ribosome binding sites to control protein expression. *Nat. Biotechnol.*, **27**, 946–950.
18. Schell,M.A. and Poser,E.F. (1989) Demonstration, characterization, and mutational analysis of NahR protein binding to nah and sal promoters. *J. Bacteriol.*, **171**, 837–846.
19. Jha,R.K., Kern,T.L., Fox,D.T. and Strauss,C.E.M. (2014) Engineering an Acinetobacter regulon for biosensing and high-throughput enzyme screening in E. coli via flow cytometry. *Nucleic Acids Res.*, **42**, 8150–8160.
20. Molina-Henares,A.J., Krell,T., Eugenia Guazzaroni,M., Segura,A. and Ramos,J.L. (2006) Members of the IclR family of bacterial transcriptional regulators function as activatorsand/or repressors. *FEMS Microbiol. Rev.*, **30**, 157–186.
21. Lutz,R. and Bujard,H. (1997) Independent and tight regulation of transcriptional units in Escherichia coli via the LacR/O, the TetR/O and AraC/I1-I2 regulatory elements. *Nucleic Acids Res.*, **25**, 1203–1210.
22. Rogers,J.K., Guzman,C.D., Taylor,N.D., Raman,S., Anderson,K. and Church,G.M. (2015) Synthetic biosensors for precise gene control and real-time monitoring of metabolites. *Nucleic Acids Res.*, **43**, 7648–7660.
23. Tera,W., Felipe,A., Segura,A., Rojas,A. and Ramos,J. (2003) Antibiotic-Dependent Induction of Pseudomonas putida DOT-T1E TtgABC Efflux Pump Is Mediated by the Drug Binding Repressor TtgR. *Antimicrob. Agents Chemother.*, **47**, 3067–3072.
24. Kaczmarczyk,A., Vorholt,J.A. and Francez-Charlot,A. (2014) Synthetic vanillate-regulated promoter for graded gene expression in Sphingomonas. *Sci. Rep.*, **4**, 4–7.
25. Kunjapur,A.M. and Prather,K.L.J. (2019) Development of a Vanillate Biosensor for the Vanillin Biosynthesis Pathway in E. coli. *ACS Synth. Biol.*, **8**, 1958–1967.
26. Jones,J.A., Vernacchio,V.R., Lachance,D.M., Lebovich,M., Fu,L., Shirke,A.N., Schultz,V.L., Cress,B., Linhardt,R.J. and Koffas,M.A.G.G. (2015) ePathOptimize: A combinatorial approach for transcriptional balancing of metabolic pathways. *Sci. Rep.*, **5**, 1–10.
